# Ancient Evolution and Dispersion of Human Papillomavirus 58 Variants

**DOI:** 10.1128/JVI.01285-17

**Published:** 2017-10-13

**Authors:** Zigui Chen, Wendy C. S. Ho, Siaw Shi Boon, Priscilla T. Y. Law, Martin C. W. Chan, Rob DeSalle, Robert D. Burk, Paul K. S. Chan

**Affiliations:** a Department of Microbiology, Faculty of Medicine, The Chinese University of Hong Kong, Hong Kong SAR, China; b Stanley Ho Center for Emerging Infectious Diseases, Faculty of Medicine, The Chinese University of Hong Kong, Hong Kong SAR, China; c Sackler Institute of Comparative Genomics, American Museum of Natural History, New York, New York, USA; d Departments of Pediatrics, Microbiology and Immunology, Epidemiology and Population Health, and Obstetrics, Gynecology, and Women's Health, Albert Einstein College of Medicine, Bronx, New York, USA; University of Texas Southwestern Medical Center

**Keywords:** papillomavirus, cervical cancer, HPV58, evolution, virus-host codivergence, oncogenicity

## Abstract

Human papillomavirus 58 (HPV58) is found in 10 to 18% of cervical cancers in East Asia but is rather uncommon elsewhere. The distribution and oncogenic potential of HPV58 variants appear to be heterogeneous, since the E7 T20I/G63S variant is more prevalent in East Asia and confers a 7- to 9-fold-higher risk of cervical precancer and cancer. However, the underlying genomic mechanisms that explain the geographic and carcinogenic diversity of HPV58 variants are still poorly understood. In this study, we used a combination of phylogenetic analyses and bioinformatics to investigate the deep evolutionary history of HPV58 complete genome variants. The initial splitting of HPV58 variants was estimated to occur 478,600 years ago (95% highest posterior density [HPD], 391,000 to 569,600 years ago). This divergence time is well within the era of speciation between Homo sapiens and Neanderthals/Denisovans and around three times longer than the modern Homo sapiens divergence times. The expansion of present-day variants in Eurasia could be the consequence of viral transmission from Neanderthals/Denisovans to non-African modern human populations through gene flow. A whole-genome sequence signature analysis identified 3 amino acid changes, 16 synonymous nucleotide changes, and a 12-bp insertion strongly associated with the E7 T20I/G63S variant that represents the A3 sublineage and carries higher carcinogenetic potential. Compared with the capsid proteins, the oncogenes E7 and E6 had increased substitution rates indicative of higher selection pressure. These data provide a comprehensive evolutionary history and genomic basis of HPV58 variants to assist further investigation of carcinogenic association and the development of diagnostic and therapeutic strategies.

**IMPORTANCE** Papillomaviruses (PVs) are an ancient and heterogeneous group of double-stranded DNA viruses that preferentially infect the cutaneous and mucocutaneous epithelia of vertebrates. Persistent infection by specific oncogenic human papillomaviruses (HPVs), including HPV58, has been established as the primary cause of cervical cancer. In this work, we reveal the complex evolutionary history of HPV58 variants that explains the heterogeneity of oncogenic potential and geographic distribution. Our data suggest that HPV58 variants may have coevolved with archaic hominins and dispersed across the planet through host interbreeding and gene flow. Certain genes and codons of HPV58 variants representing higher carcinogenic potential and/or that are under positive selection may have important implications for viral host specificity, pathogenesis, and disease prevention.

## INTRODUCTION

Cervical cancer is one of the leading causes of cancer mortality in women worldwide, with more than 0.2 million deaths annually (1). Persistent infection with high-risk genital human papillomaviruses (HPVs) is the root cause of cervical cancer (2, 3). HPVs are ancient DNA viruses that have evolved over millions of years, reaching a high level of diversity at the genomic and phenotypic levels (4, 5). The Alphapapillomavirus genus is associated with infections of the anogenital and oral mucosa and contains all high-risk types, among which HPV16 and HPV18 account for ∼70% of all cervical cancers across the world. Several high-risk HPV types have shown geographical/ethnic differences in disease attribution. Of particular interest, HPV58 ranks sixth or seventh as a cause of cervical cancer globally, but it is the third most predominant type in East Asia and was found in 10 to 18% of cervical cancers and precancers (6–8). However, the reason for this remarkable geographic distribution of HPV58-associated cervical disease burden is unknown.

HPV types are defined as those with L1 genomic sequences differing by >10%, whereas variant lineages and sublineages differ by 1 to 10% and 0.5 to 1% over the complete genomes, respectively (9, 10). At both the type and variant levels, substantial differences in evolutionary relatedness and carcinogenicity have been observed. For example, HPV16 variants were shown to correlate with the continental distribution of humans and were associated with a different degree of cancer risk (11, 12). In a multicontinental epidemiological study, the HPV58 E7 T20I/G63S variant was more frequently detected in East Asia and carried a 7- to 9-fold-higher risk for cervical cancer, indicating that the viral variant plays a key role in modulating its unique carcinogenic property (13). These data strongly indicate that sequence variation at certain sites of the HPV genome can critically determine phenotypic characteristics, including oncogenicity.

Evolutionary relationships between micropathogens and their hosts are often complex, with multiple time and space scales over which a phylodynamic interaction captures the relationships among pathogen genetic diversity, host immunity, and pathogen transmission (14–16). A prevailing model suggesting virus-host codivergence has been shown for feline papillomaviruses within the genus *Lambdapapillomavirus* isolated from oral lesions (17), but the evolution of papillomaviruses does not mirror the host phylogeny accurately (18). While natural selection and genomic drift drive important forces shaping the wide variety of papillomavirus (PV) phylogenies, potential crucial mechanisms for the well-adapted spectrum of these slowly evolving double-stranded DNA viruses (e.g., papillomaviruses or polyomaviruses) may include host switching, niche adaptation, lineage sorting and duplication, or rare recombination (18–21). A common assumption of HPV evolution was that viruses have codiverged with modern humans as a host population and distributed across the planet through Homo sapiens migration (22, 23), but this scenario is being challenged by the differential coevolution of HPV16 lineages with archaic hominins (24). Understanding the capacity for, and history of, viral transmission and adaptation to host immune selection and epidemic dynamics will facilitate the clarification of how virus genetic variation determines phylogeny and pathogenicity in individual hosts and the population.

In this study, we applied phylogenetic and bioinformatics analyses on a large data set of HPV58 variants to understand viral genetic variation and evolutionary dynamics. By estimating divergence times, we determined a complex evolutionary history of HPV58 variants that involves ancient codivergence with archaic hominins and recent viral transmission from Neanderthals/Denisovans to modern human populations. By examining viral gene substitutions and genomic signatures, we identified certain genes and mutations that process positive selection; some of them may represent higher carcinogenic potential. These findings will have clinical implications for screening, early intervention, and therapeutics in the future.

## RESULTS

### HPV58 variant genomic diversity and phylogeny.

**FIG 1:**
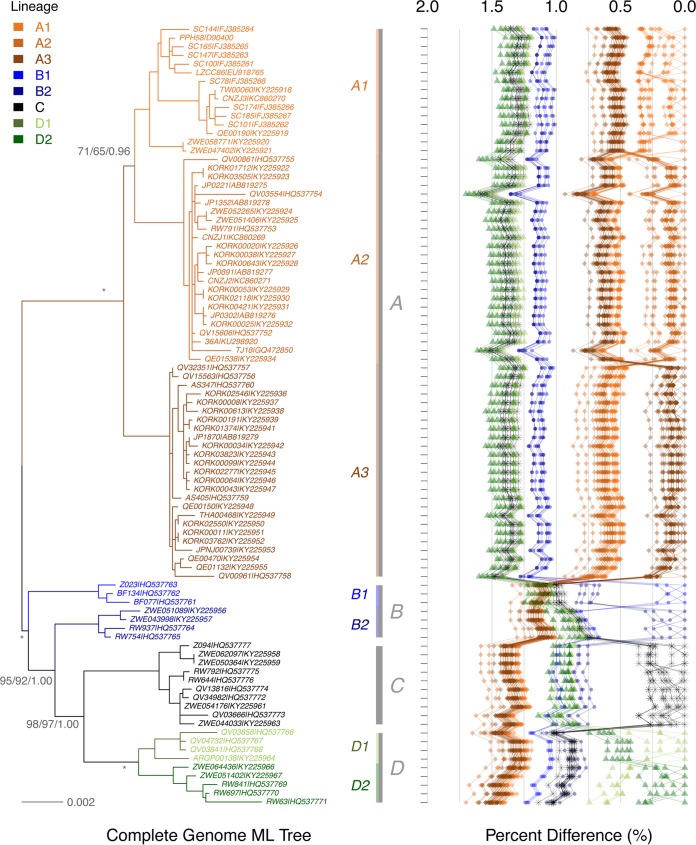
Phylogeny of HPV58 complete genomes. The topology was obtained from the maximum likelihood tree by using RAxML, inferred from a global alignment of 90 complete genomes. Support scores alongside the branches of each sublineage indicate bootstrap percentages obtained by RAxML and PhyML and the Bayesian credibility values obtained by MrBayes. The stars indicate absolute agreement among the results of the three algorithms. The pairwise nucleotide sequence differences were calculated for each isolate and are shown on the right, with the scale displayed on the top. Values for each comparison for a given isolate are connected by lines, and the comparison to self is indicated as 0.0%.

Complete genome analysis using multiple phylogenetic algorithms clustered 90 HPV58 isolates into four lineages that were further divided into seven sublineages (Fig. 1; see also Table S1 in the supplemental material). The maximum inter(sub)lineage difference was observed between A2 and D2 variants (mean percentage ± standard error, 1.45% ± 0.13%), and the overall interlineage and intersublineage mean differences ranged between 0.90% and 1.38% and between 0.54% and 0.75%, respectively. When A and non-A (B, C, and D) variants were compared, the latter encompassed a higher level of genetic diversity (nucleotide diversity [π] of 0.00748 versus 0.00439).

A summary of the sequence diversity of 90 complete genomes is shown in Table 1. In total, 500 sites (6.4%) have changed across the complete genome, among which 202 codon sites (8.4%) within 8 open reading frames (ORFs) were nonsynonymous. The noncoding regions of HPV58 (the long control region [LCR], noncoding region 1 [NCR1], and NCR2) and E4/E5 ORFs showed higher levels of genomic diversity, while the L1 protein (amino acid change) was relatively conserved compared to other ORFs.

**TABLE 1:** Variation of HPV58 genome regions and ORFs^*e*^

ORF or region	Maximum nucleotide pairwise difference (%)	Length of nucleotide sequence alignment (bp)	No. of variable nucleotide positions^*a*^	% of variable nucleotide positions	No. of variable nucleotide changes at each codon position^*b*^			Maximum amino acid pairwise difference (%)	Length of amino acid alignment	No. of variable amino acid positions^*c*^	% of variable amino acid positions	Ratio of nonsynonymous to synonymous changes
					1st	2nd	3rd					
E6	1.8	447	16	3.6	8	1	7	4.0	148	8	5.4	1.33
E7	3.4	294	20	6.8	7	6	7	7.1	97	12	12.4	1.71
E1	1.3	1,932	89	4.6	35	17	37	2.0	645	46	7.1	1.12
E2	1.3	1,074	53	4.9	11	9	33	2.2	357	27	7.6	1.13
E4	2.2	273	19	7.0	5	10	4	5.6	90	17	18.9	17.00
NCR1	3.2	62	4	6.5								
E5	3.5	228	19	8.3	8	0	11	4.0	75	5	6.7	0.36
NCR2	5.8	122	18	14.8								
L2	2.5	1,419	103	7.3	24	25	53	3.4	472	52	11.0	1.24
L1	2.2	1,572	94	6.0	17	15	62	3.6	523	35	6.7	0.67
LCR	3.6	849	92	10.8								
CG/8 ORFs^*d*^	1.7	7,834	500	6.4	115	83	214	2.2	2,407	202	8.4	1.08

a Each insert or deletion event was counted as one variation.

b The first, second, and third nucleotide positions in a codon.

c Amino acid changes (nonsynonymous changes). Each insert or deletion event was counted as one variation.

d Each nucleotide position is counted once based on 90 complete genome alignments.

e CG, complete genome; NCR1, noncoding region 1 between the E2 and E5 ORFs; NCR2, noncoding region 2 between the E5 and L2 ORFs; LCR, long control region.

No evidence of recombination was found across the HPV58 complete genome by a genetic algorithm for recombination detection (GARD). However, phylogenetic incongruence was observed among A variants when separated maximum likelihood (ML) trees inferred from the early genes (E1, E2, E6, and E7) and the late genes (L1 and L2) were compared (trees are not shown). The A1 sublineage was closer to A2 in both early gene (86% bootstrap value) and complete genome trees (Fig. 1) but closer to A3 in the late gene tree (73% bootstrap value). A bootstrap scanning method with a window size of 1,000 bp using SimPlot identified potential breakpoints among the E1, E2, L2, and L1 genes within the A variants (data not shown). Either raw sequences or sequences filtered by removing mutations potentially due to the antiviral activity of human APOBEC3 (hA3) cytosine deaminases showed similar topology incongruences between early and later gene trees.

Although sampling of isolates increased the repertoire of genomic variability, our data may have covered the majority of variant lineages within the given populations since the rarefaction curves of parsimony-informative single nucleotide polymorphisms (SNPs) (site detected in ≥2 samples) leveled off with increasing numbers of genomes. However, additional variations, including potential false-positive SNPs or sequencing errors, remain unavoidable, as the number of singleton SNPs (variations present only once in the sampled genomes) increased almost linearly following the increase in sample size (data not shown).

### Lineage fixation and genomic signatures associated with E7 T20I/G63S.

**FIG 2:**
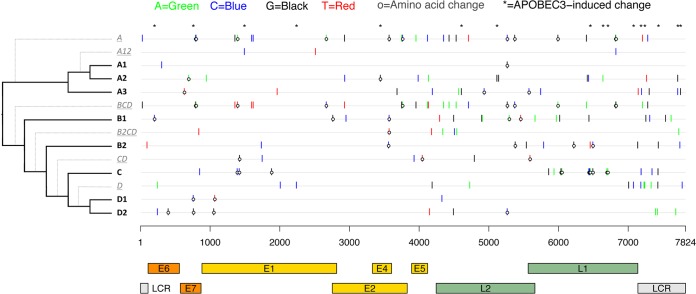
HPV58 lineage- and sublineage-specific nucleotide and amino acid changes across the complete genome. The *x* axis shows HPV58 gene/region positions, aligned according to the sublineage in the phylogenetic tree on the *y* axis. Lineage- and sublineage-specific SNPs were determined based on a global alignment of 90 complete genomes and color-coded as shown at the top. Amino acid changes within the E2/E4 region are changes observed in E2. SNPs were cumulative for the underlined lineages traversed from deepest node out to finer subline branches (dotted lines).

Lineage fixation of genetic changes was observed throughout all genes/regions of HPV58 variants. There were at least 141 nucleotide sites and 43 amino acids (aa) specifically fixed on a sublineage, lineage, or certain clade (Fig. 2; see also Table S2 in the supplemental material). For example, the A3 sublineage-specific nucleotide variations of E7 C632T (amino acid T20I); E1 C1965T; E2 A3685G; NCR2 A4192C; L2 G4570A, A4609G, and A4935C (N231T); L2/L1 A5579C (L2 M446L or L1 L5F); L1 T5747C; and LCR G7147T, G7194C, A7304G, A7714C, and A7755G are highly correlated and represent fixed changes in natural selection when sublineage A3 split from its most recent common ancestor (MRCA). The E7 T20I/G63S variant conferring higher risks for cervical precancer and cancer represents the A3 sublineage. A genome-wide association analysis using mutual information (MI) examination identified three additional amino acid changes within L2 (N231T and M446L) and L1 (L5F); 16 synonymous nucleotide mutations within E1, NCR2, L2, L1, and the LCR; and a 12-bp insertion within the LCR as being strongly associated with the E7 T20I/G63S variant (Table 2).

**TABLE 2:**
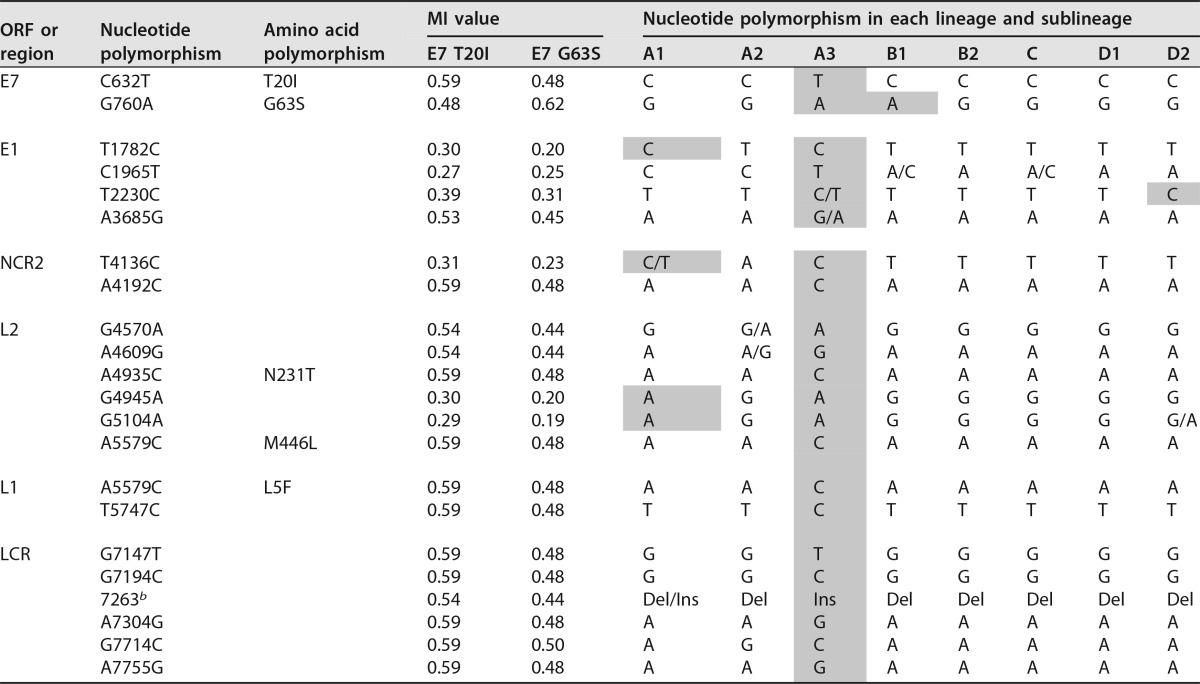
HPV58 single nucleotide polymorphisms showing genomic signatures with the HPV58 A3 variant represented by E7 T20I/G63S^*a*^

a Shading indicates changes identical to those in the A3 variants.

^*b*^ Deletion (Del) or insertion (Ins), TCCTTGTCAGTT (12 bp).

### Natural selection of the HPV58 genome.

In order to determine whether positive selection is playing a role in shaping the genetic makeup of HPV58, six models employing the maximum likelihood regression of codon substitutions (ω = *dN*/*dS*) were applied, and the model with the highest log likelihood value was chosen as the “best” one (Table 3). The ω measure is an average over all sites in an ORF. The E4 gene had the highest average (model 3 [M3]; ω = 1.9), with 5.7% of codons (mean ω = 20.5) under diversifying selection. Statistically significant codon sites in E4 identified by the likelihood ratio test (LRT) were found to be amino acids 1L, 39S, and 74V (*P* ≥ 0.95 by CODEML, and *P* ≥ 0.90 by a fast unconstrained Bayesian approximation [FUBAR]). Although the average *dN*/*dS* ratios of other ORFs were <1, the HPV58 oncogenes E7 (M2, ω = 0.85) and E6 (M3, ω = 0.59) had higher values than did E2 (M8, ω = 0.32), L2 (M3, ω = 0.32), E1 (M3, ω = 0.20), L1 (M3, ω = 0.18), and E5 (M2, ω = 0.07). A total of 11 codon sites (E6 amino acids 46V, 93K, and 97N; E7 amino acids 9R and 63G; E2 amino acid 282V; E4 amino acids 1L, 39S, and 74V; and L1 amino acids 150L and 375T) were demonstrated to be under positive selection. No amino acid within E1, E5, or L2 met criteria for positive selection using the LRT, although several sites (E1 amino acids 79I and 361D and L2 amino acid 433T) had *dN*/*dS* ratios of ≥1 by CODEML.

**TABLE 3:** Likelihood ratio tests for positive selection of amino acid sites for HPV58 genes

ORF	CODEML							FUBAR	
	Best model^*a*^	Log likelihood	*dN/dS* ratio^*b*^	LRT statistic^*c*^	Codon^*d*^	*dN/dS* ratio	Posterior probability^*e*^	*dN/dS* ratio	Posterior probability^*f*^
E6	M3	−779.5126	0.5853	**21.4206**	**46V**	**12.32**	**1.000**	**17.12**	**0.979**
					86D	12.32	1.000	5.57	0.876
					**93K**	**12.32**	**1.000**	**9.25**	**0.947**
					**97N**	**12.32**	**1.000**	**9.54**	**0.949**
E7	M2	−606.8498	0.8527	**16.3474**	**9R**	**10.09**	**0.999**	**8.37**	**0.947**
					**63G**	**10.09**	**1.000**	**20.91**	**0.989**
					64T	8.47	0.833	9.45	0.919
					77V	9.98	0.988	5.20	0.877
E1	M3	−3,452.3510	0.2046	1.8435					
E2	M8	−1,986.1880	0.3243	**23.1108**	**282V**	**30.71**	**1.000**	**20.66**	**0.992**
E4	M3	−629.6729	1.9275	**26.0371**	**1L**	**15.92**	**1.000**	**8.72**	**0.913**
					**39S**	**20.47**	**1.000**	**7.73**	**0.908**
					**74V**	**19.08**	**1.000**	**7.46**	**0.940**
E5	M2	−442.6889	0.0744	0.0003					
L2	M3	−2,918.3903	0.3219	0.0705					
L1	M3	−3,182.2828	0.1830	**17.7128**	**150L**	**5.82**	**0.957**	**19.87**	**0.981**
					325I	5.97	0.984	2.30	0.851
					**375T**	**6.06**	**1.000**	**8.46**	**0.955**

a The “best” model was interpreted from the maximum log-likelihood value. M2, selection; M3, discrete; M8, beta and ω.

b Overall *dN*/*dS* ratio for each gene.

c Likelihood ratio test statistics follow a χ^2^ distribution, with degrees of freedom equaling 2 when values were ≥5.99 and *P* values were ≤0.05 (in boldface type).

d Amino acid sites under positive selection are shown in boldface type.

e Positively selected sites with *P* values of ≥0.950 by CODEML.

f Positively selected sites with *P* values of ≥0.900 by FUBAR.

### Geographic distribution of HPV58 variants.

**FIG 3:**
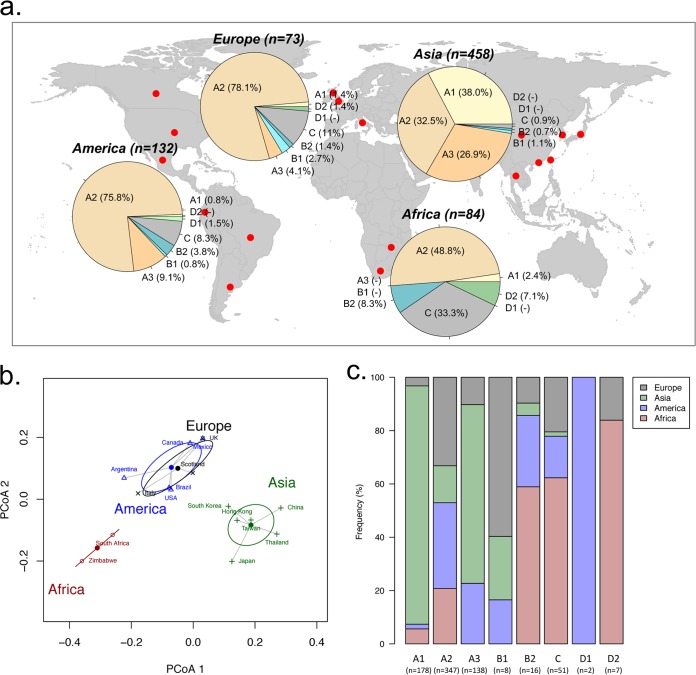
Geographic distribution of HPV58 variants. (a) A total of 747 HPV58 variants with known geographic origins from 16 countries/regions (see details in Table 4) were assigned to a lineage/sublineage and are summarized by continent in the pie charts. (b) Principal-component analysis using a weighted UniFrac algorithm clustered different study cohorts into three distinct groups, mainly matching the geographic locations where the viruses were isolated. (c) Relative frequencies of HPV58 lineage/sublineage distributions in four continents. A higher frequency indicates a predominance of certain lineages/sublineage in the associated geographic area.

Based on SNP patterns and phylogenetic tree topologies, a total of 747 HPV58 variants with known geographic origins from 16 countries/regions was assigned to lineages/sublineages (Table 4). Although these partial sequences spanned variable genes/regions, including E6, E7, L1, and the LCR, we can unambiguously assign each isolate to a phylogenetic branch with maximum likelihood in a complete genome tree using a placement algorithm. As shown in the summarized charts of HPV58 phylogeography, isolates from Asia were equally represented by the A1, A2, and A3 sublineages, with an accumulated prevalence of >97% (Fig. 3a). America and Europe were predominated by A2 variants (76% in America and 78% in Europe) but showed a larger proportion of non-A variants than did Asia (14.4 to 16.5% versus 2.7%). In Africa, about half of the variants were non-A variants, which were predominated by C variants, whereas the A variants were mainly assigned to the A2 sublineage. Overall, the A3 sublineage represented by a high-risk E7 T20I/G63S variant was most common (26.9%) in Asia and, to a lesser extent, was common in America (9.1%) and Europe (4.1%) but was absent in Africa.

**TABLE 4:** Geographic origin of HPV58 variants

Continent	Country or city	Reference	Sequenced region(s)	No. of HPB58 variants								
				Total	A1	A2	A3	B1	B2	C	D1	D2
Africa	South Africa	63	Partial LCR	11	0	6	0	0	5	0	0	0
	Zimbabwe	7	L1, LCR	73	2	35	0	0	2	28	0	6
America	Argentina	7	L1, LCR	7	0	5	0	0	0	0	2	0
	Brazil	63	Partial LCR	61	1	45	6	0	3	6	0	0
	Canada	7	L1, LCR	12	0	11	0	1	0	0	0	0
	Mexico	63	Partial LCR	4	0	4	0	0	0	0	0	0
	USA	7	L1, LCR	39	0	29	4	0	1	5	0	0
		63	Partial LCR	9	0	6	2	0	1	0	0	0
												
Asia	China	7	L1, LCR	3	1	1	1	0	0	0	0	0
		42	Nearly complete genome	37	35	1	1	0	0	0	0	0
		65	Partial E6, E7	22	12	4	6	0	0	0	0	0
		66	E6, E7	135	89	20	24	2	0	0	0	0
	Hong Kong	7	L1, LCR	90	25	36	24	2	1	2	0	0
	Japan	7	L1, LCR	14	1	4	7	0	2	0	0	0
	South Korea	7	L1, LCR	139	9	77	50	1	0	2	0	0
	Taiwan	7	L1, LCR	6	2	1	3	0	0	0	0	0
		63	Partial LCR	5	0	2	3	0	0	0	0	0
	Thailand	7	L1, LCR	7	0	3	4	0	0	0	0	0
Europe	Italy	7	L1, LCR	23	1	16	1	0	0	4	0	1
		64	Partial E6, E7, L1, LCR	24	0	17	1	1	1	4	0	0
	Scotland	63	Partial LCR	7	0	5	1	1	0	0	0	0
	UK	7	L1, LCR	19	0	19	0	0	0	0	0	0

Principal component analysis (PCA) using a weighted UniFrac algorithm led to variants that were well clustered into three distinct groups corresponding to the source of these samples (Africa, Asia, and America/Europe) (Fig. 3b). Globally, the A2 sublineage was the most widespread variant, whereas the A1 and A3 sublineages were rarely founded outside Asia (*P* < 0.001) (Fig. 3c). The majority of HPV58 non-A variants was detectable in Africa compared with other three continents; due to the limited sample size, however, we did not identify the B1 and D1 sublineages in Africa.

### Divergence time estimation and ancestral codon mutation.

**FIG 4:**
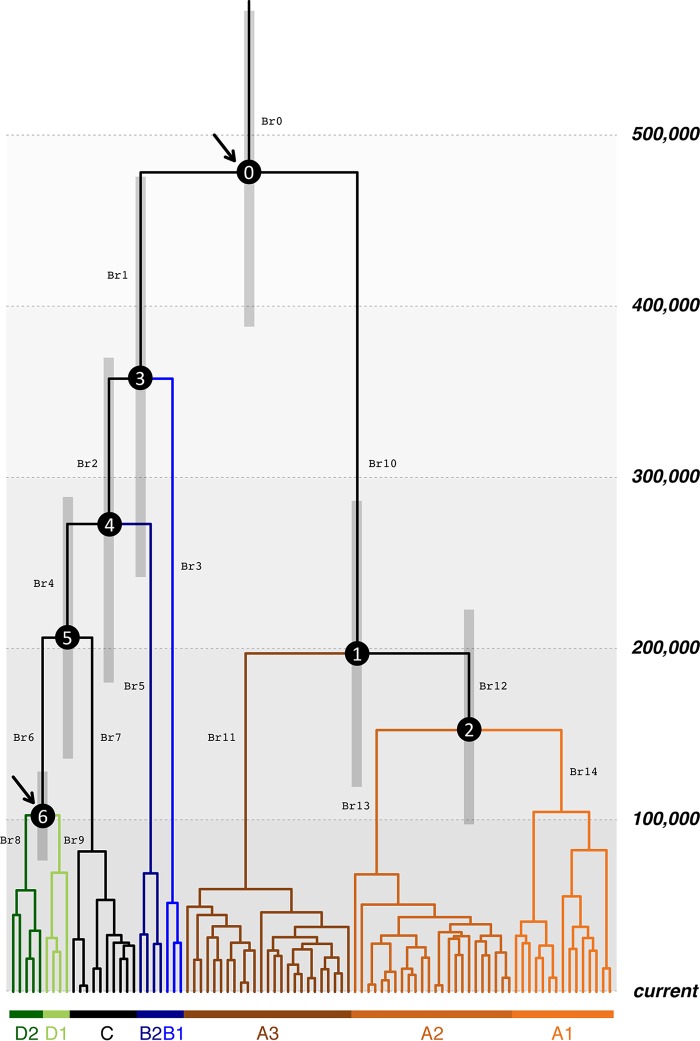
Divergence time estimation for HPV58 variants. A Bayesian MCMC method with a tree prior of a coalescent Bayesian skyline model and a UCLD molecular clock model of rate variation among branches under an HHS scenario, as the best model as determined by AICM (Table 5), was used to calculate the divergence times. An HPV16 variant substitution rate and two human evolutionary time points of calibration (arrowed at nodes 0 and 6) were set. Branch lengths are proportional to the times scaled in thousands of years. Gray bars indicate the 95% HPD for the corresponding divergence age. The branches are coded (Br0 to Br14), and ancestral codon mutations are listed in Table S3 in the supplemental material.

We used a Bayesian Markov chain Monte Carlo (MCMC) framework and the previously reported evolutionary rate of feline papillomaviruses to estimate the HPV58 variant divergence times (Table 5). Papillomaviruses process evolution with a low mutation rate due, in part, to the fact that this kind of double-strand DNA virus uses the host cell DNA replication machinery, characterized by high fidelity, proofreading capacity, and postreplication repair mechanisms. A combination of relaxed log-normal molecular clock and coalescent Bayesian skyline models provided the best performance, with a tree height estimated at 451,600 years ago (451.6 kya) (95% highest posterior density [HPD], 306.0 to 619.6 kya). This estimation is around three times longer than the modern Homo sapiens divergence time (ca. 120 to 180 kya) and implies a hominin host switch (HHS) scenario indicating ancestral viral transmission between archaic and modern human populations. We then used an archaic hominin divergence time (500 kya; 95% HPD, 400 to 600 kya) and a modern human out-of-Africa migration time (90 kya; 95% HPD, 60 to 120 kya) to calibrate the times for the most recent common ancestor (MRCA) of HPV58 variants (Fig. 4, arrows). When time points were introduced into the HPV58 variant tree, similar divergence times were estimated, with a mean substitution rate of HPV58 variants ranging between 1.72 × 10^−8^ and 1.91 × 10^−8^ substitutions/site/year. For the final plot, the HHS scenario with a combination of a relaxed log-normal molecular clock and coalescent Bayesian skyline models showed the strongest support (Akaike's information criterion for MCMC samples [AICM] of 32,648) for the time inference of HPV58 variants (Fig. 4). The initial divergence of HPV58 variants was estimated to be approximately 479 kya (95% HPD, 391 to 570 kya), largely predating the out-of-Africa migration of modern humans (60 to 120 kya).

**TABLE 5:** Divergence time estimations for HPV58 variant lineages^*a*^

Calibration	Rate (10^−8^)	Clock model	Tree prior	AICM	Log marginal likelihood	Estimated rate (10^−8^)		MRCA (kya)													
						Mean	95% HPD interval	Node 0		Node 1		Node 2		Node 3		Node 4		Node 5		Node 6	
								Mean	95% HPD interval	Mean	95% HPD interval	Mean	95% HPD interval	Mean	95% HPD interval	Mean	95% HPD interval	Mean	95% HPD interval	Mean	95% HPD interval
No	Feline PV	Relaxed	Bayesian	32,649	−16,239.82	1.84	1.61, 2.08	451.6	306.0, 619.6	191.9	125.0, 266.5	149.8	98.1, 206.8	356.6	246.5, 477.2	281.6	193.7, 376.6	220.1	147.1, 296.8	128.3	82.7, 178.7
No	1.95 (1.32–2.47)	Relaxed	Yule	+7	−16,241.02	1.95	1.73, 2.17	318.8	241.4, 407.8	179.1	125.9, 235.8	146.9	105.8, 192.3	270.8	202.3, 343.1	223.2	164.1, 283.3	182.6	134.7, 236.2	120.8	86.3, 160.8
No		Relaxed	Constant	+10	−16,239.91	1.67	1.40, 1.94	545.9	352.7, 778.2	232.7	143.6, 341.5	182.0	112.9, 258.5	433.7	293.1, 608.5	343.4	230.9, 474.1	269.3	180.3, 377.8	158.7	97.5, 223.1
No		Strict	Bayesian	+33	−16,287.82	1.89	1.33, 2.41	397.4	272.0, 545.9	187.4	123.8, 265.7	156.3	102.6, 221.0	327.6	223.6, 455.3	274.2	188.4, 379.3	230.3	157.6, 322.2	133.7	86.1, 189.4
No		Strict	Yule	+37	−16,288.68	1.96	1.40, 2.47	357.6	252.5, 496.5	178.5	120.8, 251.8	151.3	103.3, 215.8	298.1	209.3, 415.9	252.3	176.0, 352.4	213.9	149.9, 300.7	129.1	86.6, 184.2
No		Strict	Constant	+41	−16,289.52	1.90	1.33, 2.42	409.5	286.8, 570.3	197.4	128.4, 277.6	166.1	110.4, 237.8	339.8	228.3, 468.4	285.8	195.1, 397.5	240.9	163.8, 337.1	142.6	92.4, 201.2
**2 Cali.**^*b*^	**HPV16**	**Relaxed**	**Bayesian**	**32,648**	**−16,239.97**	**1.82**	**1.50, 2.13**	**478.6**	**391.0, 569.6**	**197.6**	**122.2, 283.3**	**152.8**	**100.3, 219.8**	**358.1**	**244.8, 472.7**	**273.3**	**183.1, 367.0**	**206.9**	**138.7, 285.6**	**103.0**	**79.2, 125.2**
2 Cali.	1.84 (1.43−2.21)	Relaxed	Yule	+7	−16,241.14	1.72	1.41, 2.08	425.4	339.6, 516.6	213.4	142.7, 294.7	171.3	115.1, 237.1	320.2	228.0, 417.0	252.6	177.5, 331.1	198.1	141.5, 265.8	108.7	85.7, 132.0
2 Cali.		Relaxed	Constant	+9	−16,240.87	1.81	1.52, 2.08	490.8	402.1, 580.2	224.6	143.5, 329.3	176.5	111.8, 255.6	385.2	270.2, 499.6	298.0	205.1, 402.9	226.4	151.0, 314.8	106.8	84.1, 130.8
2 Cali.		Strict	Yule	+40	−16,288.79	1.83	1.50, 2.16	348.9	301.8, 398.8	174.5	142.8, 208.9	147.9	119.3, 176.8	290.3	246.6, 334.2	245.8	209.6, 283.6	208.3	175.0, 241.1	125.7	102.0, 151.8
2 Cali.		Strict	Bayesian	+45	−16,289.89	1.85	1.54, 2.18	418.7	341.5, 494.1	192.4	143.4, 241.6	159.2	116.8, 200.8	327.9	257.8, 397.8	269.9	214.8, 329.9	222.6	174.2, 272.1	114.5	93.8, 135.3
2 Cali.		Strict	Constant	+46	−16,291.17	1.91	1.62, 2.21	417.2	343.8, 491.1	197.3	150.9, 248.6	165.0	125.8, 208.9	328.6	265.3, 400.5	271.5	217.7, 328.8	224.5	177.6, 273.1	117.0	96.0, 138.2

a Node numbers match the notation in Fig. 4.

b Two time points were introduced in the HPV58 variant tree to calibrate the time estimate. Boldface indicates the time estimate with the “best” models.

The potential ancestral codon mutations were predicted by using a maximum likelihood regression model (see Table S3 in the supplemental material). Interestingly, some sites associated with the E7 T20I/G63S variant that carries higher carcinogenicity had mutated earlier than the time of the divergence of the A3 sublineage. For example, E7 G760A within B1 variants (branch 3 [Br3] in Fig. 4 and Table S3 in the supplemental material) and E1 A1965T within the MRCA of A variants (Br10) may represent ancient adaptation or fitness in archaic hominins.

## DISCUSSION

HPV58 is one of the oncogenic types attributing to a high proportion of cervical cancers in East Asia (8). HPV variants have been reported to be different in persistence, viral load, and carcinogenicity (11, 25–27), indicating that each HPV lineage has a different evolutionary history in their host population. In this study, we applied multiple phylogenetic algorithms and used a large data set of complete genome and partial gene sequences to investigate the origin, dispersal, and diversity of HPV58 variants. We observed a predominant dispersal of HPV58 A variants worldwide and A1/A3 variants in Asia; most importantly, the estimated divergence times of HPV58 variants largely predated the recent out-of-Africa migration of modern human populations. These findings confirm a hominin host switch scenario previously reported for HPV16 showing that the ancient hominin-virus codivergence and recent host switch events shaped the radiation that we observe in the phylogenetic tree of extant HPV variants (24). The majority of HPV variants currently predominant in Eurasian populations could be the descendants of viral transmission from Neanderthals/Denisovans to modern human populations through interbreeding and gene flow.

**FIG 5:**
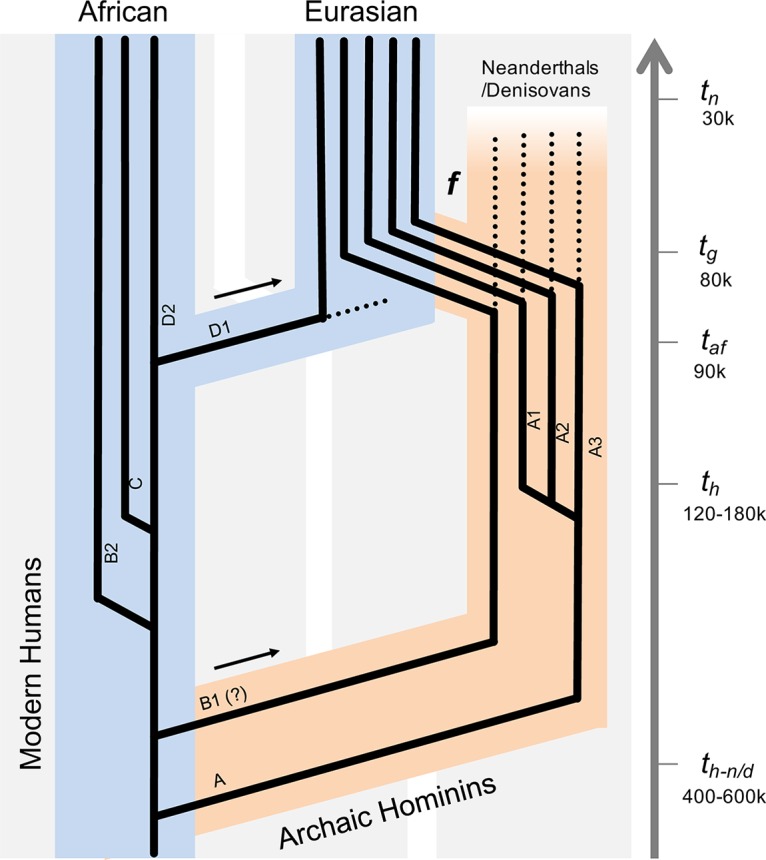
Schematic illustration of HPV58 codivergence with archaic hominins. The model is based on HPV58 variant divergence time estimations, phylogenetic topology, and geographic distributions that superimpose ancestral viral transmission between Neanderthals/Denisovans and modern human populations. *t_h-n/d_* denotes the splitting time between Neanderthals/Denisovans and modern humans, *t_h_* represents the speciation of modern humans, *t_af_* indicates the era of population expansion of modern humans walking out of Africa, *t_g_* indicates the time of gene flow (*f*) that may have occurred between modern humans and Neanderthals/Denisovans, and *t_n_* estimates the extinction of Neanderthals/Denisovans. The arrows indicate the out-of-Africa migration events of archaic and modern human populations. The broken lines indicate the potential extinction of viral variants. Branch lengths and widths are not drawn to scale.

The divergence time estimation and the phylogenetic separation between HPV58 A and non-A variants mirror the host split between archaic hominins and modern human ancestors, indicating that ancestral HPV58 lineages may have already existed before the emergence of modern humans, which rejects the initial assumption of codivergence between HPV and H. sapiens migration (22). Interestingly, the initial divergence of ancestral HPV58 lineages estimated here, similar to the split between HPV16 lineages A and BCD (461 kya; 95% HPD, 365 to 561 kya) (24), is compatible with the speciation between Neanderthals/Denisovans and modern H. sapiens ancestors (Fig. 5). When Neanderthal/Denisovan ancestors diverged and expanded out of Africa, they may have carried ancestral HPV variants (e.g., HPV16A or HPV58A). The morphological features typical of Neanderthals first appeared in the European fossil record about 400,000 to 600,000 years ago. Progressively more distinctive hominin forms subsequently evolved (e.g., splitting of Neanderthals and Denisovans) until their extinction around 30,000 years ago (28–30). During the late part of their history, Neanderthals lived in Europe and Western Asia and presumably came into contact with anatomically modern humans in the Middle East from at least 80,000 years ago. Interbreeding between Neanderthals/Denisovans and modern humans is probably largely responsible for the sexual transmission of viruses through host gene flow. This inference is made based on the supposed 2 to 4% of nuclear DNA in Eurasians that can be traced to Neanderthals (29, 31). This scenario can also be confirmed in part by the very low prevalences of certain HPV58 A variants (particular the A1 and A3 sublineages) in current African populations, strongly indicating that these variants were of Neanderthal origin. Viral transmission from modern humans to Neanderthals/Denisovans was possible based on evidence of ancient gene flow from early modern humans to Eastern Neanderthals (30). However, papillomavirus usually establishes infection at the basal layer of epithelium cells, making it impossible to detect viruses from fossil bones of archaic hominins.

The real divergence of HPV58 variants is likely more complex. In parallel with the ancient out-of-Africa expansion of Neanderthals/Denisovans, the viruses remaining in Africa codiversified with subsequent host speciation. Some viral variants (e.g., the D1 sublineage) may have dispersed outside Africa following the recent out-of-Africa migration of modern humans, while some expanded in one or more isolated hominin populations in Africa or became extinct. The MRCA for HPV58 non-A variants (358 kya; 95% HPD, 245 to 473 kya) seems older than the one for A variants (198 kya; 95% HPD, 122 to 283 kya) (Fig. 4 and Table 5), consistent with the low genomic diversity of A variants, probably because of a population bottleneck where only a proportion of viruses in Neanderthals/Denisovans was able to be horizontally transferred to modern humans. In contrast, HPV58 non-A variants are more diversified, matching the observation that African populations were the most diverse populations genetically (32). This may also support the notion that both modern humans and HPV58 non-A variants arose in Africa. Besides, interbreeding between archaic hominins was multiregional, occurring at different times and places (29). For example, a subset of modern human ancestors who carried some Neanderthal DNA may have headed east and interbred with Denisovans in Oceania (e.g., Australia, Melanesia, and the Philippines) (33). Although the contribution of Denisovans to modern humans was quantitatively small, gene flow from ancient Oceanians (after they mixed with Denisovans) to mainland Asian ancestors may have accumulated; as a result, certain viral variants (e.g., A1 and A3 sublineages) became more predominant in East Asia. While we were able to analyze the largest available worldwide collection of HPV58 isolates, the number of samples from certain areas is small, precluding a determination of the accurate geographic distribution of viral variants in different geographic areas. For example, the history of B1 variants is still elusive. The B1 sublineage is closer to B2, but they cannot be classified in the same monophyletic clade (Fig. 1); independent evolutionary histories encompassed by each of them may explain their differences in dispersal and frequency in present-day populations.

Similar to HPV16 and HPV18 genomes (34, 35), the majority of HPV58 variant genes are under purifying selection, with average *dN*/*dS* ratios of <1. The low substitution rate limits the actual number of evolutionary events and maintains the core functions of HPV-encoded proteins in a neutral fashion. In contrast, several codon sites under positive selection may affect viral phenotypes involved in facilitating host immune evasion, maintaining asymptomatic infection, or enhancing viral persistence and replication. For example, E7 aa 63 is under positive selection, with 3 different amino acid changes at the same codon: G63S is shared by the A3 and B1 sublineages, G63D is conserved in the A2 and D2 sublineage, and G63H is unique to the D1 sublineage. Such genetic changes possibly display differential viral loads and infection persistence that are directly involved in enhanced adaptive introgression in host alleles. Alternatively, the genetic variations that occur in HPV58 may be induced partly by the host innate immune system, such as the antiviral activity of hA3 cytidine deaminases (36). It has been reported that APOBEC3-mediated cytidine deaminase activity could target HPV16 genes and induce viral mutations (37). These induced mutations, if not lethal, may also be responsible for the long-term accumulation of genomic changes that affect the success of niche adaptation or function fitness contributing to HPV-associated cancer (38). Nevertheless, functional investigations will be warranted to clarify whether these and other genotype changes affect any phenotypes in present-day HPV variants.

To date, this is the most comprehensive study on the evolutionary history of HPV58 variants. One interpretation of the data in this work implies ancient hominin-virus codivergence between human papillomaviruses and hosts, while certain HPV variants, particularly the isolates predominant in present-day Eurasia, could be the descendants of sexual transmission from Neanderthals/Denisovans to modern human populations. The implementation of new technology such as next-generation sequencing makes sequencing of large amounts of complete HPV genomes more feasible and economical (11, 39, 40). A high-throughput, ultradeep-coverage method permits a more detailed examination of genotype-phenotype relationships between viral genomics and carcinogenesis. It will also help to establish local adaptation between virus and host genomes to explain the differential dispersals and cancer risks of HPV variants in different ethnic populations.

## MATERIALS AND METHODS

### Isolates and complete genome sequencing.

In our previous study, 445 HPV58 isolates collected from 15 countries/cities across four continents had been sequenced for E6-E7-E2-E5-L1-LCR (7, 13). In the present study, we identified 44 isolates that carry unique variation sites or patterns for complete genome sequencing. Type-specific primers were designed to amplify the complete genome by using nested overlapping PCR as previously reported (10). The collection of samples for sequencing analysis had been approved during the course of previous studies.

### Phylogenetic analysis and tree construction.

Ninety complete HPV58 genomes, including 44 new sequences from the present study, 26 sequences previously reported by our group (10), and 20 sequences available in the NCBI/GenBank database, were used for evolutionary analysis (see Table S1 in the supplemental material) (40–46). ML trees were constructed by using RAxML MPI v8.2.9 (47) and PhyML MPI v3.0 (48) with optimized parameters based on complete genome nucleotide sequences aligned by MAFFT v7.221 (49). Data were bootstrap resampled 1,000 times in RAxML and PhyML. MrBayes v3.2.6 (50) with 10,000,000 cycles for the MCMC algorithm was used to generate Bayesian trees. A 10% discarded burn-in was set to eliminate iterations at the beginning of the MCMC run. For Bayesian tree construction, a general time-reversible (GTR) model identified by ModelTest v3.7 (51) was set for among-site rate variation, allowing substitution rates of aligned sequences to be different. The CIPRES Science Gateway (52) was accessed to facilitate RAxML and MrBayes high-performance computing. To detect phylogenetic congruence among genes of HPV58 variants, separated ML trees inferred from concatenated nucleotide sequences of early genes (E6, E7, E1, and E2 ORFs) and late genes (L2 and L1 ORFs) were constructed using RAxML. SimPlot (53) and GARD within the HyPhy distribution (54) were used to detect potential recombination events across six genes. Mutations potentially due to the antiviral activity of hA3 cytosine deaminases (T**C**R→T**K**R or Y**T**A→Y**M**A; boldface indicates the mutation in the trinucleotide motif; M = A or C; K = G or T; R = A or G; Y = C or T) were excluded from tree comparisons and recombination tests (38).

### Sequence diversity and genomic signatures.

SNPs and amino acid changes were determined by using scripts developed in-house with R v3.3.2 (55). The rarefaction curves of SNPs were generated by EstimateS v9.1.0 (56). Inter- and intralineage nucleotide sequence differences were calculated by using the *p*-distance method in MEGA6 (57). A Wilcoxon-Mann-Whitney U test was used to determine the significance of pairwise differences between the defined groups. Genome-wide sequence signature analysis using MI computation was applied to examine the strength of the association between two variations (58). The pairwise MI indexes were calculated by using the mutinformation function in the R package “infotheo.” A higher MI value indicates greater agreement between two compared sites.

### Detection of positive selection.

The maximum likelihood regression models of codon substitution (ω = *dN*/*dS*) were applied to identify whether an HPV58 gene(s) was under positive selection (59, 60). These models view the codon as the fundamental unit of evolutionary change and take into account genealogic history when calculating scores. Log-likelihood scores evaluate the quality of the fit of the input data to the conditions of the model. Six models, including M0 (one ratio), M1 (neutral), M2 (selection), M3 (discrete), M7 (beta), and M8 (beta and ω), used for the ω distribution of distinct ORFs (E6, E7, E1, E2, E4, E5, L2, or L1), were implemented in the CODEML program in the PAML v4.8a package (60), with a guided RAxML tree inferred from the global complete genome alignment. The codon sequences of each ORF were aligned based on the amino acid alignment by MUSCLE (61). The ratio of nonsynonymous/synonymous substitution rates is an indicator of natural selection, with a ω value of 1 representing neutral variation, a ω value of <1 representing purifying selection, and a ω value of >1 representing diversifying positive selection. Three LRTs were performed to assess the influence of positive selection on a particular coding region, which compared M1 with M2, M0 with M3, and M7 with M8. When alternative models (M2, M3, and M8) suggest the presence of sites with a ω value of >1, results of all three tests taken together are considered evidence of positive selection (34, 35). Amino acid sites in a protein are expected to be under different selective pressures and have different underlying ω ratios.

A FUBAR algorithm within the HyPhy distribution (62) was used to verify the sites under positive selection observed by CODEML. This hierarchical Bayesian MCMC method ensures robustness against model misspecification by averaging over a large number of predefined site classes. A codon site with a ω value of >1 was considered to be under positive selection when the posterior probabilities determined by CODEML and FUBAR were ≥0.950 and 0.9000, respectively.

### Geographic distribution of HPV58 variants.

Information on the geographic sources of isolates was obtained from the corresponding publications (7, 42, 63–66). Tree topologies of partial sequences spanning variable genes/regions of 747 HPV58 isolates obtained worldwide (Table 4) were constructed using pplacer v1.1.alpha17 (67) by placing short sequences on a reference tree to maximize phylogenetic likelihood according to a complete genome alignment. The reference RAxML tree was based on 90 complete genomes that we used for evolutionary analysis. A cutoff value of a maximum likelihood of ≥0.8 was set as a confident assignment of HPV58 isolates into each (sub)lineage. The abundances of each lineage from the same country/city were combined and normalized as a percentage. We used a weighted UniFrac method in the R package “GUniFrac” (68) to calculate the pairwise distances between geographic locations, based upon which a PCA was performed to visualize the clustering of countries/cities by using the betadisper function in the R package “vegan.” Four geographic groups, Africa, America, Asia, and Europe, were summarized for the distribution of HPV58 variant lineages; for each lineage, its frequency in each geographic group was calculated based on the summary of individual percent abundances divided by the summary of the total percent abundance.

### Divergence time estimation.

We used a Bayesian MCMC method implemented in BEAST2 v.2.4.5 (69) to estimate the divergence times of HPV58 variants. Three tree priors were estimated by using the (i) coalescent constant population, (ii) Yule model, and (iii) coalescent Bayesian skyline demographic model, with assumptions that the papillomavirus genome has a strict mutation rate or that there is an uncorrelated log-normal distribution (UCLD) molecular clock model of rate variation among branches (Table 5). We chose the GTR sequence revolution model with gamma-distributed rate heterogeneity among sites and a proportion of invariant sites (GTR+G+I) determined by the best-fit model approach of Modeltest v3.7 (51). The complete genome alignment and a previously reported PV evolutionary rate of 1.95 × 10^−8^ substitutions per site per year (95% confidence interval [CI], 1.32 × 10^−8^ to 2.47 × 10^−8^ substitutions per site per year) were used (17).

To validate the accuracy of the time estimation, an HHS model assuming that there was ancestral viral transmission between archaic and modern human populations (24) was applied by setting two evolutionary time points to calibrate the HPV58 variant phylogenetic tree: (i) the archaic divergence of modern humans and Neanderthals/Denisovans around 500 kya (95% CI, 400 to 600 kya) (70), matching the split between HPV58 A and non-A variants, and (ii) the modern human out-of-Africa migration at 90 kya (95% CI, 60 to 120 kya) (71, 72), locating the era when HPV58 D1 and D2 variants diverged from their MRCA. An HPV16 variant substitution rate was used for validation as a uniform prior, 1.84 × 10^−8^ substitutions per site per year (95% CI, 1.43 × 10^−8^ to 2.21 × 10^−8^ substitutions per site per year) (24), with combinations of three tree priors and two clock models as described above.

The MCMC analysis was run for 100,000,000 steps, with subsampling every 10,000 generations. A discarded burn-in of the first 10% of steps was set to refine trees and log files for further analysis. The best model estimates were selected by using a posterior simulation-based analogue of AICM (73), as implemented in Tracer v.1.6. Lower AICM values indicate a better model fit. A consensus tree was inferred by using TreeAnnotater v.2.4.5 and visualized by using in-house-developed scripts in R.

### Accession number(s).

Accession numbers for the sequences determined in this study are available in GenBank under accession numbers KY225918 to KY225932, KY225934, KY225936 to KY225939, KY225941 to KY225959, KY225961, KY225963, KY225964, KY225966, and KY225967 (see Table S1 in the supplemental material).

## Supplementary Material

Supplemental material
