# Supplementary material for: Ancient Evolution and Dispersion of Human Papillomavirus 58 Variants
Source: J Virol. 2017 Oct 13;91(21):e01285-17. doi: 10.1128/JVI.01285-17 (PMC5640864; doi:10.1128/JVI.01285-17)
Supplement: Supplemental material [file supp_91_21_e01285-17__index.html]

Supplemental material 

# Ancient Evolution and Dispersion of Human Papillomavirus 58 Variants

## Supplemental material

- Supplemental file 1 -

  Table S1 (Sample list of HPV58 complete genomes included in this study.)

  Table S2 (Sequence variations of the HPV58 complete genomes.)

  Table S3 (Ancestral codon mutation of each lineage and sublineage using a maximum likelihood regression model.)

  PDF, 779K
