# Supplementary material for: Ancient Evolution and Dispersion of Human Papillomavirus 58 Variants
Source: J Virol. 2017 Oct 13;91(21):e01285-17. doi: 10.1128/JVI.01285-17 (PMC5640864; doi:10.1128/JVI.01285-17)
Supplement: Supplemental material [file JVI.01285-17_zjv999182996s1.pdf]

Table S1. Sample list of HPV58 complete genome included in this study.

Sample ID	NCBI Accession	Lineage	Sublineage	Length (bp)	Country	Histology/Cytology	Reference
PPH58	D90400	HPV58-A	HPV58-A1	7824	NA	NA	(1)
LZCC86	EU918765	HPV58-A	HPV58-A1	7824	China	NA	(2)
SC100	FJ385261	HPV58-A	HPV58-A1	7824	China	NA	(3)
SC101	FJ385262	HPV58-A	HPV58-A1	7824	China	NA	(3)
SC147	FJ385263	HPV58-A	HPV58-A1	7824	China	NA	(3)
SC144	FJ385264	HPV58-A	HPV58-A1	7824	China	NA	(3)
SC165	FJ385265	HPV58-A	HPV58-A1	7824	China	NA	(3)
SC174	FJ385266	HPV58-A	HPV58-A1	7824	China	NA	(3)
SC185	FJ385267	HPV58-A	HPV58-A1	7836	China	NA	(3)
SC78	FJ385268	HPV58-A	HPV58-A1	7824	China	NA	(3)
TJ18	GQ472850	HPV58-A	HPV58-A2	7824	China	NA	(4)
QV15606	HQ537752	HPV58-A	HPV58-A2	7824	Costa Rica	NA	(5)
RW791	HQ537753	HPV58-A	HPV58-A2	7824	Rwanda	NA	(5)
QV03554	HQ537754	HPV58-A	HPV58-A2	7824	Costa Rica	NA	(5)
QV00861	HQ537755	HPV58-A	HPV58-A2	7824	Costa Rica	NA	(5)
QV15563	HQ537756	HPV58-A	HPV58-A3	7836	Costa Rica	NA	(5)
QV32351	HQ537757	HPV58-A	HPV58-A3	7836	Costa Rica	NA	(5)
QV00961	HQ537758	HPV58-A	HPV58-A3	7836	Costa Rica	NA	(5)
AS405	HQ537759	HPV58-A	HPV58-A3	7836	Taiwan	NA	(5)
AS347	HQ537760	HPV58-A	HPV58-A3	7836	Taiwan	NA	(5)
BF077	HQ537761	HPV58-B	HPV58-B1	7823	Burkina Faso	NA	(5)
BF134	HQ537762	HPV58-B	HPV58-B1	7823	Burkina Faso	NA	(5)
Z023	HQ537763	HPV58-B	HPV58-B1	7823	Zambia	NA	(5)
RW937	HQ537764	HPV58-B	HPV58-B2	7825	Rwanda	NA	(5)
RW754	HQ537765	HPV58-B	HPV58-B2	7825	Rwanda	NA	(5)
QV03858	HQ537766	HPV58-D	HPV58-D1	7814	Costa Rica	NA	(5)
QV04732	HQ537767	HPV58-D	HPV58-D1	7814	Costa Rica	NA	(5)
QV03841	HQ537768	HPV58-D	HPV58-D1	7814	Costa Rica	NA	(5)
RW841	HQ537769	HPV58-D	HPV58-D2	7817	Rwanda	NA	(5)
RW697	HQ537770	HPV58-D	HPV58-D2	7817	Rwanda	NA	(5)
RW63	HQ537771	HPV58-D	HPV58-D2	7817	Rwanda	NA	(5)

Table S1. (cont.)

Sample ID	NCBI Accession	Lineage	Sublineage	Length (bp)	Country	Histology/Cytology	Reference
QV34982	HQ537772	HPV58-C	HPV58-C	7820	Costa Rica	NA	(5)
QV03666	HQ537773	HPV58-C	HPV58-C	7820	Costa Rica	NA	(5)
QV13816	HQ537774	HPV58-C	HPV58-C	7820	Costa Rica	NA	(5)
RW792	HQ537775	HPV58-C	HPV58-C	7820	Rwanda	NA	(5)
RW644	HQ537776	HPV58-C	HPV58-C	7820	Rwanda	NA	(5)
Z094	HQ537777	HPV58-C	HPV58-C	7820	Zambia	NA	(5)
JP0221	AB819275	HPV58-A	HPV58-A2	7824	Japan	NA	(6)
JP0302	AB819276	HPV58-A	HPV58-A2	7824	Japan	NA	(6)
JP0891	AB819277	HPV58-A	HPV58-A2	7824	Japan	NA	(6)
JP1352	AB819278	HPV58-A	HPV58-A2	7824	Japan	NA	(6)
JP1870	AB819279	HPV58-A	HPV58-A3	7836	Japan	NA	(6)
CNZJ1	KC860269	HPV58-A	HPV58-A2	7824	China	NA	(7)
CNZJ3	KC860270	HPV58-A	HPV58-A1	7824	China	NA	(7)
CNZJ2	KC860271	HPV58-A	HPV58-A2	7824	China	NA	(7)
36A	KU298920	HPV58-A	HPV58-A2	7781	Brazil	NA	(8)
TW00060	KY225918	HPV58-A	HPV58-A1	7824	Taiwan	SCC	this work
QE00190	KY225919	HPV58-A	HPV58-A1	7824	Hong Kong	CIN1	this work
ZWE058771	KY225920	HPV58-A	HPV58-A1	7824	Zimbabwe	NA	this work
ZWE047402	KY225921	HPV58-A	HPV58-A1	7824	Zimbabwe	NA	this work
KORK01712	KY225922	HPV58-A	HPV58-A2	7824	South Korea	Normal	this work
KORK03505	KY225923	HPV58-A	HPV58-A2	7824	South Korea	Normal	this work
ZWE052265	KY225924	HPV58-A	HPV58-A2	7827	Zimbabwe	NA	this work
ZWE051406	KY225925	HPV58-A	HPV58-A2	7827	Zimbabwe	NA	this work
KORK00020	KY225926	HPV58-A	HPV58-A2	7824	South Korea	LSIL	this work
KORK00038	KY225927	HPV58-A	HPV58-A2	7824	South Korea	Normal	this work
KORK00643	KY225928	HPV58-A	HPV58-A2	7824	South Korea	ASCUS	this work
KORK00053	KY225929	HPV58-A	HPV58-A2	7824	South Korea	ASCUS	this work
KORK02118	KY225930	HPV58-A	HPV58-A2	7824	South Korea	LSIL	this work
KORK00421	KY225931	HPV58-A	HPV58-A2	7824	South Korea	SCC	this work
KORK00025	KY225932	HPV58-A	HPV58-A2	7824	South Korea	ASCUS	this work
QE01538	KY225934	HPV58-A	HPV58-A2	7824	Hong Kong	CIN1	this work

Table S1. (cont.)

Sample ID	NCBI Accession	Lineage	Sublineage	Length (bp)	Country	Histology/Cytology	Reference
KORK02546	KY225936	HPV58-A	HPV58-A3	7836	South Korea	HSIL	this work
KORK00008	KY225937	HPV58-A	HPV58-A3	7836	South Korea	LSIL	this work
KORK00613	KY225938	HPV58-A	HPV58-A3	7836	South Korea	HSIL	this work
KORK00191	KY225939	HPV58-A	HPV58-A3	7836	South Korea	HSIL	this work
KORK01374	KY225941	HPV58-A	HPV58-A3	7836	South Korea	HSIL	this work
KORK00034	KY225942	HPV58-A	HPV58-A3	7836	South Korea	LSIL	this work
KORK03823	KY225943	HPV58-A	HPV58-A3	7836	South Korea	Normal	this work
KORK00099	KY225944	HPV58-A	HPV58-A3	7836	South Korea	HSIL	this work
KORK02277	KY225945	HPV58-A	HPV58-A3	7836	South Korea	HSIL	this work
KORK00064	KY225946	HPV58-A	HPV58-A3	7836	South Korea	HSIL	this work
KORK00043	KY225947	HPV58-A	HPV58-A3	7836	South Korea	HSIL	this work
QE00150	KY225948	HPV58-A	HPV58-A3	7836	Hong Kong	ICC	this work
THA00468	KY225949	HPV58-A	HPV58-A3	7836	Thailand	SCC	this work
KORK02550	KY225950	HPV58-A	HPV58-A3	7836	South Korea	Normal	this work
KORK00011	KY225951	HPV58-A	HPV58-A3	7836	South Korea	LSIL	this work
KORK03762	KY225952	HPV58-A	HPV58-A3	7836	South Korea	LSIL	this work
JPNJ00739	KY225953	HPV58-A	HPV58-A3	7836	Japan	Normal	this work
QE00470	KY225954	HPV58-A	HPV58-A3	7836	Hong Kong	CIN2	this work
QE01132	KY225955	HPV58-A	HPV58-A3	7836	Hong Kong	CIN3	this work
ZWE051089	KY225956	HPV58-B	HPV58-B2	7825	Zimbabwe	NA	this work
ZWE043998	KY225957	HPV58-B	HPV58-B2	7825	Zimbabwe	NA	this work
ZWE062097	KY225958	HPV58-C	HPV58-C	7820	Zimbabwe	NA	this work
ZWE050364	KY225959	HPV58-C	HPV58-C	7820	Zimbabwe	NA	this work
ZWE054176	KY225961	HPV58-C	HPV58-C	7863	Zimbabwe	NA	this work
ZWE044033	KY225963	HPV58-C	HPV58-C	7820	Zimbabwe	NA	this work
ARGP00138	KY225964	HPV58-D	HPV58-D1	7817	Argentina	Normal	this work
ZWE064436	KY225966	HPV58-D	HPV58-D2	7817	Zimbabwe	NA	this work
ZWE051402	KY225967	HPV58-D	HPV58-D2	7808	Zimbabwe	NA	this work

Table S2. Sequence variations of the HPV58 complete genomes. The stars indicate changes consistent with an APOBEC3-induced change.

		E6	E7	E1	
APOBEC3-induced change		. . . * * *	. . . . *	[illegible]	[illegible]
Nucleotide site (prototype)		136 143 161 187 193 203 245 246 260 307 367 374 388 392 398 400	587 591 614 632 694 726 744 756 760 761 763 775 778 790 793 798 801 803 840 852	[illegible]	[illegible]
PPH58|D90400	A1	A T G C G G G T A C C G A T A T	A G A C G T T T G G A T T A A C C T C T	[illegible]	[illegible]
SC165|FJ385265	A1	. . . . C . . .	. . . .	[illegible]	[illegible]
SC147|FJ385263	A1	. . . . C . . .	. . . .	[illegible]	[illegible]
SC100|FJ385261	A1	. . . .	. . . .	[illegible]	[illegible]
LZCC86|EU918765	A1	. . . .	. . . .	[illegible]	[illegible]
SC144|FJ385264	A1	. . . .	. . . .	[illegible]	[illegible]
SC101|FJ385262	A1	. . . . C . . .	. . . . . . G . . . . C . .	[illegible]	[illegible]
SC174|FJ385266	A1	. . . . C . . .	. A . . . . G . . . . C . .	[illegible]	[illegible]
SC185|FJ385267	A1	. . . . A C . . .	. . . . . . G . . . . C . .	[illegible]	[illegible]
TW60|NA	A1	. . . . C . . .	. . . . . . G . . . . C . .	[illegible]	[illegible]
CNZJ3|KC860270	A1	. . . . C . . .	. . . . . . G . . . . C . .	[illegible]	[illegible]
QE190|NA	A1	. . . . C . . .	. . . . . . G . . . . C . .	[illegible]	[illegible]
SC78|FJ385268	A1	. . . . C . . .	. . . . . . G . . . . C . .	[illegible]	[illegible]
058771|NA	A1	. . . .	. . . . . C	[illegible]	[illegible]
047402|NA	A1	. . . .	. . . . . C	[illegible]	[illegible]
QV00861|HQ537755	A2	. . . T . . . . . T	. . . . . . G	[illegible]	[illegible]
K1712|NA	A2	. . . . T	. . . . A . G . . A	[illegible]	[illegible]
K3505|NA	A2	. . . . T	. . . . A . G . . A	[illegible]	[illegible]
JP0221|AB819275	A2	. . . . T	. . . . A . G . . A	[illegible]	[illegible]
QV03554|HQ537754	A2	. . . . T	. . G . A . G . . A . . C	[illegible]	[illegible]
JP1352|AB819278	A2	. . . . T	. . . . A . G . . A	[illegible]	[illegible]
052265|NA	A2	. . . . T	. . . . A . G . . A	[illegible]	[illegible]
051406|NA	A2	. . . . T	. . . . A . G . . A	[illegible]	[illegible]
RW791|HQ537753	A2	. . . . T	. . . . A . G . . A	[illegible]	[illegible]
CNZJ1|KC860269	A2	. . . . T	. . . . A . G . . A	[illegible]	[illegible]
K20|NA	A2	. . . . T	. . . . A . G . . A	[illegible]	[illegible]
K38|NA	A2	. . . . T	. . . . A . G . . A	[illegible]	[illegible]
K643|NA	A2	. . . . T	. . . . A . G . . A	[illegible]	[illegible]
JP0891|AB819277	A2	. . . . T	. . . . A . G . . A	[illegible]	[illegible]
CNZJ2|KC860271	A2	. . . . T	. . . . A . G . . A	[illegible]	[illegible]
K53|NA	A2	. . . . T	. . . . A . G . . A	[illegible]	[illegible]
K2118|NA	A2	. . . . T	. . . . A . G . . A	[illegible]	[illegible]
K421|NA	A2	. . . . T	. . . . A . G C . A	[illegible]	[illegible]
JP0302|AB819276	A2	. . . . T	. . . . A . G C . A	[illegible]	[illegible]
K25|NA	A2	. . . . T	. . . . A . G C . A	[illegible]	[illegible]
QV15606|HQ537752	A2	. . . . T	. . . . A . G . . A	[illegible]	[illegible]
36A|KU298920	A2	. . . . T	. . . . A . G . . A	[illegible]	[illegible]
TJ18|GQ472850	A2	. . . . T	. . . . A . G . . A G . . G	[illegible]	[illegible]
QE1538|NA	A2	. . . . T	. . . . A . G . . A	[illegible]	[illegible]
QV32351|HQ537757	A3	. . . . T	. . . T . . G . A	[illegible]	[illegible]
QV15563|HQ537756	A3	. . . . T	. . . T . . G . A	[illegible]	[illegible]
AS347|HQ537760	A3	. . . . T	. . . T . . G . A	[illegible]	[illegible]
K2546|NA	A3	. . . . T	. . . T . . G . A	[illegible]	[illegible]
K8|NA	A3	. . . . T	. . . T . . G . A	[illegible]	[illegible]
K613|NA	A3	. . . . T	. . . T . . G . A	[illegible]	[illegible]
K191|NA	A3	. . . . T	. . . T . . G . A	[illegible]	[illegible]
K1374|NA	A3	. . . . T	. . . T . . G . A	[illegible]	[illegible]
JP1870|AB819279	A3	. . . . T	. . . T . . G . A	[illegible]	[illegible]
K34|NA	A3	. . . . T	. . . T . . G . A	[illegible]	[illegible]
K3823|NA	A3	. . . . T	. . . T . . G . A	[illegible]	[illegible]
K99|NA	A3	. . . . T	. . . T . . G . A	[illegible]	[illegible]
K2277|NA	A3	. . . . T	. . . T . . G . A	[illegible]	[illegible]
K64|NA	A3	. . . . T	. . . T . . G . A	[illegible]	[illegible]
K43|NA	A3	. . . . T	. . . T . . G . A	[illegible]	[illegible]
AS405|HQ537759	A3	. . . . T	. . . T . . G . A	[illegible]	[illegible]
QE150|NA	A3	. . . . T	. . . T . . G . A	[illegible]	[illegible]
THA468|NA	A3	. . . . T	. . . T . . G . A	[illegible]	[illegible]
K2550|NA	A3	. . . . T	. . . T . . G . A	[illegible]	[illegible]
K11|NA	A3	. . . . T	. . . T . . G . A	[illegible]	[illegible]
K3762|NA	A3	. . . . T	. . . T . . G . A	[illegible]	[illegible]
J739|NA	A3	. . . . T	. A . T . . G . A	[illegible]	[illegible]
QE470|NA	A3	. . . . T	. . . T . . G . A	[illegible]	[illegible]
QE1132|NA	A3	. . . . T	. . . T . . G . A	[illegible]	[illegible]
QV00961|HQ537758	A3	G . . . . T	. . . T . . G . A	[illegible]	[illegible]
Z023|HQ537763	B1	. . . . . C . . . T A . C . . .	. . . . . . G . A . . . . . G T A . . .	[illegible]	[illegible]
BF134|HQ537762	B1	. . . . . C . . . T A	. . . . . . G . A . . . . . G T A . . .	[illegible]	[illegible]
BF077|HQ537761	B1	. . . T . C . . . T A	. . . . . . G . A . . . . . G T A . . .	[illegible]	[illegible]
051089|NA	B2	. . . . T A	. . . . . C G . . . . G T A . T .	[illegible]	[illegible]
043998|NA	B2	. . . . T A	. . . . . C G . . . . G T A . T .	[illegible]	[illegible]
RW937|HQ537764	B2	. . . . T A	. . . . . . G . . . . G T A . . .	[illegible]	[illegible]
RW754|HQ537765	B2	. . . . T A	. . . . . . G . . . . G T A . T .	[illegible]	[illegible]
Z094|HQ537777	C	. . . T . . C . . T A . . . G .	. . . . . . G . . . . G T A . T C	[illegible]	[illegible]
062097|NA	C	. . . T . . C . . T A . . . G .	. A . . . . G . . . . G T A . T C	[illegible]	[illegible]
050364|NA	C	. . . T . . C . . T A . . . G .	. A . . . . G . . . . G T A . T C	[illegible]	[illegible]
RW792|HQ537775	C	. . A T . . C . . T A . . . G .	. . . . . . G . . . . G T A . T C	[illegible]	[illegible]
RW644|HQ537776	C	. . . T . . C . . T A . . . G .	. . . . . . G . . . . G T A . T C	[illegible]	[illegible]
QV13816|HQ537774	C	. . . T . . . . . T A	. . . . . . G . . . . G T A . T C	[illegible]	[illegible]
QV34982|HQ537772	C	. . . T . . . . . T A	. . . . . . G . . . . G T A . T C	[illegible]	[illegible]
054176|NA	C	. . . T . . . . . T A	. . . . . . G . . . . G T A . T C	[illegible]	[illegible]
QV03666|HQ537773	C	. . . T . . . . . T A	. . . . . . G . . . . C . . G T A . T C	[illegible]	[illegible]
044033|NA	C	. . . T . . . . . T A	. . . . . . G . . . . G T A . T C	[illegible]	[illegible]
QV03858|HQ537766	D1	. C . . A . A . . T . . . . G .	G . . . . . G . C A . . . . G T A . T .	[illegible]	[illegible]
QV04732|HQ537767	D1	. . . . A . A . . T . . . . G .	G . . . . . G . C A . . . . G T A . T .	[illegible]	[illegible]
QV03841|HQ537768	D1	. . . . A . A . C T . . . . G .	G . . . . . G . C A . . . . G T A . T .	[illegible]	[illegible]
P138|NA	D1	. . . . . . A . . T . . . . G .	. . . . . . G . C A . . . . G T A . T .	[illegible]	[illegible]
064436|NA	D2	. . . . . . A C . T . . . . . G	. . . . . . G . . A G . . . G T A . T .	[illegible]	[illegible]
051402|NA	D2	. . . . . . A C . T . . . C G G	. . . . . . G . . A G . . . G T A . T .	[illegible]	[illegible]
RW841|HQ537769	D2	. . . . . . A C . T . . . C G G	. . . . . . G . . A G . . . G T A . T .	[illegible]	[illegible]
RW697|HQ537770	D2	. . . . . . A C . T . . . C G G	. . . . . . G . . A G . . . G T A . T .	[illegible]	[illegible]
RW63|HQ537771	D2	. . . . . . A C . T . . . C G G	. . . . . . G . . A G . . . G T A . T .	[illegible]	[illegible]

		E6	E7	E1	
APOBEC3-induced change		. *	. . . . . *	. . * . . . . * . . . * . . . . * . . . . . *	
Amino acid site (prototype)		18 32 46 86 89 93 95 97	5 9 14 20 41 63 64 69 73 74 76 77	8 24 26 27 34 50 57 58 61 62 79 93 98 115 125 160 170 172 176 180 181 245 263 271 294 308 317 325 334 346 361 367 370 384 400 409 418 507 511 589 590 596 604 628 639 642	4 14 18 108 109 151 176 183 215
PPH58|D90400	A1	A E V D E K C N	N R D T G G T C T T D V	N I R R D F S T A E I S E N I E V S V N T K D L P Q T V D D D D Y A V E W S L A N K S I P I	I K I K C E K L T
SC165|FJ385265	A1	. . . . . N . .	. . . .	. . . .	. . . .
SC147|FJ385263	A1	. . . . . N . .	. . . .	. . . . V	. . . .
SC100|FJ385261	A1	. . . .	. . . .	. . . .	. . . .
LZCC86|EU918765	A1	. . . .	. . . .	 V	. . M . . . . . .
SC144|FJ385264	A1	. . . .	. . . .	. . . . K	. . . .
SC101|FJ385262	A1	. . . . . N . .	. . . . A	. . . .	. . . .
SC174|FJ385266	A1	. . . . . N . .	. K . . . . A	. . . . S	. . . .
SC185|FJ385267	A1	. . . . K N . .	. . . . A	. . . . L	. . . .
TW60|NA	A1	. . . . . N . .	. . . . A	. . . .	. . . .
CNZJ3|KC860270	A1	. . . . . N . .	. . . . A	. . . .	. . . .
QE190|NA	A1	. . . . . N . .	. . . . A	. . . .	. . . .
SC78|FJ385268	A1	. . . . . N . .	. . . . A	. . . .	. . . .
058771|NA	A1	. . . .	. . . .	. . . . Q	. . . .
047402|NA	A1	. . . .	. . . .	. . . . Q	. . . .
QV00861|HQ537755	A2	. . . .	. . . .	. . . . Q . . . . G	. . . .
K1712|NA	A2	. . . .	. . . . R D	. . . . Q	. . . .
K3505|NA	A2	. . . .	. . . . R D	. . . . Q	. . . .
JP0221|AB819275	A2	. . . .	. . . . R D	. . . . Q	. . . .
QV03554|HQ537754	A2	. . . .	. . G . R D . R	. . . . Q . . . . A	. . . N D . . . .
JP1352|AB819278	A2	. . . .	. . . . R D	. . . . Q	. . . .
052265|NA	A2	. . . .	. . . . R D	. . . . Q	. . . .
051406|NA	A2	. . . .	. . . . R D	. . . . Q	. . . .
RW791|HQ537753	A2	. . . .	. . . . R D	. . . . Q	. . . .
CNZJ1|KC860269	A2	. . . .	. . . . R D	. . . . Q	. . . .
K20|NA	A2	. . . .	. . . . R D	. . . . Q	. . . .
K38|NA	A2	. . . .	. . . . R D	. . . . Q	. . . .
K643|NA	A2	. . . .	. . . . R D	. . . . Q	. . . .
JP0891|AB819277	A2	. . . .	. . . . R D	. . . . Q	. . . . V .
CNZJ2|KC860271	A2	. . . .	. . . . R D	. . . . Q	. . . .
K53|NA	A2	. . . .	. . . . R D	. . . . Q	. . . .
K2118|NA	A2	. . . .	. . . . R D	. . . . Q	. . . .
K421|NA	A2	. . . .	. . . . R D	. . . . Q	. . . .
JP0302|AB819276	A2	. . . .	. . . . R D	. . . . Q	. . . .
K25|NA	A2	. . . .	. . . . R D	. . . . Q . . . . S	. . . .
QV15606|HQ537752	A2	. . . .	. . . . R D	. . . . Q	. . . .
36A|KU298920	A2	. . . .	. . . . R D	. . . . Q	. . . .
TJ18|GQ472850	A2	. . . .	. . . . R D A . A . . .	. . . . Q . . . . H . . . . P	. . . .
QE1538|NA	A2	. . . .	. . . . R D	. . . . Q	. . . .
QV32351|HQ537757	A3	. . . .	. . . I . S	. . . .	. . . .
QV15563|HQ537756	A3	. . . .	. . . I . S	. . . . R	. . . .
AS347|HQ537760	A3	. . . .	. . . I . S	. . . .	. . . .
K2546|NA	A3	. . . .	. . . I . S	. . . .	. . . .
K8|NA	A3	. . . .	. . . I . S	. . . .	. . . .
K613|NA	A3	. . . .	. . . I . S	. . . . Q	. . . .
K191|NA	A3	. . . .	. . . I . S	. . . .	. . . .
K1374|NA	A3	. . . .	. . . I . S	. . . .	. . . .
JP1870|AB819279	A3	. . . .	. . . I . S	. . . .	. . . .
K34|NA	A3	. . . .	. . . I . S	. . P K . V	. . . .
K3823|NA	A3	. . . .	. . . I . S	. . . . K	. . . .
K99|NA	A3	. . . .	. . . I . S	. . . .	. . . .
K2277|NA	A3	. . . .	. . . I . S	. . . .	. . . .
K64|NA	A3	. . . .	. . . I . S	. . . .	. . . .
K43|NA	A3	. . . .	. . . I . S	. . . .	. . . .
AS405|HQ537759	A3	. . . .	. . . I . S	. . . .	. . . .
QE150|NA	A3	. . . .	. . . I . S	. . . .	. . . .
THA468|NA	A3	. . . .	. . . I . S	K	. . . .
K2550|NA	A3	. . . .	. . . I . S	. . . .	. . . .
K11|NA	A3	. . . .	. . . I . S	. . . .	. . . .
K3762|NA	A3	. . . .	. . . I . S	. . . .	. . . .
J739|NA	A3	. . . .	. K . I . S	. . . .	. . . . . . R . .
QE470|NA	A3	. . . .	. . . I . S	. . . .	. . . .
QE1132|NA	A3	. . . .	. . . I . S	. . . .	. . . .
QV00961|HQ537758	A3	. . . .	. . . I . S	. V . . . . I . . . . A . . . . . H	. . . .
Z023|HQ537763	B1	. Q . E . N . .	. . . . . S . . . A E .	. . . . N . . . . C . . . . E . . . . T . V . .	M . . . .
BF134|HQ537762	B1	. Q . E	. . . . . S . . . A E .	. . . . C . . . . T . V . .	M . . . .
BF077|HQ537761	B1	. Q . E	. . . . . S . . . A E .	. . . . C . . . . E . . . . T . V . .	M . . . . K . . .
051089|NA	B2	. . . E	. . . . A E .	. . . . C . . . . E . . . . I . . T	. . . .
043998|NA	B2	. . . E	. . . . A E .	. . . . C . . . . N E . . . . I . . T	. . . .
RW937|HQ537764	B2	. . . E	. . . . A E .	. . . . C . . . . E . . . . I . . T	. . . .
RW754|HQ537765	B2	. . . E	. . . . A E .	. . . . C . . . . E . . . . I . . T	. . . .
Z094|HQ537777	C	. . L E . . . D	. . . . A E .	. . . K . . . . A C . T S . . . . E . E . . . I . . . . . . T	. . . .
062097|NA	C	. . L E . . . D	. K . . . . A E .	. . . . A C . T S . . . . E . E . . . I . . . . . . T	. . . .
050364|NA	C	. . L E . . . D	. K . . . . A E .	. . . . A C . T S . . . . E . E . . . I . . . . . . T	. . . .
RW792|HQ537775	C	T . L E . . . D	. . . . A E .	. . . . A C . T S . . . . E . . . . T	. . . .
RW644|HQ537776	C	. . L E . . . D	. . . . A E .	. . . . A C . T S . . . . E . . . . T	. . . .
QV13816|HQ537774	C	. . . E	. . . . A E .	. . . . A C . T S . . . . E . . . . T	. R . . . .
QV34982|HQ537772	C	. . . E	. . . . A E .	. . . . A C . T S . . . . E . . . . T	. R . . . .
054176|NA	C	. . . E	. . . . A E .	. . . . A C . T S . . . . E . . . . T	. R . . . .
QV03666|HQ537773	C	. . . E	. . . . A E .	. . . . . . G . . . . A C . T S . . . . E . . . . V D T	. R . . . .
044033|NA	C	. . . E	. . . . A E .	. . . . A C . T S . . . . E . . . . T	. R . . . .
QV03858|HQ537766	D1	. . I . . . . D	S . . . . H . . . A E .	. . . . D R . . . . . . C . . S . G S . . . . . . E . . . . I . . T	. . . .
QV04732|HQ537767	D1	. . I . . . . D	S . . . . H . . . A E .	. . . . D R . . . . . . C . . S . . . . E . . . . . R . I . . T	. . . .
QV03841|HQ537768	D1	. . I . . . . D	S . . . . H . . . A E .	. . . . D R . . . . . . C . . S . . . L . . . . . E . . . . I . . T	. . . .
P138|NA	D1	. . I . . . . D	. . . . . H . . . A E .	. . . . D R . . K . . . C . . S . . . . E . . . . I . . T	. . . .
064436|NA	D2	. . T . . . . K	. . . . . D A . . A E .	. . . . A . . R . . . . . . C . . S . . . . E . . . . I . . T . . S .	. . . .
051402|NA	D2	. . T . . . R E	. . . . . D A . . A E .	. . . . A . . . . C . . S . . . . E . . . . I . . T	. . . .
RW841|HQ537769	D2	. . T . . . R E	. . . . . D A . . A E .	. . . . A . . . . C . . S . . . . E . . . . I . . T	. . . .
RW697|HQ537770	D2	. . T . . . R E	. . . . . D A . . A E .	. . . . A . . . . C . . S . . . . E . . . . I . . T	. . . .
RW63|HQ537771	D2	. . T . . . R E	. . . . . D A . . A E .	. . . . A . . R . . . . . . C . . S . . . . E . . . . I . . T C . . .	 I

Table S2. (cont.)

		E2	E4	NCR1	E5	NCR2	
APOBEC3-induced change		*** * * *	*** *		*	*	* * * **
Nucleotide site (prototype)		33333333333333333333333333333333<br>44444444555555555666666666777778<br>11334499225567899133556678125672<br>16085805590721756314192765211424	3333333333333333333<br>3344444444555555555<br>3911334499225567899<br>1616085805590721756	3333<br>8888<br>4589<br>6910	3333333333444444444<br>8999999999000000011<br>9033445578233347901<br>7601392788903874316	444444444444444444<br>111111111111111112<br>223333355788889990<br>131568238047902384	4444444444444444444444444444444444444444444444444445<br>2233333344444455555555566666666666677777777779999990<br>7913445923789901334577902245566678801234479990033480<br>2760284672055890493105791743907880141944860450915520
PPH58|D90400	A1	AAAGCTTAGATAGGTCGCTACGTCGATAACAT	TCAAAGCTTAGATAGGTCG	CAGC	ATTTATCAATGGCACACAA	ACTTCGGGG-TTATATAT	ACGTCTCGGGGATAGTGCGCGCAAATCCCTCTCCTCTCCCCCTCCTGCAACG
SC165|FJ385265	A1	..............G.................	................G..	....	....	.........-........	....
SC147|FJ385263	A1	..............G.................	................G..	....	....	.........-........	....
SC100|FJ385261	A1	......C.......G.................	........C.......G..	....	..............G....	....T...A-.......G	....
LZCC86|EU918765	A1	......C.......G.................	........C.......G..	.G..	....	.........-.......G	....C.........
SC144|FJ385264	A1	..............G...............C.	................G..	....	.............C...G.	.........-........	..................................G.................
SC101|FJ385262	A1	..........C...G.T.....C.........	............C...G.T	....	........C.T.T......	.........-........	........................CC..........................
SC174|FJ385266	A1	..........C...G.T.....C.........	............C...G.T	....	........C.T.T......	.........-........	........................CC..........................
SC185|FJ385267	A1	..........C...G.T.....CT........	............C...G.T	....	........C.T.T......	.........-........	........................CC..........................
TW60|NA	A1	..G.......C...G.T.....C.........	....G.......C...G.T	....	........C.T.T......	.........-........	........................CC..........................
CNZJ3|KC860270	A1	..G.......C...G.T.....C.........	....G.......C...G.T	....	........C.T.T......	.........-........	........................CC..........................
QE190|NA	A1	..........C...G.T.....C.........	C...........C...G.T	....	........C.T.T......	.........-........	........................CC..........................
SC78|FJ385268	A1	..........C...G.......C.........	C...........C...G..	....	........C.T.T......	.........-........	..........A.............CC..........................
058771|NA	A1	..............G.T...............	................G.T	....	T...C.......T......	.........-........	.........................C..........................
047402|NA	A1	..............G.T...............	................G.T	....	T...C.......T......	.........-........	.........................C..........................
QV00861|HQ537755	A2	....G.....CG..G.T...............	......G.....CG..G.T	T...	.....CA..C..T......	....A....-G..C....	.........................C.......................G..
K1712|NA	A2	....G.....C...G.T...............	......G.....C...G.T	....	.....C...C..T......	....A....-........	.........................C.......................G..
K3505|NA	A2	....G.....C...G.T...............	......G.....C...G.T	....	.....C...C..T......	....A....-........	.........................C.......................G..
JP0221|AB819275	A2	....G.....C...G.T...............	......G.....C...G.T	....	.....C...C..T......	....A....-........	.........................C.......................G..
QV03554|HQ537754	A2	....G.....C...G.T...............	......G.....C...G.T	....	.....C...C..T......	....A....-........	.................G....G..C.......................G..
JP1352|AB819278	A2	....G.....C...G.T...............	......G.....C...G.T	....	.....C...C..T......	....A....-........	.........................C.......................G..
052265|NA	A2	....G.....C...G.T...............	......G.....C...G.T	....	.....C...C..T......	....A....-........	.........................C.......................G..
051406|NA	A2	....G.....C...G.T..........G....	......G.....C...G.T	....	.....C...C..T......	....A....-........	..............A..........C.......................G..
RW791|HQ537753	A2	....G.....C...G.T...............	......G.....C...G.T	....	.....C...C..T......	....A....-........	.......................G.C.......................G..
CNZJ1|KC860269	A2	....G.....C...G.T...............	......G.....C...G.T	....	.....C...C..T......	....A....-........	.........................C.......................G..
K20|NA	A2	....G.....C...G.T...............	......G.....C...G.T	....	.....C...C..T......	....A....-........	....................A....C.......................G..
K38|NA	A2	....G.....C...G.T...............	......G.....C...G.T	....	.....C...C..T......	....A....-........	.........................C.......................G..
K643|NA	A2	....G.....C...G.T...............	......G.....C...G.T	....	.....C...C..T......	....A....-......C.	.........................C.......................G..
JP0891|AB819277	A2	....G.....C...G.T...............	......G.....C...G.T	....	.....C...C..T......	....A....-........	.........................C.......................G..
CNZJ2|KC860271	A2	....G.....C...G.T...............	......G.....C...G.T	....	.....C...C..T......	....A....-........	.........................C.......................G..
K53|NA	A2	....G.....C...G.T...............	......G.....C...G.T	....	.....C...C..T......	....A....-........	.........................C.......................G..
K2118|NA	A2	....G.....C...G.T...............	......G.....C...G.T	....	.....C...C..T......	....A....-........	.........................C.......................G..
K421|NA	A2	....G.....C...G.T...............	......G.....C...G.T	....	.....C...C..T......	....A....-........	.........................C.......................G..
JP0302|AB819276	A2	....G.....C...G.T...............	......G.....C...G.T	....	.....C...C..T......	....A....-........	.........................C.......................G..
K25|NA	A2	....G.....C...G.T...............	......G.....C...G.T	....	.....C...C..T......	....A....-........	.........................C.......................G..
QV15606|HQ537752	A2	....G.....C...G.T...............	......G.....C...G.T	....	.....C...C..T......	....A....-........	.........................C.......................G..
36A|KU298920	A2	....G.....C...G.T...............	......G.....C...G.T	....	.....C...C..T......	....A....-........	.........................C.......................G..
TJ18|GQ472850	A2	....G.....C...G.TAGT....A.......	......G.....C...G.T	..C.	.....C...C..T......	....A....-........	.........................C.......................G..
QE1538|NA	A2	....G.....C...G.T...............	......G.....C...G.T	....	.....C...C..T......	....A....-........	.........................C.......................G..
QV32351|HQ537757	A3	..........C...G.T........G......	............C...G.T	....	............T......	.........-....C...	....................A..G.C......................C...
QV15563|HQ537756	A3	..........C...G.T........G......	............C...G.T	....	............T......	.........-....C...	....................A..G.C......................C...
AS347|HQ537760	A3	..........C...G.T........G......	............C...G.T	....	............T......	.........-....C...	....................A..G.C......................C...
K2546|NA	A3	..............G.................	................G..	....	............T......	........A-....C...	....................A..G.C......................C...
K8|NA	A3	....G.....C...G.T........G......	......G.....C...G.T	....	............T......	.........-....C...	....................A..G.C......................C...
K613|NA	A3	....T.....C...G.T........G......	......T.....C...G.T	....	............T......	.........-....C...	....................A..G.C......................C...
K191|NA	A3	....T.....C...G.T........G......	......T.....C...G.T	....	............T......	.........-....C...	....................A..G.C......................C...
K1374|NA	A3	....T.....C...G.T........G......	......T.....C...G.T	....	............T......	.........-....C...	....................A..G.C......................C...
JP1870|AB819279	A3	....T.....C...G.T........G......	......T.....C...G.T	....	............T......	.........-....C...	....................A..G.C......................C...
K34|NA	A3	....T.....C...G.T........G......	......T.....C...G.T	....	............T......	.........-....C...	....................A..G.C......................C...
K3823|NA	A3	....T.....C...G.T........G......	......T.....C...G.T	....	............T......	.........-....C...	....................A..G.C......................C...
K99|NA	A3	....T.....C...G.T........G......	......T.....C...G.T	....	............T......	.........-....C...	....................A..G.C......................C...
K2277|NA	A3	....T.....C...G.T........G......	......T.....C...G.T	....	............T......	.........-....C...	....................A..G.C......................C...
K64|NA	A3	....T.....C...G.T........G......	......T.....C...G.T	....	............T......	.........-....C...	....................A..G.C......................C...
K43|NA	A3	....T.....C...G.T........G......	......T.....C...G.T	....	............T......	.........-....C...	....................A..G.C......................C...
AS405|HQ537759	A3	........A.C...G.T........G......	..........A.C...G.T	....	............T......	.........-....C...	....................A..G.C......................C...
QE150|NA	A3	....T.....C...G.T........G......	......T.....C...G.T	....	............T......	.........-....C...	....................A..G.C......................C.T.
THA468|NA	A3	....T.....C...G.T........G......	......T.....C...G.T	....	............T......	.........-....C...	....................A..G.C......................C...
K2550|NA	A3	....T.....C...G.T........G......	......T.....C...G.T	....	............T......	.........-....C...	....................A..G.C......................C...
K11|NA	A3	....T.....C...G.T........G......	......T.....C...G.T	....	............T......	.........-....C...	....................A..G.C......................C...
K3762|NA	A3	....T.....C...G.T........G......	......T.....C...G.T	....	............T......	.........-....C...	....................A..G.C......................C...
J739|NA	A3	....T.....C...G.T........G.....G	......T.....C...G.T	....	............T......	.........-....C...	........A...........A..G.C......................C...
QE470|NA	A3	....T.....C...G.T........G......	......T.....C...G.T	....	............T......	.........-....C...	....................A..G.C......................C...
QE1132|NA	A3	....T.....C...G.T........GG.....	......T.....C...G.T	....	............T......	.........-....C...	..T.................A..G.C......................C..A
QV00961|HQ537758	A3	..........C...G.T........G......	............C...G.T	....	............T......	.........-....C...	....................A..G.C......................C...
Z023|HQ537763	B1	.............CG.T...........GA..	...............CG.T	....	.......G....T......	.A..T....-........	.T....A..A...G..A........C..........C........GA..G..
BF134|HQ537762	B1	.............CG.T...........GA..	...............CG.T	..C.	.....C.G....T......	.A..T....-........	.T....A..A...G..A........C..........C........GA..G..
BF077|HQ537761	B1	.............CG.T...........GA..	...............CG.T	..C.	.....C.G....T......	.A..T....-........	.T....A..A...G..A........C..........C........GA..G..
051089|NA	B2	..........C.CTG.T....A......GA..	............C.CTG.T	....	.......G....T......	.AGT.....T........	....A.A..A.....C..A......C......A...C............G..
043998|NA	B2	..........C.CTGTT...........GA..	............C.CTGTT	....	.......G....T......	.AGT.....T........	....A.A..A.....CA.A......C......A...C............G..
RW937|HQ537764	B2	..........C.CTG.T............A..	............C.CTG.T	....	.......G....T......	.AGT.....T.G......	....A.A..A......A.A..T...C..........C............G..
RW754|HQ537765	B2	...A......C.CTG.T...........GA..	.....A......C.CTG.T	....	.......G....T......	.AGT.....T........	....A.A..A.....CA.A......C..........C............G..
Z094|HQ537777	C	.C........C..TG.T...G.......GA..	...C........C..TG.T	....	..C....G....T.T....	GAGT.....T........	....A.A..A.....CA.A......C..........C......TG....G..
062097|NA	C	.C........C..TG.T...G.......GA..	...C........C..TG.T	....	..C....G....T.T....	GAGT.....T........	....A.A..A.C...CA.A......C..........C.......G....G..
050364|NA	C	.C........C..TG.T...G.......GA..	...C........C..TG.T	....	..C....G....T.T....	GAGT.....T........	....A.A..A.C...CA.A......C..........C.......G....G..
RW792|HQ537775	C	G.........C..TG.T...........GA..	..G.........C..TG.T	...T	..C....G..A.T.T....	.AGT.....T........	....A.A..A.....CA.A......C..........C.......G....G..
RW644|HQ537776	C	G.........C..TG.T...........GA..	..G.........C..TG.T	...T	..C....G..A.T.T....	.AGT.....T........	....A.A..A.....CA.A......C..........C.......G....G..
QV13816|HQ537774	C	G.........C..TG.T...........GA..	..G.........C..TG.T	....	..C....G....T.T....	.AGT.....T........	....A.A..A.....CA.A......C.........AC......TG....G..
QV34982|HQ537772	C	G.........C..TG.T...........GA..	..G.........C..TG.T	....	..C....G....T.T....	.AGT.....T........	....A.A..A.....CA.A......C..........C.......G....G..
054176|NA	C	G.........C..TG.T...........GA..	..G.........C..TG.T	....	..C....G....T.T....	.AGT.....T........	....A.A..A.....CA.A......C..........C.......G....G..
QV03666|HQ537773	C	G......G..C..TG.T...........GA..	..G......G..C..TG.T	....	..C....G....T.T....	.AGT.....T........	....A.A..A.....CA.A......C..........C.......G....G..
044033|NA	C	G.........C..TG.T...........GA..	..G.........C..TG.T	....	..C....G....T.T....	.AGT.....T........	T...A.A..A.....CA.A......C..........C.......G....G..
QV03858|HQ537766	D1	.....G...GC..TG.............GA..	.......G...GC..TG..	....	..C....G...AT.T.T..	.AA.TA...T..G.....	...CAAAA.A.....CA.A......C..........CA......G..G.G..
QV04732|HQ537767	D1	.....G....C..TG.............GA..	.......G....C..TG..	....	..C....G....T.TT...	.AA.TA...T..G.....	...CAAA..A.....CA.A......C..........CA......G....G..
QV03841|HQ537768	D1	.....G....C..TG.............GA..	.......G....C..TG..	....	..C....G....T.T....	.AA.TA...T..G.....	...CAAA..A.....CA.A......C..........CA......G....G..
P138|NA	D1	..........C..TG.T...........GA..	............C..TG.T	....	.G.....G....T.T...G	.A..T..C.T..G..G..	...CA.A..A.....CA.A......C..........CA......G....G..
064436|NA	D2	..........C..TG.T...........GA..	............C..TG.T	....	..C....G....T.T....	.AGT..T..T..G.....	....A.A..A..G..CA.A......C..........CA......G....G..
051402|NA	D2	..........C..TG.............GA..	............C..TG..	....	..CC...G....T.T....	.AGT..T..T..G.....	....A.A..A..G..CA.A......C..........CA......G....G..
RW841|HQ537769	D2	..........C..TG.............GA..	............C..TG..	....	..C....G....T.T....	.AGT..T..T..G.....	....A.A..A..G..CA.A......CGT........CAG.TT..G....G..
RW697|HQ537770	D2	..........C..TG.............GA..	............C..TG..	....	..C....G....T.T....	.AGT..T..T..G.....	....A.A..A..G..CA.A......CGT...A....CAG.TT..G....G..
RW63|HQ537771	D2	..........C..TG.T...........GA..	.T..........C..TG.T	....	..C....G....T.T....	.AGT..T..T..G.....	....A.A..A..G..CA.AG.....CGTTCAA.T..CAGTT...G....G..

		E2	E4	E5	
APOBEC3-induced change		* * * *	***	*	** **
Amino acid site (prototype)		220 222 229 232 248 258 269 279 282 287 293 294 300 303 308 338 340 358	01 23 28 29 34 37 39 40 54 56 66 67 74 78 81 86 89	5 18 47 52 68	10 25 62 76 81 89 99 103 111 134 137 139 142 146 154 164 169 178 183 184 230 231 247 253
PPH58|D90400	A1	NSRDDGKSVDCLPDMLLL	LPTKQDSIITEDVGCFS	IIAFH	KGGSERPSSSTPLSTTLSFSRNHD
SC165|FJ385265	A1	.......A..........	...............L.	.....	....
SC147|FJ385263	A1	.......A..........	...............L.	.....	....
SC100|FJ385261	A1	.......A..........	........T......L.	...L.	....
LZCC86|EU918765	A1	.......A..........	........T......L.	.....	..................L.....
SC144|FJ385264	A1	.......A........F.	...............L.	.....	....
SC101|FJ385262	A1	.......AL.........	............A..L.	.....	....
SC174|FJ385266	A1	.......AL.........	............A..L.	.....	....
SC185|FJ385267	A1	.......AL.........	............A..L.	.....	....
TW60|NA	A1	.......AL.........	....R.......A..L.	.....	....
CNZJ3|KC860270	A1	.......AL.........	....R.......A..L.	.....	....
QE190|NA	A1	.......AL.........	S...........A..L.	.....	....
SC78|FJ385268	A1	.......A..........	S...........A..L.	.....	...N....................
058771|NA	A1	.......AL.........	...............L.	.L...	....
047402|NA	A1	.......AL.........	...............L.	.L...	....
QV00861|HQ537755	A2	......EAL.........	......W.....A..L.	.....	....
K1712|NA	A2	.......AL.........	......W.....A..L.	.....	....
K3505|NA	A2	.......AL.........	......W.....A..L.	.....	....
JP0221|AB819275	A2	.......AL.........	......W.....A..L.	.....	....
QV03554|HQ537754	A2	.......AL.........	......W.....A..L.	.....	......R.................
JP1352|AB819278	A2	.......AL.........	......W.....A..L.	.....	....
052265|NA	A2	.......AL.........	......W.....A..L.	.....	....
051406|NA	A2	.......AL.........	......W.....A..L.	.....	.....H..................
RW791|HQ537753	A2	.......AL.........	......W.....A..L.	.....	....
CNZJ1|KC860269	A2	.......AL.........	......W.....A..L.	.....	....
K20|NA	A2	.......AL.........	......W.....A..L.	.....	....
K38|NA	A2	.......AL.........	......W.....A..L.	.....	....
K643|NA	A2	.......AL.........	......W.....A..L.	.....	....
JP0891|AB819277	A2	.......AL.........	......W.....A..L.	.....	....
CNZJ2|KC860271	A2	.......AL.........	......W.....A..L.	.....	....
K53|NA	A2	.......AL.........	......W.....A..L.	.....	....
K2118|NA	A2	.......AL.........	......W.....A..L.	.....	....
K421|NA	A2	.......AL.........	......W.....A..L.	.....	....
JP0302|AB819276	A2	.......AL.........	......W.....A..L.	.....	....
K25|NA	A2	.......AL.........	......W.....A..L.	.....	....
QV15606|HQ537752	A2	.......AL.........	......W.....A..L.	.....	....
36A|KU298920	A2	.......AL.........	......W.....A..L.	.....	....
TJ18|GQ472850	A2	.......ALEWF..I...	......W.....A..L.	.....	....
QE1538|NA	A2	.......AL.........	......W.....A..L.	.....	....
QV32351|HQ537757	A3	.......AL.........	............A..L.	.....	.....................T..
QV15563|HQ537756	A3	.......AL.........	............A..L.	.....	.....................T..
AS347|HQ537760	A3	.......AL.........	............A..L.	.....	.....................T..
K2546|NA	A3	.......A..........	...............L.	.....	.....................T..
K8|NA	A3	.......AL.........	......W.....A..L.	.....	.....................T..
K613|NA	A3	.......AL.........	......L.....A..L.	.....	.....................T..
K191|NA	A3	.......AL.........	......L.....A..L.	.....	.....................T..
K1374|NA	A3	.......AL.........	......L.....A..L.	.....	.....................T..
JP1870|AB819279	A3	.......AL.........	......L.....A..L.	.....	.....................T..
K34|NA	A3	.......AL.........	......L.....A..L.	.....	.....................T..
K3823|NA	A3	.......AL.........	......L.....A..L.	.....	.....................T..
K99|NA	A3	.......AL.........	......L.....A..L.	.....	.....................T..
K2277|NA	A3	.......AL.........	......L.....A..L.	.....	.....................T..
K64|NA	A3	.......AL.........	......L.....A..L.	.....	.....................T..
K43|NA	A3	.......AL.........	......L.....A..L.	.....	.....................T..
AS405|HQ537759	A3	.....E.AL.........	..........K.A..L.	.....	.....................T..
QE150|NA	A3	.......AL.........	......L.....A..L.	.....	.....................TY.
THA468|NA	A3	.......AL.........	......L.....A..L.	.....	.....................T..
K2550|NA	A3	.......AL.........	......L.....A..L.	.....	.....................T..
K11|NA	A3	.......AL.........	......L.....A..L.	.....	.....................T..
K3762|NA	A3	.......AL.........	......L.....A..L.	.....	.....................T..
J739|NA	A3	.......AL........V	......L.....A..L.	.....	..R..................T..
QE470|NA	A3	.......AL.........	......L.....A..L.	.....	.....................T..
QE1132|NA	A3	.......AL.........	......L.....A..L.	.....	.C...................T.N
QV00961|HQ537758	A3	.......AL.........	............A..L.	.....	.....................T..
Z023|HQ537763	B1	.......AL......M..	..............SL.	.....	....
BF134|HQ537762	B1	.......AL......M..	..............SL.	.....	....
BF077|HQ537761	B1	.......AL......M..	..............SL.	.....	....
051089|NA	B2	.......AL....N.M..	............AAFL.	.....	....
043998|NA	B2	.......AL......M..	............AAFLF	.....	....
RW937|HQ537764	B2	.......AL......M..	............AAFL.	.....	........F...............
RW754|HQ537765	B2	..Q....AL......M..	.....N......AAFL.	.....	....
Z094|HQ537777	C	.R.....AL...R..M..	...N........A.FL.	.....	...................L....
062097|NA	C	.R.....AL...R..M..	...N........A.FL.	.....	....A...................
050364|NA	C	.R.....AL...R..M..	...N........A.FL.	.....	....A...................
RW792|HQ537775	C	S......AL......M..	..A.........A.FL.	.....	....
RW644|HQ537776	C	S......AL......M..	..A.........A.FL.	.....	....
QV13816|HQ537774	C	S......AL......M..	..A.........A.FL.	.....	..............N....L....
QV34982|HQ537772	C	S......AL......M..	..A.........A.FL.	.....	....
054176|NA	C	S......AL......M..	..A.........A.FL.	.....	....
QV03666|HQ537773	C	S...G..AL......M..	..A......A..A.FL.	.....	....
044033|NA	C	S......AL......M..	..A.........A.FL.	.....	M.......................
QV03858|HQ537766	D1	...E.X.A.......M..	.......S...GA.FL.	..T.Y	....................G...
QV04732|HQ537767	D1	...E.X.A.......M..	.......S....A.FL.	.....	....
QV03841|HQ537768	D1	...E.X.A.......M..	.......S....A.FL.	.....	....
P138|NA	D1	.......AL......M..	............A.FL.	M....	....
064436|NA	D2	.......AL......M..	............A.FL.	.....	....
051402|NA	D2	.......A.......M..	............A.FL.	.....	....
RW841|HQ537769	D2	.......A.......M..	............A.FL.	.....	.........CI....SFF......
RW697|HQ537770	D2	.......A.......M..	............A.FL.	.....	.........CI.I..SFF......
RW63|HQ537771	D2	.......AL......M..	.S..........A.FL.	.....	.......C.CILNF.SF.......

Table S2. (cont.)

Isolate	Subtype	L2	L1
APOBEC3-induced change		`       *  * *  *          *    * **                  `	`       *       *  *                            **     *        *      *               * *     `
Nucleotide site (prototype)		55555555555555555555555555555555555555555555555555555	5555555555555555555555666666666666666666666666666666666666666666666666666666666666667777777777
		00000000111112222222233333333333344444445555555555556	5556677788888888999999000000000011222334444444444455555555555566666667777778888889990000001111
		33455556011470366669902245677788903456890013334578891	7891648900123468346799112334556845026470133455555900112334556804888990114992226682571133782233
		03711144439346356790536970645818517197943510690289633	9633679819984910981246040896162408628714649004589603781698480171038276136782780916040667984835
PPH58|D90400	A1	GGT---ACATATCACAGCAACTATCTGGACCCGTAACAGTACCAAAATAAACG	AACGGTTCAAAACACGGAGGGAAAACAGCAATTAGAAAGGATAAGTCGGTCATTATAAATAAGGTACGGAGTAGAGCAAGAAGTAAGAAGAGAA
SC165|FJ385265	A1	...---...............C...............................	.............................G..
SC147|FJ385263	A1	...---...C.....	..............................G...
SC100|FJ385261	A1	...---...	..................A...C.......
LZCC86|EU918765	A1	...---...	......................G...G.....................
SC144|FJ385264	A1	...---.......G..................C.....A..............	....G...............
SC101|FJ385262	A1	...---..........................C.....A..............	....G.................................
SC174|FJ385266	A1	...---..........................C.....A..............	....G.................................
SC185|FJ385267	A1	...---..........................C.....A..............	....G.G.................................
TW60|NA	A1	...---..........................C.....A..............	....G.....A...........................
CNZJ3|KC860270	A1	...---..........................C.....A..............	....G.....A...........................
QE190|NA	A1	...---..........................C.....A..............	.....................................T......................G.....A...........................
SC78|FJ385268	A1	...---..........................C.....A..............	..............................G.............................G.....A...........................
058771|NA	A1	...---..........................C.....A..............	.........C..A..........C............................A.............A............C..............
047402|NA	A1	...---..........................C.....A..............	.........C..A..........C............................A.............A..G.........C..............
QV00861|HQ537755	A2	...---G.G.GG....A...............C.....A....C.........	.......................C................GC..............G......A..............................
K1712|NA	A2	...----.G.GG.G..A...............C.....A.......C......	.......................C................GC..............G......A..............................
K3505|NA	A2	...----.G.GG.G..A...............C.....A.......C......	.......................C................GC..............G......A..............................
JP0221|AB819275	A2	...----.G.GG....A...............C.....A.......C......	.......................C................GC..............G......A..............................
QV03554|HQ537754	A2	...----.G.GG....A..............TC.....A.......C.C....	.......................C................GC...C.....G....G......A..................AG..........
JP1352|AB819278	A2	...----.G.GG.T..A...............C.....A.......C......	.......................C................GC..............G......A..............................
052265|NA	A2	...ATA..G.GG....A...............C.....A.......C......	.......................C................GC..............G......A.......................G......
051406|NA	A2	...ATA..G.GG....A...............C.....A.......C......	.......................C................GC..............G......A.......................G......
RW791|HQ537753	A2	...----.G.GG....A...............C.....A.......C......	.......................C................GC..............G......A..............................
CNZJ1|KC860269	A2	...----.G.GG....A...........G...C.....A.......C......	.......................C................GC..............G......A..................A...........
K20|NA	A2	...----.G.GG....A...............C.G...A.......C......	.......T...............C................GC..............G...GG.A..............................
K38|NA	A2	...----.G.GG....A...............C.G...A.......C......	.......T...............C................GC..............G...G..A..............................
K643|NA	A2	...----.G.GG....A...............C.G...A.......C......	.......T...............C................GC..............G...G..A..............................
JP0891|AB819277	A2	...----.G.GG....A...............C.G...A.......C......	.......................C................GC..............G...G..A..............................
CNZJ2|KC860271	A2	...----.G.GG....A............T..C.G...A.......C......	.......................C................GC..............G...G..A............T.................
K53|NA	A2	...----.G.GG....A...............C.G...A.......C......	..........G............C................GC..............G......A.......C......................
K2118|NA	A2	...----.G.GG....A...............C.G...A.......C......	..........G............C................GC..............G......A.......C......................
K421|NA	A2	...----.G.GG....A.........A.....C.G...A.......C......	.......................C................GC..............G......A..............................
JP0302|AB819276	A2	...----.G.GG....A.........A.....C.G...A.......C......	.......................C................GC..............G......A..............................
K25|NA	A2	...----.G.GG....A.........A.....C.G...A.......C......	.......................C................GC..............G......A..............................
QV15606|HQ537752	A2	...----.G.GG....A...............C.....A.......C......	.......................C..............A.GC..............G......A..............................
36A|KU298920	A2	...----.G.GG....A...............C.....A......GC......	.......................C................GC..............G......C..............................
TJ18|GQ472850	A2	...----.G.GG..T.A...............C.....A.......C......	.......................C............G...GC....T.........G......A..............................
QE1538|NA	A2	...----.G.GG....A...............C....CA..............	...........G...........C................GC............G.G......A..............................
QV32351|HQ537757	A3	...---..........A...............C.....A..........C...	C....C.................C................................G...................A.................
QV15563|HQ537756	A3	...---..........A...............C.....A..........C...	C....C.................C................................G...................A.................
AS347|HQ537760	A3	...---..........A...............C.....A..........C...	C....C.................C................................G...................A..........C......
K2546|NA	A3	...---..........A...............C.....A..........C...	C....C.................C.........G......................G...................A.................
K8|NA	A3	...---..........A...............C.....A..........C...	C....C.................C.........G......................G...................A.................
K613|NA	A3	...---..........A...............C.....A..TA......C...	C....C.................C.........G......................G...................A.................
K191|NA	A3	...---..........A...............C.....A.....T....C...	C....C.................C..........................T.....G...................A.................
K1374|NA	A3	...---..........A...............C.....A.....T....C...	C....C.................C..........................T.....G...................A.................
JP1870|AB819279	A3	...---..........A...............C.....A.C........C...	C....C.................C..........................A.....G...................A.................
K34|NA	A3	...---..........A...............C.....A.C........C...	C....C.................C..........................A.....G...................A.................
K3823|NA	A3	...---..........A...............C.....A..........C...	C....C.................C..........................A.....G...................A.................
K99|NA	A3	...---..........A...............C.....A..........C...	C....C.................C..........................A.....G...................A.................
K2277|NA	A3	...---..........A...............C.....A..........C...	C....C.................C................................G...................A.................
K64|NA	A3	...---..........A...............C.....A..........C...	C....C.................C................................G...................A.................
K43|NA	A3	...---..........A...............C.....A..........C...	C....C.................C................................G...................A.............G...
AS405|HQ537759	A3	...---......T...A...............C.....A..........C...	C....C.................C................................G...................A.................
QE150|NA	A3	...---..........A...............C.....A..........C...	C....C.................C........G.......................G...................A.................
THA468|NA	A3	.A.---..........A........C......C.....A..........C...	C....C.................C................................G...................A....G............
K2550|NA	A3	...---..........A........C......C.....A..........C...	C....C.................C................................G...................A....G............
K11|NA	A3	...---..........A........C......C.....A..........C...	C....C.................C................................G...................A.................
K3762|NA	A3	...---..........A........C......C.....A..........C...	C....C.................C................................G...................A.................
J739|NA	A3	...---..........A........C......C.....A..........C...	C....C.................C................................G.......G........A..A.................
QE470|NA	A3	...---..........A...............C.....A..........C...	C....C.................C...A......................T.....G.................G.A...G.............
QE1132|NA	A3	...---..........A...............C.....A..........C...	C....C.................C..........................T.....G.................G.A...G............G
QV00961|HQ537758	A3	...---..........A....C..........C.....A..........C...	C....C.........A.G.....C................................G........G..........A...G.............
Z023|HQ537763	B1	...----.G......GAG..A.C....C..A.C...T.A..............	....A...G..........AAC.CG..............A...G............G.................GAAG..........GA....
BF134|HQ537762	B1	...----.G.......AG..A..C...C..A.C...T.A..............	....A...G..........AA..CG..............A...G............G.................GAAG..........GA....
BF077|HQ537761	B1	...----.G.......AG..A..C...C..A.C...T.A..............	....A...G..........AA..CG.G............A...G............G.................GAAG..........GA..C.
051089|NA	B2	...----.G.......AG.........C..G.C.....A........G.....	......C.G...........A......A......AG...A.......T.CT.....G.........A.......G.AG..........G..A..
043998|NA	B2	...----.G.......AG.........C..A.C.....A........G.....	......C.G...........A.............AG...A.......T.CT.....G.........A.......GAAG................
RW937|HQ537764	B2	...----.G.......AG.........C..A.C.....A........G.....	......C.G...........A..........C...G...A.......T.CT.......................GAAG................
RW754|HQ537765	B2	...----.G.......AG.........C..A.C.....A........G.....	......C.G...........A..........C...G...A.......T.CT.......................GAAG................
Z094|HQ537777	C	...----.G.......AG.........C..A.C.....A............T.	..T.....G.....G.A...A....TG.A..........A..CCC..AAG.................AA.A...GAAG.......T...A....
062097|NA	C	...----TG.......AG.........C..A.C.....A............T.	..T.....G.....G.A...A....TG.A..........A..CCC..AAG.................AA.A...GAAG.......T...A....
050364|NA	C	...----TG.......AG.........C..A.C.....A............T.	..T.....G.....G.A...A....TG.A..........A..CCC..AAG.................AA.A...GAAG.......T...A....
RW792|HQ537775	C	...----.G.......AG.........C..A.C.....A............T.	..T...........G.A...A....TG.A..........A..CCC...AG.................AA.A...GAAG.......G...A....
RW644|HQ537776	C	...----.G.......AG.........C..A.C.....A............T.	..T...........G.A...A....TG.A..........A..CCC...AG.................AA.A...GAAG.......G...A....
QV13816|HQ537774	C	...----.G.......AG.........C..A.C..T..A............T.	..T...........G.A...A....TG.A..........A..CCC...AG.................AA.A...GAAG.......G...A....
QV34982|HQ537772	C	...----.G.......AG.........C..A.C..T..A............TT	..TT..........G.A...A....TG.A..........A..CCC...AG.................AA.A...GAAG.......G...A....
054176|NA	C	...----.G.......AG.........C..A.C.....A............T.	..T...........G.A...A....TG.A..........A..CCC...AG.................AA.A...GAAG.......G...A....
QV03666|HQ537773	C	...----.G.......AG.........C..A.CC....A............T.	..T...........G.A...A....TG.A..........A..CCC...AG.....C...C..A....AA.A...GAAG.......G...A....
044033|NA	C	...----.G.......AG.........C..A.C.....A............T.	..T.......G...G.A...A....TG.A..........A..CCC..AAG.................AA.A...GAAG.......G...A....
QV03858|HQ537766	D1	..C---..G.......AGC.....G..C..A.C.....A............GT	.GT.......G.........A..................A.......A...........................AAG......GG..C.....
QV04732|HQ537767	D1	...----.G.......AG.........C..A.C.....A............T.	..T.......G.........A..................A.......A...........................AAG......GG..C.....
QV03841|HQ537768	D1	...----.G.......AG.........C..A.C.....A............T.	..T.......G.........A..................A.......A...........................AAG......GG..C.....
P138|NA	D1	A..----.G.......AG.........C..A.C.....A.......C....T.	..T.......G.........A..................A.......A...........................AAG......GG..C.....
064436|NA	D2	...---..........CG.........C..A.C.....A............T.	..T.......G.........A..................A.......A..................A.......GAAG......GG..C.....
051402|NA	D2	...----.G.......CG.........C..A.C.....A............T.	..T.......G.........A..................A.......A...................A......GAAG......GG..C.....
RW841|HQ537769	D2	...----.G.......CG.........C..A.C.....AA...........T.	..T.......G.........A..................A.......A.....C............AA......GAAG......GG..C.....
RW697|HQ537770	D2	...----.G.......CG.........C..A.C.....A............T.	..T.......G.........A......A...........A.......A...................A......GAAG......GG..C.....
RW63|HQ537771	D2	...----.GC......CG.........C..A.C.....A............T.	..T.......G..G......A..................A.......A.........C........A.......GAAG......GG..C.....

Isolate	Subtype	L2	L1
APOBEC3-induced change		`    **   *   *    *          `	`     **  *         *       *   *   `
Amino acid site (prototype)		22222333333333333334444444444	`     11111111222222233333333444455`
		66777124445567777880012233445	`  118034455662369999112477881222922`
		34014110291485789020661339687	58072634909130512679185858132021234
PPH58|D90400	A1	EDXSSRKQQLTSDDQSPLSTLASSTIMHR	LTADNDEVYLSRPIDLKAVDVFIGTGKDIDNRVKK
SC165|FJ385265	A1	.........F...................	....
SC147|FJ385263	A1	........................P....	....
SC100|FJ385261	A1	....	......K.........................P..
LZCC86|EU918765	A1	....	........C..........................
SC144|FJ385264	A1	.....................T.......	....
SC101|FJ385262	A1	.....................T.......	....
SC174|FJ385266	A1	.....................T.......	....
SC185|FJ385267	A1	.....................T.......	....
TW60|NA	A1	.....................T.......	........................N..........
CNZJ3|KC860270	A1	.....................T.......	........................N..........
QE190|NA	A1	.....................T.......	...............F........N..........
SC78|FJ385268	A1	.....................T.......	........................N..........
058771|NA	A1	.....................T.......	....T....F...........Y..N..........
047402|NA	A1	.....................T.......	....T....F...........Y..N.R........
QV00861|HQ537755	A2	...G.................T.......	.........F............M............
K1712|NA	A2	.....................T..P....	.........F............M............
K3505|NA	A2	.....................T..P....	.........F............M............
JP0221|AB819275	A2	.....................T..P....	.........F............M............
QV03554|HQ537754	A2	..................L..T..PL...	.........F........A...M............
JP1352|AB819278	A2	......N..............T..P....	.........F............M............
052265|NA	A2	..N..................T..P....	.........F............M............
051406|NA	A2	..N..................T..P....	.........F............M............
RW791|HQ537753	A2	.....................T..P....	.........F............M............
CNZJ1|KC860269	A2	...............G.....T..P....	.........F............M............
K20|NA	A2	.....................T..P....	.........F............M............
K38|NA	A2	.....................T..P....	.........F............M............
K643|NA	A2	.....................T..P....	.........F............M............
JP0891|AB819277	A2	.....................T..P....	.........F............M............
CNZJ2|KC860271	A2	................S....T..P....	.........F............M............
K53|NA	A2	.....................T..P....	.........F............M............
K2118|NA	A2	.....................T..P....	.........F............M............
K421|NA	A2	.............N.......T..P....	.........F............M............
JP0302|AB819276	A2	.............N.......T..P....	.........F............M............
K25|NA	A2	.............N.......T..P....	.........F............M............
QV15606|HQ537752	A2	.....................T..P....	.........F............M............
36A|KU298920	A2	.....................T..P....	.........F............M............
TJ18|GQ472850	A2	.....................T..P....	.........F....G.......M............
QE1538|NA	A2	.....................T.......	.........F............M............
QV32351|HQ537757	A3	.....................T....L..	F........F............M............
QV15563|HQ537756	A3	.....................T....L..	F........F............M............
AS347|HQ537760	A3	.....................T....L..	F........F............M............
K2546|NA	A3	.....................T....L..	F........F............M............
K8|NA	A3	.....................T....L..	F........F............M............
K613|NA	A3	.....................TFY..L..	F........F............M............
K191|NA	A3	.....................T....L..	F........F............M............
K1374|NA	A3	.....................T....L..	F........F............M............
JP1870|AB819279	A3	.....................T....L..	F........F............M............
K34|NA	A3	.....................T....L..	F........F............M............
K3823|NA	A3	.....................T....L..	F........F............M............
K99|NA	A3	.....................T....L..	F........F............M............
K2277|NA	A3	.....................T....L..	F........F............M............
K64|NA	A3	.....................T....L..	F........F............M............
K43|NA	A3	.....................T....L..	F........F............M............
AS405|HQ537759	A3	.....C...............T....L..	F........F............M............
QE150|NA	A3	.....................T....L..	F........F............M............
THA468|NA	A3	.N...................T....L..	F........F............M............
K2550|NA	A3	.....................T....L..	F........F............M............
K11|NA	A3	.....................T....L..	F........F............M............
K3762|NA	A3	.....................T....L..	F........F............M............
J739|NA	A3	.....................T....L..	F........F............M............
QE470|NA	A3	.....................T....L..	F........F.K..........M.....V......
QE1132|NA	A3	.....................T....L..	F........F............M.....V.....R
QV00961|HQ537758	A3	...........P.........T....L..	F....N...F............M............
Z023|HQ537763	B1	.......RE.N...H..M..FT.......	.......I.F............M.....VND....
BF134|HQ537762	B1	........E.N...H..M..FT.......	.......I.F............M.....VND....
BF077|HQ537761	B1	........E.N...H..M..FT.......	.......I.FG...........M.....VND.N..
051089|NA	B2	........E.....H..V...T.......	.......I...K.V......A.M.N...V.DI...
043998|NA	B2	........E.....H..M...T.......	.......I.....V......A.M.N...VND....
RW937|HQ537764	B2	........E.....H..M...T.......	.......I.....V......A.......VND....
RW754|HQ537765	B2	........E.....H..M...T.......	.......I.....V......A.......VND....
Z094|HQ537777	C	........E.....H..M...T.......	..V....I..G.T...TP.NG....D.NVND....
062097|NA	C	....L...E.....H..M...T.......	..V....I..G.T...TP.NG....D.NVND....
050364|NA	C	....L...E.....H..M...T.......	..V....I..G.T...TP.NG....D.NVND....
RW792|HQ537775	C	........E.....H..M...T.......	..V....I..G.T...TP.NG....D.NVND....
RW644|HQ537776	C	........E.....H..M...T.......	..V....I..G.T...TP.NG....D.NVND....
QV13816|HQ537774	C	........E.....H..MS..T.......	..V....I..G.T...TP.NG....D.NVND....
QV34982|HQ537772	C	........E.....H..MS..T......I	..VY...I..G.T...TP.NG....D.NVND....
054176|NA	C	........E.....H..M...T.......	..V....I..G.T...TP.NG....D.NVND....
QV03666|HQ537773	C	........E.....H..M...T.......	..V....I..G.T...TP.NG..D.D.NVND....
044033|NA	C	........E.....H..M...T.......	..V....I..G.T...TP.NG....D.NVND....
QV03858|HQ537766	D1	........D...E.H..M...T.....R.	.AV....I.................N.D....
QV04732|HQ537767	D1	........E.....H..M...T.......	..V....I.................N.D....
QV03841|HQ537768	D1	........E.....H..M...T.......	..V....I.................N.D....
P138|NA	D1	K.......E.....H..M...T..P....	..V....I.................N.D....
064436|NA	D2	.......HE.....H..M...T.......	..V....I................N...VND....
051402|NA	D2	.......HE.....H..M...T.......	..V....I....................VND....
RW841|HQ537769	D2	.......HE.....H..M...T.......	..V....I................N...VND....
RW697|HQ537770	D2	.......HE.....H..M...T.......	..V....I...K................VND....
RW63|HQ537771	D2	.......HE.....H..M...T.......	..V....I................N...VND....

Table S2. (cont.)

		LCR																																																																																																																																															
APOBEC3-induced change							*				*																						*											*			*				*	*												*																																																		*			*			*			*										*	*									
Nucleotide site (prototype)		7	7	7	7	7	7	7	7	7	7	7	7	7	7	7	7	7	7	7	7	7	7	7	7	7	7	7	7	7	7	7	7	7	7	7	7	7	7	7	7	7	7	7	7	7	7	7	7	7	7	7	7	7	7	7	7	7	7	7	7	7	7	7	7	7	7	7	7	7	7	7	7	7	7	7	7	7	7	7	7	7	7	7	7	7	7	7	7	7	7	7	7	7	7	7	7	7	7	7	7	7	7	7	7	7	7	7	7	7	7	7	7	7	7	7	7	7	7	7	7	7	7	7																		
		1	1	1	1	1	1	1	1	1	1	2	2	2	2	2	2	2	2	2	2	2	2	2	2	2	2	2	2	2	2	2	2	2	2	2	3	3	3	3	3	3	3	4	4	4	4	4	4	4	4	4	4	5	5	5	5	5	5	6	6	6	6	6	6	7	7	7	7	7	7	7	7	7	7	7	7	7	7	7	7	7	7	7	7	7	7	7	7	7	7	7	7	7	7	7	7	7	7	7	7	7	7	7	7	7	7	7	7	7	7	7	7	7	7	7	7	7	7	7	7	7	7	7	7	8																		
		4	4	6	7	7	8	8	8	9	9	0	0	2	2	3	3	4	5	6	6	6	6	6	6	6	6	6	6	6	6	6	6	7	8	9	0	1	3	4	6	8	9	0	1	2	2	3	3	6	6	8	9	2	2	2	3	3	4	1	4	5	7	8	8	0	1	1	1	1	1	1	1	1	1	1	1	1	1	1	1	1	1	1	1	1	1	1	1	1	1	1	1	1	1	1	1	1	1	1	1	1	1	1	1	1	1	1	1	1	1	1	1	1	1	3	4	4	5	5	7	7	8	8	8	8	9	9	9	0	1	1	1	2	3	3	5	5	5	5	6	8	8	8	8	8	8	9
		0	7	5	5	7	5	6	9	4	9	0	7	4	6	0	4	1	7	3	3	3	3	3	3	3	3	3	3	3	3	5	6	1	4	7	4	3	2	5	9	8	5	8	0	1	9	1	5	3	4	3	0	2	4	5	1	8	0	9	8	9	3	4	6	3	4	5	5	5	5	5	5	5	5	5	5	5	5	5	5	5	5	5	5	5	5	5	5	5	5	5	5	5	5	5	5	5	5	5	5	5	5	5	5	5	5	5	5	5	5	5	5	7	9	0	5	9	5	6	8	9	4	5	7	9	0	2	3	7	1	8	9	1	0	3	2	3	4	7	2	1	4	6	7	8	9	7
PPH58|D90400	A1	T	G	T	T	G	T	A	T	G	C	T	T	T	G	G	T	C	T	-	-	-	-	-	-	-	-	-	-	-	-	C	C	C	C	A	A	A	G	T	T	C	G	A	A	G	T	T	A	C	T	T	G	T	A	A	A	C	A	G	A	A	A	A	G	G	A	G	-	-	-	-	-	-	-	-	-	-	-	-	-	-	-	-	-	-	-	-	-	-	-	-	-	-	-	-	-	-	-	-	-	-	-	-	-	-	-	-	-	-	-	T	G	C	G	C	A	A	T	A	T	T	T	G	G	C	A	T	A	A	T	T	C	G	C	T	G	C	C	G	A	T	T	T	T	C
SC165|FJ385265	A1	.	.	.	.	.	.	.	.	.	.	.	.	.	.	.	.	.	.	-	-	-	-	-	-	-	-	-	-	-	-	.	.	.	.	.	.	.	.	.	.	.	.	.	.	.	.	.	.	.	.	.	.	.	.	.	.	.	.	.	.	.	.	.	.	.	.	.	-	-	-	-	-	-	-	-	-	-	-	-	-	-	-	-	-	-	-	-	-	-	-	-	-	-	-	-	-	-	-	-	-	-	-	-	-	-	-	-	-	-	-	.	.	.	.	.	.	.	.	.	.	.	.	.	.	.	.	.	.	.	.	.	.	.	.	.	.	.	.	.	.	.	.	.	.	.
SC147|FJ385263	A1	.	.	.	.	.	.	.	.	.	.	.	.	.	.	.	.	.	.	-	-	-	-	-	-	-	-	-	-	-	-	.	.	.	.	.	.	.	.	.	.	.	.	.	.	.	.	.	.	.	.	.	.	.	.	.	.	.	.	.	.	.	.	.	.	.	.	.	-	-	-	-	-	-	-	-	-	-	-	-	-	-	-	-	-	-	-	-	-	-	-	-	-	-	-	-	-	-	-	-	-	-	-	-	-	-	-	-	-	-	-	.	.	.	.	.	.	.	.	.	.	.	.	.	.	.	.	.	.	.	.	.	.	.	.	.	.	.	.	.	.	.	.	.	.	.
SC100|FJ385261	A1	.	.	.	.	.	.	.	.	.	.	.	.	.	.	.	.	.	.	-	-	-	-	-	-	-	-	-	-	-	-	.	.	.	.	.	.	.	.	.	.	.	.	.	.	.	.	.	.	.	.	.	.	.	.	.	.	.	.	.	.	.	.	.	.	.	.	.	-	-	-	-	-	-	-	-	-	-	-	-	-	-	-	-	-	-	-	-	-	-	-	-	-	-	-	-	-	-	-	-	-	-	-	-	-	-	-	-	-	-	-	.	.	.	.	.	.	.	.	.	.	.	.	.	.	.	.	.	.	.	.	.	.	.	.	.	.	.	.	.	.	.	.	.	.	.
LZCC86|EU918765	A1	.	.	.	C	.	.	.	.	.	.	.	.	.	.	.	.	.	.	-	-	-	-	-	-	-	-	-	-	-	-	.	.	.	.	.	.	.	.	.	.	.	.	.	.	.	.	.	.	.	.	.	.	.	.	.	.	.	.	.	.	.	.	.	.	.	.	.	-	-	-	-	-	-	-	-	-	-	-	-	-	-	-	-	-	-	-	-	-	-	-	-	-	-	-	-	-	-	-	-	-	-	-	-	-	-	-	-	-	-	-	.	.	.	.	.	.	.	.	.	.	.	.	.	.	.	.	.	.	.	.	.	.	.	.	.	.	.	.	.	.	.	.	.	.	.
SC144|FJ385264	A1	.	.	.	.	.	.	.	.	.	.	.	.	.	.	.	.	.	.	-	-	-	-	-	-	-	-	-	-	-	-	.	.	.	.	.	.	.	.	.	.	.	.	.	.	.	.	.	.	.	.	.	.	.	.	.	.	.	G	.	.	.	.	.	.	.	.	.	-	-	-	-	-	-	-	-	-	-	-	-	-	-	-	-	-	-	-	-	-	-	-	-	-	-	-	-	-	-	-	-	-	-	-	-	-	-	-	-	-	-	-	.	.	.	.	G	.	.	.	.	C	.	.	.	.	.	.	.	.	.	.	.	.	.	.	.	.	.	.	.	.	.	.	.	.	.
SC101|FJ385262	A1	.	.	.	.	.	.	.	.	.	.	.	.	.	.	.	.	.	.	-	-	-	-	-	-	-	-	-	-	-	-	.	.	.	.	.	.	.	.	.	.	.	.	.	.	.	.	.	.	.	.	.	A	.	.	.	.	.	.	.	.	.	.	.	.	.	.	.	-	-	-	-	-	-	-	-	-	-	-	-	-	-	-	-	-	-	-	-	-	-	-	-	-	-	-	-	-	-	-	-	-	-	-	-	-	-	-	-	-	-	-	.	.	.	.	.	.	.	.	.	.	.	.	.	.	.	.	.	.	.	.	.	.	.	.	.	.	.	.	.	.	.	.	.	.	.
SC174|FJ385266	A1	.	.	.	.	.	.	.	.	.	.	A	.	.	.	.	.	.	.	-	-	-	-	-	-	-	-	-	-	-	-	.	.	.	.	.	.	.	.	.	.	.	.	.	.	.	.	.	.	.	G	.	.	.	.	.	.	.	.	.	.	.	.	.	.	.	.	.	-	-	-	-	-	-	-	-	-	-	-	-	-	-	-	-	-	-	-	-	-	-	-	-	-	-	-	-	-	-	-	-	-	-	-	-	-	-	-	-	-	-	-	.	.	.	.	.	.	.	.	.	.	.	.	.	.	.	.	G	.	.	.	.	.	.	.	.	.	.	.	.	.	.	.	.	.	.
SC185|FJ385267	A1	.	.	.	.	.	.	.	.	.	.	.	.	.	.	.	.	.	.	T	C	C	T	T	G	T	C	A	G	T	T	.	.	.	.	.	.	.	.	.	.	.	.	.	.	.	.	.	.	.	.	.	.	.	.	.	.	.	.	.	.	.	.	.	.	.	.	.	-	-	-	-	-	-	-	-	-	-	-	-	-	-	-	-	-	-	-	-	-	-	-	-	-	-	-	-	-	-	-	-	-	-	-	-	-	-	-	-	-	-	-	.	.	.	.	.	.	.	.	.	.	.	.	.	.	.	.	.	.	.	.	.	.	.	.	.	.	.	.	.	.	.	.	.	.	.
TW60|NA	A1	.	.	.	.	.	.	.	.	.	.	.	.	.	.	.	.	.	.	-	-	-	-	-	-	-	-	-	-	-	-	.	.	.	.	.	.	.	.	.	.	.	.	.	.	.	.	.	.	.	.	.	.	.	.	.	.	.	.	.	.	.	.	.	.	.	.	.	-	-	-	-	-	-	-	-	-	-	-	-	-	-	-	-	-	-	-	-	-	-	-	-	-	-	-	-	-	-	-	-	-	-	-	-	-	-	-	-	-	-	-	.	.	.	.	.	.	.	.	.	.	.	.	.	.	.	G	.	.	.	.	.	.	.	.	.	.	.	.	.	.	.	.	.	.	.
CNZJ3|KC860270	A1	.	.	.	.	.	.	.	.	.	.	.	.	.	.	.	.	.	.	-	-	-	-	-	-	-	-	-	-	-	-	.	.	.	.	.	.	.	.	.	.	.	.	.	.	.	.	.	.	.	.	.	.	.	.	.	.	.	.	.	.	.	.	.	.	.	.	.	-	-	-	-	-	-	-	-	-	-	-	-	-	-	-	-	-	-	-	-	-	-	-	-	-	-	-	-	-	-	-	-	-	-	-	-	-	-	-	-	-	-	-	.	.	.	.	.	.	.	.	.	.	.	.	.	.	.	G	.	.	.	.	.	.	.	.	.	.	.	.	C	.	.	.	.	.	.
QE190|NA	A1	.	.	.	.	.	.	.	.	.	.	.	.	.	.	.	.	.	.	-	-	-	-	-	-	-	-	-	-	-	-	.	.	.	.	.	.	.	.	.	.	.	.	.	.	C	.	.	.	.	.	.	.	.	.	.	.	.	.	.	.	.	.	.	.	.	.	.	-	-	-	-	-	-	-	-	-	-	-	-	-	-	-	-	-	-	-	-	-	-	-	-	-	-	-	-	-	-	-	-	-	-	-	-	-	-	-	-	-	-	-	.	.	.	.	.	.	.	.	.	.	.	.	.	.	.	G	.	.	.	.	.	.	.	.	.	.	.	.	.	.	.	.	.	.	.
SC78|FJ385268	A1	.	.	.	.	.	.	.	.	.	.	.	.	.	.	.	.	.	.	-	-	-	-	-	-	-	-	-	-	-	-	.	.	.	.	.	.	.	.	.	.	.	.	.	.	.	.	.	.	.	.	.	.	.	.	.	.	T	.	.	.	.	.	.	.	.	.	.	-	-	-	-	-	-	-	-	-	-	-	-	-	-	-	-	-	-	-	-	-	-	-	-	-	-	-	-	-	-	-	-	-	-	-	-	-	-	-	-	-	-	-	.	.	.	.	.	.	.	.	.	.	.	.	.	.	.	G	.	.	.	.	.	.	.	.	.	.	.	.	.	.	C	.	.	.	.
058771|NA	A1	.	.	.	.	.	.	.	.	.	.	.	.	.	.	.	.	.	.	-	-	-	-	-	-	-	-	-	-	-	-	.	.	.	.	.	.	.	.	.	.	.	.	.	.	.	.	.	.	.	.	.	.	.	.	.	.	.	.	.	.	.	C	.	.	.	.	.	-	-	-	-	-	-	-	-	-	-	-	-	-	-	-	-	-	-	-	-	-	-	-	-	-	-	-	-	-	-	-	-	-	-	-	-	-	-	-	-	-	-	-	.	.	.	.	.	.	.	.	.	.	.	.	.	.	.	.	.	.	.	.	.	.	.	.	.	.	.	.	.	.	.	.	.	.	.
047402|NA	A1	.	.	.	.	.	.	.	.	.	.	.	.	.	.	.	.	.	.	-	-	-	-	-	-	-	-	-	-	-	-	.	.	.	.	.	.	.	.	.	.	.	.	.	.	.	.	.	.	.	.	.	.	.	.	.	.	.	.	.	.	.	C	.	.	.	.	.	-	-	-	-	-	-	-	-	-	-	-	-	-	-	-	-	-	-	-	-	-	-	-	-	-	-	-	-	-	-	-	-	-	-	-	-	-	-	-	-	-	-	-	.	.	.	.	.	.	.	.	.	.	.	.	.	.	.	.	.	.	.	.	.	.	.	.	.	.	.	.	.	.	.	.	.	.	.
QV00861|HQ537755	A2	.	.	.	.	.	.	.	.	.	.	.	.	.	.	.	.	.	.	-	-	-	-	-	-	-	-	-	-	-	-	.	T	.	.	.	.	.	.	.	.	.	.	.	.	.	.	.	.	.	.	.	.	.	.	.	.	.	.	.	.	.	.	.	.	.	G	.	-	-	-	-	-	-	-	-	-	-	-	-	-	-	-	-	-	-	-	-	-	-	-	-	-	-	-	-	-	-	-	-	-	-	-	-	-	-	-	-	-	-	-	.	.	.	.	.	.	.	.	.	.	.	.	.	.	.	.	.	.	.	.	C	.	.	.	.	.	.	.	.	.	.	.	.	.	.
K1712|NA	A2	.	.	G	.	.	.	.	.	.	.	.	.	.	.	.	.	.	.	-	-	-	-	-	-	-	-	-	-	-	-	.	T	.	.	.	.	.	.	.	.	.	.	.	.	.	.	.	.	.	.	G	.	.	.	.	.	.	.	.	.	.	.	.	.	.	G	.	-	-	-	-	-	-	-	-	-	-	-	-	-	-	-	-	-	-	-	-	-	-	-	-	-	-	-	-	-	-	-	-	-	-	-	-	-	-	-	-	-	-	-	.	.	.	.	.	.	.	.	.	.	.	.	.	.	.	.	.	.	.	.	.	.	.	T	.	.	.	.	.	.	.	.	.	.	.
K3505|NA	A2	.	.	G	.	.	.	.	.	.	.	.	.	.	.	.	.	.	.	-	-	-	-	-	-	-	-	-	-	-	-	.	T	.	.	.	.	.	.	.	.	.	.	.	.	.	.	.	.	.	.	G	.	.	.	.	.	.	.	.	.	.	.	.	.	.	G	.	-	-	-	-	-	-	-	-	-	-	-	-	-	-	-	-	-	-	-	-	-	-	-	-	-	-	-	-	-	-	-	-	-	-	-	-	-	-	-	-	-	-	-	.	.	.	.	.	.	.	.	.	.	.	.	.	.	.	.	.	.	.	.	.	.	.	T	.	.	.	.	.	.	.	.	.	.	.
JP0221|AB819275	A2	.	.	.	.	.	.	.	.	.	.	.	.	.	.	.	.	.	.	-	-	-	-	-	-	-	-	-	-	-	-	G	T	.	.	.	.	.	.	.	.	.	.	.	.	.	.	.	.	.	.	.	.	.	.	.	.	.	.	.	.	.	.	.	.	.	G	.	-	-	-	-	-	-	-	-	-	-	-	-	-	-	-	-	-	-	-	-	-	-	-	-	-	-	-	-	-	-	-	-	-	-	-	-	-	-	-	-	-	-	-	.	.	.	.	.	.	.	.	.	.	.	.	.	.	.	.	.	.	.	.	.	.	.	.	.	.	.	.	.	.	.	.	.	.	.
QV03554|HQ537754	A2	.	.	.	.	.	.	.	.	.	.	.	.	C	.	.	.	.	.	-	-	-	-	-	-	-	-	-	-	-	-	G	T	.	.	.	.	.	.	.	.	.	.	.	.	.	.	.	.	.	.	.	.	.	.	.	.	.	.	.	.	.	.	.	.	.	G	.	-	-	-	-	-	-	-	-	-	-	-	-	-	-	-	-	-	-	-	-	-	-	-	-	-	-	-	-	-	-	-	-	-	-	-	-	-	-	-	-	-	-	-	.	.	.	.	.	.	.	.	.	.	.	.	.	.	.	.	.	.	.	.	.	.	.	.	.	.	.	.	.	.	.	.	.	.	.
JP1352|AB819278	A2	.	.	.	.	.	.	.	.	.	.	.	.	.	.	.	.	.	.	-	-	-	-	-	-	-	-	-	-	-	-	G	T	.	.	.	.	.	.	.	.	.	.	.	.	.	.	.	.	.	.	.	.	.	.	.	.	.	.	.	.	.	.	.	.	.	G	.	-	-	-	-	-	-	-	-	-	-	-	-	-	-	-	-	-	-	-	-	-	-	-	-	-	-	-	-	-	-	-	-	-	-	-	-	-	-	-	-	-	-	-	.	.	.	.	.	.	C	.	.	.	.	.	.	.	.	.	.	.	.	.	.	.	.	.	.	.	.	.	.	.	.	.	.	.	.
052265|NA	A2	.	.	.	.	.	.	.	.	.	.	.	.	.	.	.	.	.	.	-	-	-	-	-	-	-	-	-	-	-	-	G	T	.	.	.	.	.	.	.	.	.	.	C	.	.	.	.	.	.	.	.	.	G	.	.	.	.	.	.	.	.	.	.	.	.	G	.	-	-	-	-	-	-	-	-	-	-	-	-	-	-	-	-	-	-	-	-	-	-	-	-	-	-	-	-	-	-	-	-	-	-	-	-	-	-	-	-	-	-	-	.	.	.	.	.	.	.	.	.	.	.	.	.	.	.	.	C	.	.	.	.	.	.	.	.	.	.	.	.	.	.	.	.	.	.
051406|NA	A2	.	.	.	.	.	.	.	.	.	.	.	.	.	.	.	.	.	.	-	-	-	-	-	-	-	-	-	-	-	-	G	T	.	.	.	.	.	.	.	.	.	.	C	.	.	.	.	.	.	.	.	.	G	.	.	.	.	.	.	.	.	.	.	.	.	G	.	-	-	-	-	-	-	-	-	-	-	-	-	-	-	-	-	-	-	-	-	-	-	-	-	-	-	-	-	-	-	-	-	-	-	-	-	-	-	-	-	-	-	-	.	.	.	.	.	.	.	.	.	.	.	.	.	.	.	.	C	.	.	.	.	.	.	.	.	.	.	.	.	.	.	.	.	.	.
RW791|HQ537753	A2	.	.	.	.	A	.	.	.	.	.	.	.	.	.	.	.	.	.	-	-	-	-	-	-	-	-	-	-	-	-	G	T	.	.	.	.	.	.	.	.	.	.	.	.	.	.	.	.	.	.	.	.	G	.	.	.	.	.	.	.	.	.	.	.	.	G	.	-	-	-	-	-	-	-	-	-	-	-	-	-	-	-	-	-	-	-	-	-	-	-	-	-	-	-	-	-	-	-	-	-	-	-	-	-	-	-	-	-	-	-	.	.	.	.	.	.	.	.	.	.	.	.	.	.	.	.	.	.	.	.	.	.	.	.	.	.	.	.	.	.	.	.	.	.	.
CNZJ1|KC860269	A2	.	.	.	.	.	.	.	.	.	.	.	.	.	.	.	.	.	.	-	-	-	-	-	-	-	-	-	-	-	-	.	T	.	.	.	.	.	.	.	.	.	.	.	.	.	.	.	.	.	.	.	.	.	.	.	.	.	.	.	.	.	.	.	.	.	G	.	-	-	-	-	-	-	-	-	-	-	-	-	-	-	-	-	-	-	-	-	-	-	-	-	-	-	-	-	-	-	-	-	-	-	-	-	-	-	-	-	-	-	-	.	.	.	.	.	.	.	.	.	.	.	.	.	.	.	.	.	.	.	.	.	.	.	.	.	.	.	.	.	.	.	.	.	.	.
K20|NA	A2	.	.	.	.	.	.	.	.	.	.	.	.	.	.	.	.	.	.	-	-	-	-	-	-	-	-	-	-	-	-	.	T	.	.	.	.	.	.	.	.	.	.	.	.	.	.	.	.	.	.	.	.	.	.	.	.	.	.	.	.	.	.	.	.	.	G	.	-	-	-	-	-	-	-	-	-	-	-	-	-	-	-	-	-	-	-	-	-	-	-	-	-	-	-	-	-	-	-	-	-	-	-	-	-	-	-	-	-	-	-	.	A	.	.	.	.	.	.	.	.	.	.	.	.	.	.	.	.	.	.	.	.	.	.	.	A	.	.	.	.	.	.	.	.	.
K38|NA	A2	.	.	.	.	.	.	.	.	.	.	.	.	.	.	.	.	.	.	-	-	-	-	-	-	-	-	-	-	-	-	.	T	.	.	.	.	.	.	.	.	.	.	.	.	.	.	.	.	.	.	.	.	.	.	.	T	.	.	.	.	.	.	.	.	.	G	.	-	-	-	-	-	-	-	-	-	-	-	-	-	-	-	-	-	-	-	-	-	-	-	-	-	-	-	-	-	-	-	-	-	-	-	-	-	-	-	-	-	-	-	.	A	.	.	.	.	.	.	.	.	.	.	.	.	.	.	.	.	.	.	.	.	.	.	.	A	.	.	.	.	.	.	.	.	.
K643|NA	A2	.	.	.	.	.	.	.	.	.	.	.	.	.	.	.	.	.	.	-	-	-	-	-	-	-	-	-	-	-	-	.	T	.	.	.	.	.	.	.	.	.	.	.	.	.	.	.	.	.	.	.	.	.	T	.	.	.	.	.	.	.	.	.	.	.	G	.	-	-	-	-	-	-	-	-	-	-	-	-	-	-	-	-	-	-	-	-	-	-	-	-	-	-	-	-	-	-	-	-	-	-	-	-	-	-	-	-	-	-	-	.	A	.	.	.	.	.	.	.	.	.	.	.	.	.	.	.	.	.	.	.	.	.	.	.	A	.	.	.	.	.	.	.	.	.
JP0891|AB819277	A2	.	.	.	.	.	.	.	.	.	.	.	.	.	.	.	.	.	.	-	-	-	-	-	-	-	-	-	-	-	-	.	T	.	.	.	.	.	.	.	.	.	.	.	.	.	.	.	.	.	.	.	.	.	.	.	.	.	.	.	.	.	.	.	.	.	G	.	-	-	-	-	-	-	-	-	-	-	-	-	-	-	-	-	-	-	-	-	-	-	-	-	-	-	-	-	-	-	-	-	-	-	-	-	-	-	-	-	-	-	-	.	.	.	.	.	.	.	.	.	.	.	.	.	.	.	.	.	.	.	.	.	.	.	.	.	.	.	.	.	.	G	.	.	.	.
CNZJ2|KC860271	A2	.	.	.	.	.	.	.	.	.	.	.	.	.	.	.	.	.	.	-	-	-	-	-	-	-	-	-	-	-	-	.	T	.	.	.	.	.	.	.	.	.	.	.	.	.	.	.	.	.	.	.	.	.	.	.	.	.	.	.	.	.	.	.	.	.	G	.	-	-	-	-	-	-	-	-	-	-	-	-	-	-	-	-	-	-	-	-	-	-	-	-	-	-	-	-	-	-	-	-	-	-	-	-	-	-	-	-	-	-	-	.	.	.	.	.	.	.	.	.	.	.	.	.	.	.	.	.	.	.	.	.	.	.	.	.	.	.	.	.	.	.	.	.	.	.
K53|NA	A2	.	.	.	.	.	.	.	.	.	.	.	.	.	.	.	.	.	.	-	-	-	-	-	-	-	-	-	-	-	-	.	T	.	.	.	.	.	.	.	.	.	.	.	.	.	.	.	.	.	.	.	.	.	.	.	.	.	.	.	.	.	.	.	.	.	G	.	-	-	-	-	-	-	-	-	-	-	-	-	-	-	-	-	-	-	-	-	-	-	-	-	-	-	-	-	-	-	-	-	-	-	-	-	-	-	-	-	-	-	-	.	.	.	.	.	.	.	.	.	.	.	.	.	.	.	.	.	.	.	.	.	.	.	.	.	.	.	.	.	.	.	.	.	G	.
K2118|NA	A2	.	.	.	.	.	.	.	.	.	.	.	.	.	.	.	.	.	.	-	-	-	-	-	-	-	-	-	-	-	-	.	T	.	.	.	.	.	.	.	.	.	.	.	.	.	.	.	.	.	.	.	.	.	.	.	.	.	.	.	.	.	.	.	.	.	G	.	-	-	-	-	-	-	-	-	-	-	-	-	-	-	-	-	-	-	-	-	-	-	-	-	-	-	-	-	-	-	-	-	-	-	-	-	-	-	-	-	-	-	-	.	.	.	.	.	.	.	.	.	.	.	.	.	.	.	.	.	.	.	.	.	.	.	.	.	.	.	.	.	.	.	.	.	G	.
K421|NA	A2	.	.	.	.	.	.	.	.	.	.	.	.	.	.	.	.	.	.	-	-	-	-	-	-	-	-	-	-	-	-	.	T	.	.	.	.	.	.	.	.	.	.	.	.	.	.	.	.	.	.	.	.	.	.	.	.	.	.	.	.	.	.	.	.	.	G	.	-	-	-	-	-	-	-	-	-	-	-	-	-	-	-	-	-	-	-	-	-	-	-	-	-	-	-	-	-	-	-	-	-	-	-	-	-	-	-	-	-	-	-	.	.	.	.	.	.	.	.	.	.	.	.	.	.	.	.	.	.	.	.	.	.	.	.	C	.	.	.	.	.	.	.	.	.	.
JP0302|AB819276	A2	.	.	.	.	.	.	.	.	.	.	.	.	.	.	.	.	.	.	-	-	-	-	-	-	-	-	-	-	-	-	.	T	.	.	.	.	.	.	.	.	.	.	.	.	.	.	.	.	.	.	.	.	.	.	.	.	.	.	.	.	.	.	.	.	.	G	.	-	-	-	-	-	-	-	-	-	-	-	-	-	-	-	-	-	-	-	-	-	-	-	-	-	-	-	-	-	-	-	-	-	-	-	-	-	-	-	-	-	-	-	.	.	.	.	.	.	.	.	.	.	.	.	.	.	.	.	.	.	.	.	.	.	.	.	C	.	.	.	.	.	.	.	.	.	.
K25|NA	A2	.	.	.	.	.	.	.	.	.	.	.	.	.	.	.	.	.	.	-	-	-	-	-	-	-	-	-	-	-	-	.	T	.	.	.	.	.	.	.	.	.	.	.	.	.	.	.	.	.	.	.	.	.	.	.	.	.	.	.	.	.	.	.	.	.	G	.	-	-	-	-	-	-	-	-	-	-	-	-	-	-	-	-	-	-	-	-	-	-	-	-	-	-	-	-	-	-	-	-	-	-	-	-	-	-	-	-	-	-	-	.	.	.	.	.	.	.	.	.	.	.	.	.	.	.	.	.	.	.	.	.	.	.	.	C	.	.	.	.	.	.	.	.	.	.
QV15606|HQ537752	A2	.	.	.	.	.	.	.	.	.	.	.	.	.	.	.	.	.	.	-	-	-	-	-	-	-	-	-	-	-	-	.	T	.	.	.	.	.	.	.	.	.	.	.	.	.	.	.	.	.	.	.	.	.	.	.	.	.	.	.	.	.	.	.	.	.	G	.	-	-	-	-	-	-	-	-	-	-	-	-	-	-	-	-	-	-	-	-	-	-	-	-	-	-	-	-	-	-	-	-	-	-	-	-	-	-	-	-	-	-	-	.	.	.	.	.	.	.	.	.	.	.	.	.	.	.	.	.	.	.	.	.	.	.	.	.	.	.	.	.	.	.	.	.	.	.
36A|KU298920	A2	.	.	.	.	.	.	.	.	.	.	.	.	.	.	.	.	.	.	-	-	-	-	-	-	-	-	-	-	-	-	.	T	.	.	.	.	.	.	.	.	.	.	.	.	.	.	.	.	.	.	.	.	.	.	.	.	.	.	.	.	.	.	.	.	.	G	.	-	-	-	-	-	-	-	-	-	-	-	-	-	-	-	-	-	-	-	-	-	-	-	-	-	-	-	-	-	-	-	-	-	-	-	-	-	-	-	-	-	-	-	.	.	.	.	.	.	.	.	.	.	.	.	.	.	.	.	.	.	.	.	.	.	.	.	.	.	.	.	.	.	.	.	.	.	.
TJ18|GQ472850	A2	.	.	.	.	.	.	.	.	.	.	.	.	.	.	.	.	.	.	-	-	-	-	-	-	-	-	-	-	-	-	.	T	.	.	.	.	.	.	.	.	.	.	.	.	.	.	.	.	.	.	.	.	.	.	.	.	.	.	.	.	.	.	.	.	A	G	.	-	-	-	-	-	-	-	-	-	-	-	-	-	-	-	-	-	-	-	-	-	-	-	-	-	-	-	-	-	-	-	-	-	-	-	-	-	-	-	-	-	-	-	.	.	.	.	.	.	.	.	.	.	.	.	.	.	.	.	.	.	.	.	.	.	.	.	.	.	.	.	.	.	C	.	.	.	.
QE1538|NA	A2	.	.	.	.	.	.	.	.	.	.	.	.	.	.	.	.	.	.	-	-	-	-	-	-	-	-	-	-	-	-	.	T	.	.	.	.	.	.	.	.	.	.	.	.	.	.	.	.	.	.	.	.	.	.	.	.	.	.	.	.	.	.	.	.	.	G	.	-	-	-	-	-	-	-	-	-	-	-	-	-	-	-	-	-	-	-	-	-	-	-	-	-	-	-	-	-	-	-	-	-	-	-	-	-	-	-	-	-	-	-	.	.	.	.	.	.	.	.	.	.	.	.	.	.	.	.	.	.	.	.	.	.	.	.	.	.	.	.	.	.	.	.	C	.	.
QV32351|HQ537757	A3	.	T	.	.	.	.	.	.	C	.	.	.	.	.	.	.	.	.	T	C	C	T	T	G	T	C	A	G	T	T	.	.	.	.	.	G	.	.	.	.	.	.	.	G	.	.	.	.	.	.	.	.	.	.	.	.	.	.	.	.	.	.	.	.	.	C	.	-	-	-	-	-	-	-	-	-	-	-	-	-	-	-	-	-	-	-	-	-	-	-	-	-	-	-	-	-	-	-	-	-	-	-	-	-	-	-	-	-	-	-	.	.	.	.	.	G	.	.	.	.	.	.	.	.	.	.	.	.	.	.	.	.	.	T	.	.	.	.	.	.	.	.	.	.	.
QV15563|HQ537756	A3	.	T	.	.	.	.	.	.	C	.	.	.	.	.	.	.	.	.	T	C	C	T	T	G	T	C	A	G	T	T	.	.	.	.	.	G	.	.	.	.	.	.	.	G	.	.	.	.	.	.	.	.	.	.	.	.	.	.	.	.	.	.	.	.	.	C	.	-	-	-	-	-	-	-	-	-	-	-	-	-	-	-	-	-	-	-	-	-	-	-	-	-	-	-	-	-	-	-	-	-	-	-	-	-	-	-	-	-	-	-	.	.	.	.	.	G	.	.	.	.	.	.	.	.	.	.	.	.	.	.	.	.	.	T	.	.	.	.	.	.	.	G	.	.	.
AS347|HQ537760	A3	.	T	.	.	.	.	.	.	C	.	.	.	.	.	.	.	.	.	T	C	C	T	T	G	T	C	A	G	T	T	.	.	.	.	.	G	.	.	.	.	.	.	.	.	.	.	.	.	.	.	.	.	.	.	C	.	.	.	.	.	.	.	.	.	.	C	.	-	-	-	-	-	-	-	-	-	-	-	-	-	-	-	-	-	-	-	-	-	-	-	-	-	-	-	-	-	-	-	-	-	-	-	-	-	-	-	-	-	-	-	.	.	.	.	.	G	.	.	.	.	.	.	.	.	.	.	.	.	.	.	.	.	.	T	C	.	.	.	.	T	.	.	.	.	.
K2546|NA	A3	.	T	.	.	.	.	.	.	C	.	.	.	.	.	.	.	.	.	T	C	C	T	T	G	T	C	A	G	T	T	.	.	.	.	.	G	.	.	.	.	.	.	.	.	.	.	.	.	T	.	.	.	.	.	C	.	.	.	.	.	.	.	.	.	.	C	.	-	-	-	-	-	-	-	-	-	-	-	-	-	-	-	-	-	-	-	-	-	-	-	-	-	-	-	-	-	-	-	-	-	-	-	-	-	-	-	-	-	-	-	.	.	.	.	.	G	.	.	.	.	.	.	.	.	.	.	.	.	.	.	.	.	.	T	C	.	.	.	.	.	.	.	.	.	.
K8|NA	A3	.	T	.	.	.	.	.	.	C	.	.	.	.	.	.	.	.	.	T	C	C	T	T	G	T	C	A	G	T	T	.	.	.	.	.	G	.	.	.	.	.	.	.	.	.	.	.	.	T	.	.	.	.	.	C	.	.	.	.	C	.	.	.	.	.	C	.	-	-	-	-	-	-	-	-	-	-	-	-	-	-	-	-	-	-	-	-	-	-	-	-	-	-	-	-	-	-	-	-	-	-	-	-	-	-	-	-	-	-	-	.	.	.	.	.	G	.	.	.	.	.	.	.	.	.	.	.	.	.	.	.	.	.	T	C	.	.	.	.	.	.	.	.	.	.
K613|NA	A3	.	T	.	.	.	.	.	.	C	.	.	.	.	.	.	.	.	.	T	C	C	T	T	G	T	C	A	G	T	T	.	.	.	.	.	G	.	.	.	.	.	.	.	.	.	.	.	.	T	.	.	.	.	.	C	.	.	.	.	.	.	.	.	.	.	C	.	-	-	-	-	-	-	-	-	-	-	-	-	-	-	-	-	-	-	-	-	-	-	-	-	-	-	-	-	-	-	-	-	-	-	-	-	-	-	-	-	-	-	-	.	.	.	.	.	G	.	.	.	.	.	.	.	.	.	.	.	.	.	.	.	.	.	T	C	.	.	.	.	.	.	.	.	.	.
K191|NA	A3	.	T	.	.	.	.	.	.	C	.	.	.	.	A	.	.	.	.	T	C	C	T	T	G	T	C	A	G	T	T	.	.	.	.	.	G	.	.	.	.	.	.	.	.	.	.	.	.	T	.	.	.	.	.	C	.	.	.	.	.	.	.	.	.	.	C	.	-	-	-	-	-	-	-	-	-	-	-	-	-	-	-	-	-	-	-	-	-	-	-	-	-	-	-	-	-	-	-	-	-	-	-	-	-	-	-	-	-	-	-	.	.	.	.	.	G	.	.	.	.	.	.	.	.	.	.	.	.	.	.	.	.	.	T	C	.	.	.	.	.	.	.	.	.	.
K1374|NA	A3	.	T	.	.	.	.	.	.	C	.	.	.	.	.	.	.	.	.	T	C	C	T	T	G	T	C	A	G	T	T	.	.	.	.	.	G	.	.	.	.	.	.	.	.	.	.	.	.	T	.	.	.	.	.	C	.	.	.	.	.	.	.	.	.	.	C	.	-	-	-	-	-	-	-	-	-	-	-	-	-	-	-	-	-	-	-	-	-	-	-	-	-	-	-	-	-	-	-	-	-	-	-	-	-	-	-	-	-	-	-	.	.	.	.	.	G	.	.	.	.	.	.	.	.	.	.	.	.	.	.	.	.	.	T	C	.	.	.	.	.	.	.	.	.	.
JP1870|AB819279	A3	.	T	.	.	.	.	.	.	C	.	.	.	.	.	.	.	.	.	T	C	C	T	T	G	T	C	A	G	T	T	.	.	.	.	.	G	.	.	.	.	.	.	.	.	.	.	.	.	T	.	.	.	.	.	C	.	.	.	.	.	.	.	.	.	.	C	.	-	-	-	-	-	-	-	-	-	-	-	-	-	-	-	-	-	-	-	-	-	-	-	-	-	-	-	-	-	-	-	-	-	-	-	-	-	-	-	-	-	-	-	.	.	.	.	.	G	.	.	.	.	.	.	.	.	.	.	.	.	.	.	.	.	.	T	C	.	.	.	.	.	.	.	.	.	.
K34|NA	A3	.	T	.	.	.	.	.	.	C	.	.	.	.	.	.	.	.	.	T	C	C	T	T	G	T	C	A	G	T	T	.	.	.	.	.	G	.	.	.	.	.	.	.	.	.	.	.	.	T	.	.	.	.	.	C	.	.	.	.	.	.	.	.	.	.	C	.	-	-	-	-	-	-	-	-	-	-	-	-	-	-	-	-	-	-	-	-	-	-	-	-	-	-	-	-	-	-	-	-	-	-	-	-	-	-	-	-	-	-	-	.	.	.	.	.	G	.	.	.	.	.	.	.	.	.	.	.	.	.	.	.	.	.	T	C	.	.	.	.	.	.	.	.	.	.
K3823|NA	A3	.	T	.	.	.	.	.	.	C	.	.	.	.	.	.	.	.	.	T	C	C	T	T	G	T	C	A	G	T	T	.	.	.	.	.	G	.	.	.	.	.	.	.	.	.	.	.	.	T	.	.	.	.	.	C	.	.	.	.	.	.	.	.	.	.	C	.	-	-	-	-	-	-	-	-	-	-	-	-	-	-	-	-	-	-	-	-	-	-	-	-	-	-	-	-	-	-	-	-	-	-	-	-	-	-	-	-	-	-	-	.	.	.	.	.	G	.	.	.	.	.	.	.	.	.	.	.	.	.	.	.	.	.	T	C	.	.	.	.	.	.	.	.	.	.
K99|NA	A3	.	T	.	.	.	.	.	.	C	.	.	.	.	.	.	.	.	.	T	C	C	T	T	G	T	C	A	G	T	T	.	.	.	.	.	G	.	.	.	.	.	.	.	.	.	.	.	.	T	.	.	.	.	.	C	.	.	.	.	.	.	.	.	.	.	C	.	-	-	-	-	-	-	-	-	-	-	-	-	-	-	-	-	-	-	-	-	-	-	-	-	-	-	-	-	-	-	-	-	-	-	-	-	-	-	-	-	-	-	-	.	.	.	.	.	G	.	.	.	.	.	.	.	.	.	.	.	.	.	.	.	.	.	T	C	.	.	.	.	.	.	.	.	.	.
K2277|NA	A3	.	T	.	.	.	.	.	.	C	.	.	.	.	.	.	.	.	.	T	C	C	T	T	G	T	C	A	G	T	T	.	.	.	.	.	G	.	.	.	.	.	.	.	.	.	.	.	.	T	.	.	.	.	.	C	.	.	.	.	.	.	T	.	.	.	C	.	-	-	-	-	-	-	-	-	-	-	-	-	-	-	-	-	-	-	-	-	-	-	-	-	-	-	-	-	-	-	-	-	-	-	-	-	-	-	-	-	-	-	-	.	.	.	.	.	G	.	.	.	.	.	.	.	.	.	.	.	.	.	.	.	.	.	T	C	.	.	.	.	.	.	.	.	.	.
K64|NA	A3	.	T	.	.	.	.	.	.	C	.	.	.	.	.	.	.	.	.	T	C	C	T	T	G	T	C	A	G	T	T	.	.	.	.	.	G	.	.	.	.	.	.	.	.	.	.	.	.	T	.	.	.	.	.	C	.	.	.	.	.	.	T	.	.	.	C	.	-	-	-	-	-	-	-	-	-	-	-	-	-	-	-	-	-	-	-	-	-	-	-	-	-	-	-	-	-	-	-	-	-	-	-	-	-	-	-	-	-	-	-	.	.	.	.	.	G	.	.	.	.	.	.	.	T	.	.	.	.	.	.	.	.	.	T	C	.	.	.	.	.	.	.	.	.	.
K43|NA	A3	.	T	.	.	.	.	.	.	C	.	.	.	.	.	.	.	.	.	T	C	C	T	T	G	T	C	A	G	T	T	.	.	.	.	.	G	.	.	.	.	.	.	.	.	.	.	.	.	T	.	.	.	.	.	C	.	.	.	.	.	.	T	.	.	.	C	.	-	-	-	-	-	-	-	-	-	-	-	-	-	-	-	-	-	-	-	-	-	-	-	-	-	-	-	-	-	-	-	-	-	-	-	-	-	-	-	-	-	-	-	.	.	.	.	.	G	.	.	.	.	.	.	.	.	.	.	.	.	.	.	.	.	.	T	C	.	.	.	.	.	.	.	.	.	.
AS405|HQ537759	A3	.	T	.	.	.	.	.	.	C	.	.	.	.	.	.	.	.	.	T	C	C	T	T	G	T	C	A	G	T	T	.	.	.	.	.	G	.	.	.	.	.	.	.	.	.	.	.	.	.	.	.	.	.	.	C	.	.	.	.	.	.	.	.	.	.	C	.	-	-	-	-	-	-	-	-	-	-	-	-	-	-	-	-	-	-	-	-	-	-	-	-	-	-	-	-	-	-	-	-	-	-	-	-	-	-	-	-	-	-	-	.	.	.	.	.	G	.	.	.	.	.	.	.	.	.	C	.	.	.	.	.	.	.	T	.	.	.	.	.	.	.	.	.	.	.
QE150|NA	A3	.	T	.	.	.	.	.	.	C	.	.	.	.	.	.	.	.	.	T	C	C	T	T	G	T	C	A	G	T	T	.	.	.	.	.	G	.	.	.	.	.	.	.	.	.	.	.	.	.	.	.	.	.	.	.	.	.	.	.	.	.	.	.	.	.	C	.	-	-	-	-	-	-	-	-	-	-	-	-	-	-	-	-	-	-	-	-	-	-	-	-	-	-	-	-	-	-	-	-	-	-	-	-	-	-	-	-	-	-	-	.	.	.	.	.	G	.	.	.	.	.	.	.	.	.	.	.	.	.	.	.	.	.	T	.	.	.	.	.	.	.	.	.	.	.
THA468|NA	A3	.	T	.	.	.	.	.	.	C	.	.	.	.	.	.	.	.	.	T	C	C	T	T	G	T	T	A	G	T	T	.	.	.	.	.	G	.	.	.	.	.	.	.	.	.	.	.	.	.	.	.	.	.	.	.	.	.	.	.	.	.	.	.	.	.	C	.	-	-	-	-	-	-	-	-	-	-	-	-	-	-	-	-	-	-	-	-	-	-	-	-	-	-	-	-	-	-	-	-	-	-	-	-	-	-	-	-	-	-	-	.	.	.	.	.	G	.	.	.	.	.	.	.	.	.	.	.	C	C	A	.	.	.	T	.	.	.	.	.	.	.	.	.	.	.
K2550|NA	A3	.	T	.	.	.	.	.	.	C	.	.	.	.	.	.	.	.	.	T	C	C	T	T	G	T	C	A	G	T	T	.	.	.	.	.	G	.	.	.	.	.	.	.	.	.	.	.	.	.	.	.	.	.	.	.	.	.	.	.	.	.	.	.	.	.	C	.	-	-	-	-	-	-	-	-	-	-	-	-	-	-	-	-	-	-	-	-	-	-	-	-	-	-	-	-	-	-	-	-	-	-	-	-	-	-	-	-	-	-	-	.	.	.	.	.	G	.	.	.	.	.	.	.	.	.	.	.	.	.	.	.	.	.	T	.	.	.	.	.	.	.	.	.	.	.
K11|NA	A3	.	T	.	.	.	.	.	.	C	.	.	.	.	.	.	.	.	.	T	C	C	T	T	G	T	C	A	G	T	T	.	.	.	.	.	G	.	.	.	.	.	.	.	.	.	.	.	.	.	.	.	.	.	.	.	.	.	.	.	.	.	.	.	.	.	C	.	-	-	-	-	-	-	-	-	-	-	-	-	-	-	-	-	-	-	-	-	-	-	-	-	-	-	-	-	-	-	-	-	-	-	-	-	-	-	-	-	-	-	-	.	.	.	.	.	G	.	.	.	.	.	.	.	.	.	.	.	.	.	.	.	.	.	T	.	.	.	.	.	.	.	.	.	C	.
K3762|NA	A3	.	T	.	.	.	.	.	.	C	.	.	.	.	.	.	.	.	.	T	C	C	T	T	G	T	C	A	G	T	T	.	.	.	.	.	G	.	.	.	.	.	.	.	.	.	.	.	.	.	.	.	.	.	.	.	.	.	.	.	.	.	.	.	.	.	C	.	-	-	-	-	-	-	-	-	-	-	-	-	-	-	-	-	-	-	-	-	-	-	-	-	-	-	-	-	-	-	-	-	-	-	-	-	-	-	-	-	-	-	-	.	.	.	.	.	G	.	.	.	.	.	.	.	.	.	.	.	.	.	.	.	.	.	T	.	.	.	.	.	.	.	.	.	C	.
J739|NA	A3	.	T	.	.	.	.	.	.	C	.	.	.	.	.	.	.	.	.	T	C	C	T	T	G	T	C	A	G	T	T	.	.	.	.	.	G	.	.	.	.	.	.	.	.	.	.	.	.	.	.	.	.	.	.	.	.	.	.	.	.	.	.	.	.	.	C	.	-	-	-	-	-	-	-	-	-	-	-	-	-	-	-	-	-	-	-	-	-	-	-	-	-	-	-	-	-	-	-	-	-	-	-	-	-	-	-	-	-	-	-	.	.	.	.	.	G	.	.	.	.	.	.	.	.	.	.	.	.	.	.	.	.	.	T	.	.	.	.	.	.	.	.	.	.	.
QE470|NA	A3	.	T	.	.	.	.	.	.	C	.	.	.	.	.	.	.	.	.	T	C	C	T	T	G	T	C	A	G	T	T	.	.	.	.	.	G	.	.	.	.	.	.	.	.	.	.	.	.	.	.	.	.	.	.	.	.	.	.	.	.	.	.	.	.	.	C	.	-	-	-	-	-	-	-	-	-	-	-	-	-	-	-	-	-	-	-	-	-	-	-	-	-	-	-	-	-	-	-	-	-	-	-	-	-	-	-	-	-	-	-	.	.	.	.	.	G	.	.	.	.	.	.	.	.	.	.	.	.	.	.	.	.	.	T	.	.	.	.	.	.	.	.	.	.	.
QE1132|NA	A3	.	T	.	.	.	.	.	.	C	.	.	.	.	.	.	.	.	.	T	C	C	T	T	G	T	C	A	G	T	T	.	.	.	.	.	G	.	.	.	.	.	.	.	.	.	.	.	.	.	.	.	.	.	.	.	.	.	.	.	.	.	.	.	.	.	C	.	-	-	-	-	-	-	-	-	-	-	-	-	-	-	-	-	-	-	-	-	-	-	-	-	-	-	-	-	-	-	-	-	-	-	-	-	-	-	-	-	-	-	-	.	.	.	.	.	G	.	.	.	.	.	.	.	.	.	.	.	.	.	.	.	.	.	T	.	.	.	.	.	.	.	.	.	.	.
QV00961|HQ537758	A3	.	T	.	.	.	.	.	.	C	.	.	.	.	.	.	.	.	.	T	C	C	T	T	G	T	C	A	G	T	T	.	.	.	.	.	G	.	.	.	.	T	.	.	.	.	.	.	.	.	.	.	.	.	.	.	.	.	.	.	.	G	.	G	.	.	C	.	-	-	-	-	-	-	-	-	-	-	-	-	-	-	-	-	-	-	-	-	-	-	-	-	-	-	-	-	-	-	-	-	-	-	-	-	-	-	-	-	-	-	-	.	.	.	.	.	G	.	.	.	.	.	.	.	.	.	.	.	.	.	.	.	.	.	T	.	.	.	.	.	.	.	.	.	.	.
Z023|HQ537763	B1	.	.	.	.	.	.	C	.	.	T	.	A	.	.	.	.	.	G	-	-	-	-	-	-	-	-	-	-	-	-	-	.	.	G	.	.	C	.	.	.	.	.	.	.	.	.	.	.	.	.	.	.	C	.	.	.	.	G	A	.	.	.	.	.	.	.	.	-	-	-	-	-	-	-	-	-	-	-	-	-	-	-	-	-	-	-	-	-	-	-	-	-	-	-	-	-	-	-	-	-	-	-	-	-	-	-	-	-	-	-	.	.	.	.	.	.	.	.	.	.	.	.	.	.	.	.	.	.	.	.	.	G	.	T	.	A	.	.	.	.	.	.	.	.	.
BF134|HQ537762	B1	.	.	.	.	.	.	.	.	.	T	.	A	.	.	.	.	.	G	-	-	-	-	-	-	-	-	-	-	-	-	-	.	.	G	.	.	C	.	.	.	.	.	.	.	.	.	.	.	.	.	.	.	C	.	.	.	.	G	A	.	.	.	.	.	.	.	.	-	-	-	-	-	-	-	-	-	-	-	-	-	-	-	-	-	-	-	-	-	-	-	-	-	-	-	-	-	-	-	-	-	-	-	-	-	-	-	-	-	-	-	.	.	.	.	.	.	.	.	.	.	.	.	A	.	.	.	.	.	.	.	.	G	.	T	.	A	.	.	.	.	.	.	.	.	.
BF077|HQ537761	B1	.	.	.	.	.	.	.	.	.	T	.	A	.	.	.	.	.	G	-	-	-	-	-	-	-	-	-	-	-	-	-	.	.	G	.	.	C	.	.	.	.	.	.	.	.	.	.	.	.	.	.	.	C	.	.	.	.	G	A	.	.	.	.	.	.	.	A	-	-	-	-	-	-	-	-	-	-	-	-	-	-	-	-	-	-	-	-	-	-	-	-	-	-	-	-	-	-	-	-	-	-	-	-	-	-	-	-	-	-	-	.	.	A	.	.	.	.	.	.	.	.	.	.	.	.	.	.	.	.	.	.	G	A	T	.	A	.	.	.	.	.	.	.	.	.
051089|NA	B2	G	.	.	.	.	.	C	.	.	T	.	A	.	.	.	.	.	.	-	-	-	-	-	-	-	-	-	-	-	-	-	.	.	G	.	.	.	.	.	.	.	.	.	.	.	.	.	G	.	.	.	.	C	.	.	.	.	.	.	.	.	.	.	.	.	.	.	-	-	-	-	-	-	-	-	-	-	-	-	-	-	-	-	-	-	-	-	-	-	-	-	-	-	-	-	-	-	-	-	-	-	-	-	-	-	-	-	-	-	-	.	.	A	C	.	.	.	.	G	.	.	.	.	.	.	.	.	.	.	.	.	G	.	T	.	A	.	A	.	.	.	.	.	.	T
043998|NA	B2	G	.	.	.	.	.	C	.	.	T	.	A	.	.	.	.	.	.	-	-	-	-	-	-	-	-	-	-	-	-	-	.	.	G	.	.	.	.	.	.	.	.	.	.	.	.	.	G	.	.	.	.	C	.	.	.	.	.	.	.	.	.	.	.	.	.	.	-	-	-	-	-	-	-	-	-	-	-	-	-	-	-	-	-	-	-	-	-	-	-	-	-	-	-	-	-	-	-	-	-	-	-	-	-	-	-	-	-	-	-	.	.	A	C	.	.	.	.	G	.	.	.	.	.	.	.	.	.	.	.	.	G	.	T	.	A	.	A	.	.	.	.	.	.	T
RW937|HQ537764	B2	G	.	G	.	.	.	C	.	.	T	.	A	.	.	.	.	.	.	-	-	-	-	-	-	-	-	-	-	-	-	-	.	.	G	.	.	.	.	.	.	.	.	.	.	.	.	.	G	.	.	.	.	C	.	.	.	.	.	.	.	.	.	.	.	.	.	.	-	-	-	-	-	-	-	-	-	-	-	-	-	-	-	-	-	-	-	-	-	-	-	-	-	-	-	-	-	-	-	-	-	-	-	-	-	-	-	-	-	-	-	.	.	A	C	.	.	.	.	G	.	.	.	.	.	.	.	.	.	.	.	.	G	.	T	.	A	.	A	.	.	.	.	.	.	T
RW754|HQ537765	B2	G	.	.	.	.	.	C	.	.	T	.	A	.	.	.	.	.	.	-	-	-	-	-	-	-	-	-	-	-	-	-	.	.	G	.	.	.	.	.	.	.	.	.	.	.	.	.	G	.	.	.	.	C	.	.	.	.	.	.	.	.	.	.	.	.	.	.	-	-	-	-	-	-	-	-	-	-	-	-	-	-	-	-	-	-	-	-	-	-	-	-	-	-	-	-	-	-	-	-	-	-	-	-	-	-	-	-	-	-	-	.	.	.	C	.	.	.	.	G	.	.	.	.	.	.	.	.	.	.	.	.	G	.	T	.	A	.	A	.	.	.	.	.	.	T
Z094|HQ537777	C	.	.	.	.	.	-	-	C	.	.	.	A	.	.	.	.	.	.	-	-	-	-	-	-	-	-	-	-	-	-	-	.	.	G	.	.	.	.	C	G	.	.	.	.	.	.	G	.	.	.	G	.	.	.	.	.	.	.	.	.	.	.	.	.	.	.	.	-	-	-	-	-	-	-	-	-	-	-	-	-	-	-	-	-	-	-	-	-	-	-	-	-	-	-	-	-	-	-	-	-	-	-	-	-	-	-	-	-	-	-	.	.	A	.	.	.	.	.	.	.	.	.	.	.	.	G	.	.	.	.	.	G	.	T	.	A	.	A	.	.	.	.	.	.	.
062097|NA	C	.	.	.	.	.	-	-	C	.	.	.	A	.	.	.	.	.	.	-	-	-	-	-	-	-	-	-	-	-	-	-	.	.	G	.	.	.	.	C	.	.	.	.	.	.	.	G	.	.	.	G	.	.	.	.	.	.	.	.	.	.	.	.	.	.	.	.	-	-	-	-	-	-	-	-	-	-	-	-	-	-	-	-	-	-	-	-	-	-	-	-	-	-	-	-	-	-	-	-	-	-	-	-	-	-	-	-	-	-	-	.	.	A	.	.	.	.	.	.	.	.	.	.	.	.	.	.	.	.	.	.	G	.	T	.	A	.	A	.	.	.	.	.	.	.
050364|NA	C	.	.	.	.	.	-	-	C	.	.	.	A	.	.	.	.	.	.	-	-	-	-	-	-	-	-	-	-	-	-	-	.	.	G	.	.	.	.	C	.	.	.	.	.	.	.	G	.	.	.	G	.	.	.	.	.	.	.	.	.	.	.	.	.	.	.	.	-	-	-	-	-	-	-	-	-	-	-	-	-	-	-	-	-	-	-	-	-	-	-	-	-	-	-	-	-	-	-	-	-	-	-	-	-	-	-	-	-	-	-	.	.	A	.	.	.	.	.	.	.	.	.	.	.	.	.	.	.	.	.	.	G	.	T	.	A	.	A	.	.	.	.	.	.	.
RW792|HQ537775	C	.	.	.	.	.	-	-	C	.	.	.	A	.	.	.	.	.	.	-	-	-	-	-	-	-	-	-	-	-	-	-	.	T	G	.	.	.	.	C	G	.	.	.	.	.	.	G	.	.	.	G	.	.	.	.	.	.	.	.	.	.	.	.	.	.	.	.	-	-	-	-	-	-	-	-	-	-	-	-	-	-	-	-	-	-	-	-	-	-	-	-	-	-	-	-	-	-	-	-	-	-	-	-	-	-	-	-	-	-	-	.	.	A	.	.	.	.	.	G	.	.	C	.	.	.	.	.	.	.	.	.	G	.	T	C	A	.	A	.	.	.	.	.	.	.
RW644|HQ537776	C	.	.	.	.	.	-	-	C	.	.	.	A	.	.	.	.	.	.	-	-	-	-	-	-	-	-	-	-	-	-	-	.	.	G	.	.	.	.	C	G	.	.	.	.	.	.	G	.	.	.	G	.	.	.	.	.	.	.	.	.	.	.	.	.	.	.	.	-	-	-	-	-	-	-	-	-	-	-	-	-	-	-	-	-	-	-	-	-	-	-	-	-	-	-	-	-	-	-	-	-	-	-	-	-	-	-	-	-	-	-	.	.	A	.	.	.	.	.	G	.	.	C	.	.	.	.	.	.	.	.	.	G	.	T	C	A	.	A	.	.	.	.	.	.	.
QV13816|HQ537774	C	.	.	.	.	.	-	-	C	.	.	.	A	.	.	.	.	.	.	-	-	-	-	-	-	-	-	-	-	-	-	-	.	.	G	.	.	.	.	C	G	.	.	.	.	.	.	G	.	.	.	G	.	.	.	.	.	.	.	.	.	.	.	.	.	.	.	.	-	-	-	-	-	-	-	-	-	-	-	-	-	-	-	-	-	-	-	-	-	-	-	-	-	-	-	-	-	-	-	-	-	-	-	-	-	-	-	-	-	-	-	.	.	A	.	.	.	.	.	G	.	.	.	.	.	.	.	.	.	.	.	.	G	.	T	C	A	.	A	.	.	.	.	.	.	.
QV34982|HQ537772	C	.	.	.	.	.	-	-	C	.	.	.	A	.	.	.	.	.	.	-	-	-	-	-	-	-	-	-	-	-	-	-	.	.	G	.	.	.	.	C	G	.	.	.	.	.	.	G	.	.	.	G	.	.	.	.	.	.	.	.	.	.	.	.	.	.	.	.	-	-	-	-	-	-	-	-	-	-	-	-	-	-	-	-	-	-	-	-	-	-	-	-	-	-	-	-	-	-	-	-	-	-	-	-	-	-	-	-	-	-	-	.	.	A	.	.	.	.	.	G	.	.	.	.	.	.	.	.	.	.	.	.	G	.	T	C	A	.	A	.	.	.	.	.	.	.
054176|NA	C	.	.	.	.	.	-	-	C	.	.	.	A	.	.	.	.	.	.	-	-	-	-	-	-	-	-	-	-	-	-	-	.	.	G	.	.	.	.	C	G	.	.	.	.	.	.	G	.	.	.	G	.	.	.	.	.	.	.	.	.	.	.	.	.	.	.	.	T	T	T	G	T	T	A	T	G	C	C	A	A	A	A	T	A	T	G	T	C	T	T	G	T	A	A	A	A	G	T	G	A	C	T	C	A	C	T	A	A	C	A	.	.	A	.	.	.	.	.	G	.	.	.	.	.	.	.	.	.	.	.	.	G	.	T	C	A	.	A	.	.	.	.	.	.	.
QV03666|HQ537773	C	.	.	.	.	.	-	-	C	.	.	.	A	.	.	.	.	.	.	-	-	-	-	-	-	-	-	-	-	-	-	-	.	.	G	.	.	.	.	C	G	.	.	.	.	.	.	G	.	.	.	G	.	.	.	.	.	.	.	.	.	.	.	.	.	.	.	.	-	-	-	-	-	-	-	-	-	-	-	-	-	-	-	-	-	-	-	-	-	-	-	-	-	-	-	-	-	-	-	-	-	-	-	-	-	-	-	-	-	-	-	.	.	A	.	.	.	.	.	G	.	.	.	.	.	.	.	.	.	.	.	.	G	.	T	C	A	.	A	.	.	.	.	.	.	.
044033|NA	C	.	.	.	.	.	-	-	C	.	.	.	A	.	.	.	.	.	.	-	-	-	-	-	-	-	-	-	-	-	-	-	.	.	G	.	.	.	.	C	G	.	.	.	.	.	.	G	.	.	.	G	.	.	.	.	.	.	.	.	.	.	.	.	.	.	.	.	-	-	-	-	-	-	-	-	-	-	-	-	-	-	-	-	-	-	-	-	-	-	-	-	-	-	-	-	-	-	-	-	-	-	-	-	-	-	-	-	-	-	-	.	.	A	.	.	.	.	.	G	.	A	.	.	.	.	.	G	.	.	.	.	G	.	T	C	A	.	A	.	.	.	.	.	.	.
QV03858|HQ537766	D1	.	.	-	.	.	C	.	.	.	.	.	A	.	.	A	.	A	.	-	-	-	-	-	-	-	-	-	-	-	-	-	.	.	G	.	.	.	A	.	.	.	.	.	.	.	G	.	.	.	.	.	.	.	.	.	.	.	.	.	.	.	.	.	.	.	.	.	-	-	-	-	-	-	-	-	-	-	-	-	-	-	-	-	-	-	-	-	-	-	-	-	-	-	-	-	-	-	-	-	-	-	-	-	-	-	-	-	-	-	-	G	.	A	.	.	.	.	C	G	.	.	.	.	.	.	G	.	.	.	.	.	G	.	T	.	A	.	.	.	.	.	.	.	.	.
QV04732|HQ537767	D1	.	.	-	.	.	C	.	.	.	.	.	A	.	.	A	.	A	.	-	-	-	-	-	-	-	-	-	-	-	-	-	.	.	G	.	.	.	A	.	.	.	.	.	.	.	G	.	.	.	.	.	.	.	.	.	.	.	.	.	.	.	.	.	.	.	.	.	-	-	-	-	-	-	-	-	-	-	-	-	-	-	-	-	-	-	-	-	-	-	-	-	-	-	-	-	-	-	-	-	-	-	-	-	-	-	-	-	-	-	-	G	.	A	.	.	.	.	C	G	.	.	.	.	.	.	G	.	.	.	.	.	G	.	T	.	A	.	.	.	.	.	.	.	.	.
QV03841|HQ537768	D1	.	.	-	.	.	C	.	.	.	.	.	A	.	.	A	.	A	.	-	-	-	-	-	-	-	-	-	-	-	-	-	.	.	G	.	.	.	A	.	.	.	.	.	.	.	G	.	.	.	.	.	.	.	.	.	.	.	.	.	.	.	.	.	.	.	.	.	-	-	-	-	-	-	-	-	-	-	-	-	-	-	-	-	-	-	-	-	-	-	-	-	-	-	-	-	-	-	-	-	-	-	-	-	-	-	-	-	-	-	-	G	.	A	.	.	.	.	C	G	.	.	.	.	.	.	G	.	.	.	.	.	G	.	T	.	A	.	.	.	.	.	.	.	.	.
P138|NA	D1	.	.	-	.	.	C	.	.	.	.	.	A	.	.	A	.	A	.	-	-	-	-	-	-	-	-	-	-	-	-	-	.	.	G	C	.	.	A	.	.	.	.	.	.	.	G	.	C	.	.	.	.	.	.	.	.	.	.	.	.	.	.	.	.	.	.	.	-	-	-	-	-	-	-	-	-	-	-	-	-	-	-	-	-	-	-	-	-	-	-	-	-	-	-	-	-	-	-	-	-	-	-	-	-	-	-	-	-	-	-	.	.	A	.	.	.	.	C	G	.	.	.	.	.	T	.	.	.	.	.	.	G	.	T	.	A	.	.	.	.	.	.	.	.	.
064436|NA	D2	.	.	-	.	.	C	.	.	.	.	.	A	.	.	A	A	A	.	-	-	-	-	-	-	-	-	-	-	-	-	-	.	.	G	.	.	.	A	.	.	.	A	.	.	A	G	.	.	.	.	.	.	C	.	.	.	.	.	.	.	.	.	.	A	.	.	.	-	-	-	-	-	-	-	-	-	-	-	-	-	-	-	-	-	-	-	-	-	-	-	-	-	-	-	-	-	-	-	-	-	-	-	-	-	-	-	-	-	-	-	.	.	A	.	.	.	.	C	G	.	.	.	.	.	.	.	.	.	.	.	.	G	.	T	.	A	.	.	.	.	.	.	.	.	.
051402|NA	D2	.	.	-	.	.	C	.	.	.	.	.	A	.	.	A	A	A	.	-	-	-	-	-	-	-	-	-	-	-	-	-	.	.	G	.	.	.	A	.	.	.	A	.	.	A	G	.	.	.	.	.	.	C	.	.	.	.	.	.	.	.	.	.	A	.	.	.	-	-	-	-	-	-	-	-	-	-	-	-	-	-	-	-	-	-	-	-	-	-	-	-	-	-	-	-	-	-	-	-	-	-	-	-	-	-	-	-	-	-	-	.	.	A	.	.	.	.	C	G	.	.	.	.	.	.	.	.	.	.	.	.	G	.	T	.	A	G	.	.	.	.	.	.	.	.
RW841|HQ537769	D2	.	.	-	.	.	C	.	.	.	.	.	A	.	.	A	.	A	.	-	-	-	-	-	-	-	-	-	-	-	-	-	.	.	G	.	.	.	A	.	.	.	A	.	.	A	G	.	.	.	.	.	.	C	.	.	.	.	.	.	.	.	.	.	A	.	.	.	-	-	-	-	-	-	-	-	-	-	-	-	-	-	-	-	-	-	-	-	-	-	-	-	-	-	-	-	-	-	-	-	-	-	-	-	-	-	-	-	-	-	-	.	.	A	.	.	.	.	C	G	.	.	C	.	.	.	.	.	.	.	.	.	G	.	T	.	A	.	.	.	.	.	.	.	.	.
RW697|HQ537770	D2	.	.	-	.	.	C	.	.	.	.	.	A	.	.	A	.	A	.	-	-	-	-	-	-	-	-	-	-	-	-	-	.	.	G	.	.	.	A	.	.	.	A	.	.	A	G	.	.	.	.	.	.	C	.	.	.	.	.	.	.	.	.	.	A	.	.	.	-	-	-	-	-	-	-	-	-	-	-	-	-	-	-	-	-	-	-	-	-	-	-	-	-	-	-	-	-	-	-	-	-	-	-	-	-	-	-	-	-	-	-	.	.	A	.	.	.	.	C	G	.	.	.	.	.	.	.	.	.	.	.	.	G	.	T	.	A	.	.	.	.	.	.	.	.	.
RW63|HQ537771	D2	.	.	-	.	.	C	.	.	.	.	.	A	.	.	A	.	A	.	-	-	-	-	-	-	-	-	-	-	-	-	-	.	.	G	.	.	.	A	.	.	.	A	.	.	A	G	.	.	.	.	.	.	.	.	.	.	.	.	.	.	.	.	.	A	.	.	.	-	-	-	-	-	-	-	-	-	-	-	-	-	-	-	-	-	-	-	-	-	-	-	-	-	-	-	-	-	-	-	-	-	-	-	-	-	-	-	-	-	-	-	.	.	A	.	.	.	.	C	.	.	.	.	.	.	.	.	.	.	.	.	.	G	.	T	.	A	.	.	.	.	.	.	.	.	.

APOBEC3-induced change	
Amino acid site (prototype)	
PPH58|D90400	A1
SC165|FJ385265	A1
SC147|FJ385263	A1
SC100|FJ385261	A1
LZCC86|EU918765	A1
SC144|FJ385264	A1
SC101|FJ385262	A1
SC174|FJ385266	A1
SC185|FJ385267	A1
TW60|NA	A1
CNZJ3|KC860270	A1
QE190|NA	A1
SC78|FJ385268	A1
058771|NA	A1
047402|NA	A1
QV00861|HQ537755	A2
K1712|NA	A2
K3505|NA	A2
JP0221|AB819275	A2
QV03554|HQ537754	A2
JP1352|AB819278	A2
052265|NA	A2
051406|NA	A2
RW791|HQ537753	A2
CNZJ1|KC860269	A2
K20|NA	A2
K38|NA	A2
K643|NA	A2
JP0891|AB819277	A2
CNZJ2|KC860271	A2
K53|NA	A2
K2118|NA	A2
K421|NA	A2
JP0302|AB819276	A2
K25|NA	A2
QV15606|HQ537752	A2
36A|KU298920	A2
TJ18|GQ472850	A2
QE1538|NA	A2
QV32351|HQ537757	A3
QV15563|HQ537756	A3
AS347|HQ537760	A3
K2546|NA	A3
K8|NA	A3
K613|NA	A3
K191|NA	A3
K1374|NA	A3
JP1870|AB819279	A3
K34|NA	A3
K3823|NA	A3
K99|NA	A3
K2277|NA	A3
K64|NA	A3
K43|NA	A3
AS405|HQ537759	A3
QE150|NA	A3
THA468|NA	A3
K2550|NA	A3
K11|NA	A3
K3762|NA	A3
J739|NA	A3
QE470|NA	A3
QE1132|NA	A3
QV00961|HQ537758	A3
Z023|HQ537763	B1
BF134|HQ537762	B1
BF077|HQ537761	B1
051089|NA	B2
043998|NA	B2
RW937|HQ537764	B2
RW754|HQ537765	B2
Z094|HQ537777	C
062097|NA	C
050364|NA	C
RW792|HQ537775	C
RW644|HQ537776	C
QV13816|HQ537774	C
QV34982|HQ537772	C
054176|NA	C
QV03666|HQ537773	C
044033|NA	C
QV03858|HQ537766	D1
QV04732|HQ537767	D1
QV03841|HQ537768	D1
P138|NA	D1
064436|NA	D2
051402|NA	D2
RW841|HQ537769	D2
RW697|HQ537770	D2
RW63|HQ537771	D2

Table S3. Ancestral codon mutation of each lineage and sublineage using a maximum likelihood regression model.

Branch number	Ancestral codon mutation	nt site
Br1 (B/C/D)	E6, 86 GAC (D) 0.626 -> GAA (E) 1.000 *	367
	E7, 74 ACA (T) 0.614 -> GCA (A) 0.999 *	793
	E7, 75 ACC (T) 1.000 -> ACT (T) 1.000	801
	E2, 60 CCG (P) 1.000 -> CCT (P) 1.000	2932
	E2, 272 GTG (V) 1.000 -> GTT (V) 1.000	3571
	E2, 332 GAA (E) 1.000 -> GAG (E) 1.000	3751
	E2, 337 CTG (L) 0.759 -> ATG (M) 1.000 *	3764
	E4, 80 TGT (C) 0.500 -> TTT (F) 1.000 *	3571
	E5, 22 CCA (P) 1.000 -> CCG (P) 1.000	3957
Br2 (B2/C/D)	E7, 89 ACC (T) 1.000 -> ACT (T) 1.000	840
	L2, 33 CCC (P) 1.000 -> CCA (P) 1.000	4342
	L2, 89 CGT (R) 1.000 -> CGC (R) 1.000	4510
	L2, 100 TTG (L) 1.000 -> TTA (L) 1.000	4543
	L1, 150 TTC (F) 0.967 -> TTA (L) 0.973 *	6014
Br3 (B1)	E6, 32 GAA (E) 1.000 -> CAA (Q) 1.000 *	203
	E7, 63 GGC (G) 0.899 -> AGC (S) 1.000 *	760
	E1, 207 GCT (A) 1.000 -> GCG (A) 1.000	1503
	E1, 487 TTA (L) 1.000 -> CTA (L) 1.000	2341
	E1, 628 ATC (I) 1.000 -> GTC (V) 1.000 *	2764
	E2, 4 ATA (I) 1.000 -> ATG (M) 1.000 *	2764
	E2, 67 ACT (T) 1.000 -> ACC (T) 1.000	2953
	E2, 265 TGC (C) 1.000 -> TGT (C) 1.000	3550
	E2, 272 GTT (V) 1.000 -> GTC (V) 1.000	3571
	E4, 73 GCA (A) 1.000 -> GTA (V) 1.000 *	3550
	E4, 80 TTT (F) 1.000 -> TCT (S) 1.000 *	3571
	L2, 18 TAC (Y) 1.000 -> TAT (Y) 1.000	4297
	L2, 85 TTA (L) 1.000 -> TTG (L) 1.000	4498
	L2, 219 TCT (S) 1.000 -> TCG (S) 1.000	4900
	L2, 222 GTG (V) 1.000 -> GTA (V) 1.000	4909
	L2, 351 ACT (T) 0.998 -> AAT (N) 1.000 *	5295
	L2, 406 CTT (L) 0.997 -> TTT (F) 1.000 *	5459
	L1, 34 GAG (E) 1.000 -> GAA (E) 1.000	5666
	L1, 79 AAA (K) 1.000 -> AAG (K) 1.000	5801
	L1, 136 AGG (R) 1.000 -> AGA (R) 1.000	5972
	L1, 152 AAA (K) 1.000 -> AAG (K) 1.000	6020
	L1, 292 AAA (K) 1.000 -> AAG (K) 1.000	6440
	L1, 505 CTA (L) 1.000 -> CTG (L) 1.000	7079
	L1, 508 TCG (S) 1.000 -> TCA (S) 1.000	7088
Br4 (C/D)	E6, 97 AAT (N) 1.000 -> GAT (D) 0.933 *	398
	E1, 181 ACT (T) 1.000 -> AGT (S) 0.999 *	1424
	E1, 289 AAT (N) 1.000 -> AAC (N) 1.000	1749
	E5, 13 TTT (F) 1.000 -> TTC (F) 1.000	3930
	E5, 52 TTC (F) 1.000 -> TTT (F) 1.000	4047
	L2, 184 TCC (S) 1.000 -> TCG (S) 1.000	4795
	L2, 450 AGC (S) 1.000 -> AGT (S) 1.000	5593

Table S3. (cont.)

Branch number	Ancestral codon mutation	nt site
	L1, 10 GCT (A) 0.998 -> GTT (V) 0.981 *	5593
	L1, 325 ATG (M) 0.755 -> ATA (I) 0.895 *	6539
	L1, 484 CTA (L) 1.000 -> CTG (L) 1.000	7016
Br5 (B2)	E1, 286 TTA (L) 1.000 -> CTA (L) 1.000	1738
	E1, 511 CTA (L) 0.999 -> ATA (I) 0.998 *	2413
	E2, 269 GGG (G) 1.000 -> GGC (G) 1.000	3562
	E4, 77 GGC (G) 1.000 -> GCC (A) 1.000 *	3562
	L2, 433 ACT (T) 1.000 -> ACG (T) 1.000	5542
	L1, 75 TAT (Y) 1.000 -> TAC (Y) 1.000	5789
	L1, 79 AAA (K) 1.000 -> AAG (K) 1.000	5801
	L1, 220 ATT (I) 0.999 -> GTT (V) 0.999 *	6222
	L1, 298 CCG (P) 1.000 -> CCT (P) 1.000	6458
	L1, 311 GTT (V) 0.996 -> GCT (A) 0.999 *	6496
	L1, 312 ATC (I) 1.000 -> ATT (I) 1.000	6500
Br6 (D)	E6, 46 GTA (V) 0.451 -> ATA (I) 1.000 *	245
	E6, 86 GAA (E) 0.960 -> GAC (D) 1.000 *	367
	E7, 63 GGC (G) 1.000 -> GAC (D) 0.961 *	761
	E1, 79 ATA (I) 0.998 -> AGA (R) 0.912 *	1118
	E1, 104 GCC (A) 1.000 -> GCT (A) 1.000	1194
	E1, 376 GCA (A) 1.000 -> GCC (A) 1.000	2010
	E1, 453 TTT (F) 1.000 -> TTC (F) 1.000	2241
	E1, 511 CTA (L) 0.999 -> ATA (I) 0.999 *	2413
	L2, 162 TCC (S) 1.000 -> TCA (S) 1.000	4729
	L1, 85 AAA (K) 1.000 -> AAG (K) 1.000	5819
	L1, 298 CCG (P) 1.000 -> CCA (P) 1.000	6458
	L1, 482 GCA (A) 1.000 -> GCG (A) 1.000	7010
	L1, 505 CTA (L) 1.000 -> CTC (L) 1.000	7079
Br7 (C)	E6, 26 ATC (I) 1.000 -> ATT (I) 1.000	187
	E6, 46 GTA (V) 0.451 -> CTA (L) 1.000 *	245
	E7, 93 CCT (P) 1.000 -> CCC (P) 1.000	852
	E1, 170 GTA (V) 0.997 -> GCA (A) 1.000 *	1391
	E1, 180 AAT (N) 0.999 -> ACT (T) 1.000 *	1421
	E1, 334 GAT (D) 0.999 -> GAG (E) 1.000 *	1884
	L1, 99 GTC (V) 1.000 -> GTG (V) 1.000	5861
	L1, 125 TTG (L) 1.000 -> TTA (L) 1.000	5939
	L1, 158 ACC (T) 1.000 -> ACT (T) 1.000	6038
	L1, 159 AGT (S) 0.999 -> GGT (G) 1.000 *	6039
	L1, 163 CCC (P) 1.000 -> ACC (T) 1.000 *	6051
	L1, 292 AAA (K) 0.999 -> ACC (T) 1.000 *	6439
	L1, 296 GCT (A) 1.000 -> CCT (P) 1.000 *	6450
	L1, 299 GAT (D) 0.999 -> AAT (N) 1.000 *	6459
	L1, 311 GTT (V) 0.997 -> GGT (G) 1.000 *	6496
	L1, 376 AAG (K) 1.000 -> AAA (K) 1.000	6692
	L1, 378 GGT (G) 0.999 -> GAT (D) 1.000 *	6697
	L1, 383 GAT (D) 0.999 -> AAT (N) 1.000 *	6711
	L1, 508 TCG (S) 1.000 -> TCA (S) 1.000	7088

Table S3. (cont.)

Branch number	Ancestral codon mutation	nt site
Br8 (D2)	E6, 46 ATA (I) 1.000 -> ACA (T) 0.989 *	246
	E6, 97 GAT (D) 1.000 -> GAG (E) 0.923 *	400
	E7, 64 ACC (T) 0.969 -> GCC (A) 1.000 *	763
	E1, 58 ACT (T) 0.998 -> GCT (A) 0.999 *	1054
	E1, 450 TTA (L) 1.000 -> CTA (L) 1.000	2230
	L2, 84 CCT (P) 1.000 -> CCG (P) 1.000	4495
	L2, 341 CAA (Q) 1.000 -> CAC (H) 1.000 *	5266
Br9 (D1)	E7, 63 GAC (D) 0.961 -> CAC (H) 1.000 *	760
	E1, 62 GAA (E) 0.998 -> GAT (D) 1.000 *	1068
	L2, 29 CCT (P) 1.000 -> CCC (P) 1.000	4330
	L1, 412 GTA (V) 1.000 -> ATA (I) 1.000 *	6798
Br10 (A)	E1, 159 TCT (S) 1.000 -> TCG (S) 1.000	1359
	E1, 172 TGT (C) 0.582 -> AGT (S) 1.000 *	1396
	E1, 239 TCT (S) 1.000 -> TCC (S) 1.000	1599
	E1, 247 TTA (L) 1.000 -> CTA (L) 1.000	1621
	E1, 300 ATT (I) 1.000 -> ATC (I) 1.000	1782
	E1, 361 GAA (E) 0.612 -> GAT (D) 0.916 *	1965
	E1, 596 ACA (T) 0.580 -> AAA (K) 1.000 *	2669
	L2, 37 GGA (G) 1.000 -> GGC (G) 1.000	4354
	L2, 63 TCA (S) 1.000 -> TCG (S) 1.000	4432
	L2, 97 GTA (V) 1.000 -> GTG (V) 1.000	4534
	L2, 156 TCC (S) 1.000 -> TCT (S) 1.000	4711
	L2, 342 GAA (E) 0.968 -> CAA (Q) 1.000 *	5267
	L2, 377 CAC (H) 0.968 -> CAG (Q) 1.000 *	5374
	L2, 380 ATG (M) 0.968 -> CTG (L) 1.000 *	5381
	L1, 144 ATA (I) 0.988 -> GTA (V) 1.000 *	5994
	L1, 280 CAA (Q) 1.000 -> CAG (Q) 1.000	6404
	L1, 412 GTA (V) 0.979 -> ATA (I) 0.997 *	6798
	L1, 420 AAT (N) 0.987 -> GAT (D) 0.999 *	6822
	L1, 422 GAT (D) 0.988 -> AAT (N) 1.000 *	6828
Br11 (A3)	E7, 20 ACT (T) 1.000 -> ATT (I) 1.000 *	632
	E7, 63 GGC (G) 1.000 -> AGC (S) 1.000 *	760
	E1, 450 TTA (L) 1.000 -> CTA (L) 1.000	2230
	E2, 310 ACA (T) 1.000 -> ACG (T) 1.000	3685
	L2, 109 GAG (E) 1.000 -> GAA (E) 1.000	4570
	L2, 122 TCA (S) 1.000 -> TCG (S) 1.000	4609
	L2, 231 AAC (N) 1.000 -> ACC (T) 1.000 *	4935
	L2, 234 CAG (Q) 1.000 -> CAA (Q) 1.000	4945
	L2, 287 TTG (L) 1.000 -> TTA (L) 1.000	5104
	L2, 446 ATG (M) 1.000 -> CTG (L) 1.000 *	5579
	L1, 5 TTA (L) 0.999 -> TTC (F) 1.000 *	5579
	L1, 61 TAT (Y) 1.000 -> TAC (Y) 1.000	5747
Br12 (A1/2)	E1, 204 TTT (F) 1.000 -> TTC (F) 1.000	1494
	E1, 361 GAT (D) 0.916 -> GAC (D) 1.000	1965
	E1, 544 CAC (H) 1.000 -> CAT (H) 1.000	2514
	L1, 421 TCA (S) 1.000 -> TCC (S) 1.000	6827

Table S3. (cont.)

Branch number	Ancestral codon mutation		nt site
Br13 (A2)	E1, 22 GCG (A) 1.000 -> GCA (A) 1.000		948
	E1, 160 GAG (E) 0.998 -> CAG (Q) 1.000	*	1360
	E1, 300 ATC (I) 1.000 -> ATT (I) 1.000		1782
	E1, 486 TTT (F) 1.000 -> TTC (F) 1.000		2340
	E2, 61 TCA (S) 1.000 -> TCC (S) 1.000		2935
	E2, 231 CTC (L) 1.000 -> CTG (L) 1.000		3445
	E4, 39 TCG (S) 1.000 -> TGG (W) 1.000	*	3445
	E5, 20 TTG (L) 1.000 -> CTG (L) 1.000		3949
	E5, 33 TTG (L) 1.000 -> CTG (L) 1.000		3988
	L2, 292 TTA (L) 1.000 -> TTG (L) 1.000		5119
	L2, 300 CGT (R) 1.000 -> CGG (R) 1.000		5143
	L1, 284 AGA (R) 1.000 -> AGG (R) 1.000		6416
	L1, 290 GCT (A) 1.000 -> GCC (A) 1.000		6434
	L1, 359 GTG (V) 1.000 -> GTA (V) 1.000		6641
Br14 (A1)	E6, 66 TGT (C) 1.000 -> TGC (C) 1.000		307
	E7, 57 ACG (T) 1.000 -> ACT (T) 1.000		744
	E2, 265 TGC (C) 1.000 -> TGT (C) 1.000		3550
	E4, 73 GCA (A) 1.000 -> GTA (V) 1.000	*	3550
	L2, 234 CAG (Q) 1.000 -> CAA (Q) 1.000		4945
	L2, 287 TTG (L) 1.000 -> TTA (L) 1.000		5104
	L2, 341 CAA (Q) 1.000 -> CAG (Q) 1.000		5266
	L1, 325 ATG (M) 0.962 -> ATA (I) 0.956	*	6539

* Nonsynonymous changes (amino acid changes)

Reference:

1. **Kirii Y, Iwamoto S, Matsukura T.** 1991. Human papillomavirus type 58 DNA sequence. Virology **185:**424-427.
2. **Wu X, Zhang C, Feng S, Liu C, Li Y, Yang Y, Gao J, Li H, Meng S, Li L, Zhang Y, Hu X, Wu X, Lin L, Li X, Wang Y.** 2009. Detection of HPV types and neutralizing antibodies in Gansu province, China. J Med Virol **81:**693-702.
3. **Wu EQ, Zha X, Yu XH, Zhang GN, Wu YG, Fan Y, Ren Y, Kong LQ, Kong W.** 2009. Profile of physical status and gene variation of human papillomavirus 58 genome in cervical cancer. J Gen Virol **90:**1229-1237.
4. **Wu XL, Zhang CT, Zhu XK, Wang YC.** 2010. Detection of HPV types and neutralizing antibodies in women with genital warts in Tianjin City, China. Virol Sin **25:**8-17.
5. **Chen Z, Schiffman M, Herrero R, Desalle R, Anastos K, Segondy M, Sahasrabuddhe VV, Gravitt PE, Hsing AW, Burk RD.** 2011. Evolution and taxonomic classification of human papillomavirus 16 (HPV16)-related variant genomes: HPV31, HPV33, HPV35, HPV52, HPV58 and HPV67. PLoS One **6:**e20183.
6. **Kukimoto I, Maehama T, Sekizuka T, Ogasawara Y, Kondo K, Kusumoto-Matsuo R, Mori S, Ishii Y, Takeuchi T, Yamaji T, Takeuchi F, Hanada K, Kuroda M.** 2013. Genetic variation of human papillomavirus type 16 in individual clinical specimens revealed by deep sequencing. PLoS One **8:**e80583.
7. **Li Y, Wang X, Ni T, Wang F, Lu W, Zhu J, Xie X, Zheng ZM.** 2013. Human papillomavirus type 58 genome variations and RNA expression in cervical lesions. J Virol **87:**9313-9322.
8. **Siqueira JD, Alves BM, Prellwitz IM, Furtado C, Meyrelles AR, Machado ES, Seuanez HN, Soares MA, Soares EA.** 2016. Identification of novel human papillomavirus lineages and sublineages in HIV/HPV-coinfected pregnant women by next-generation sequencing. Virology **493:**202-208.
